# Steric and Electronic Effects on the Structure and Photophysical Properties of Hg(II) Complexes

**DOI:** 10.1021/acs.inorgchem.0c03640

**Published:** 2021-02-25

**Authors:** Francisco Sánchez-Férez, Joaquim Ma Rius-Bartra, Teresa Calvet, Mercè Font-Bardia, Josefina Pons

**Affiliations:** † Departament de Química, Universitat Autònoma de Barcelona, 08193 Bellaterra, Barcelona, Spain; ‡ Departament de Mineralogia, Petrologia i Geologia Aplicada, Universitat de Barcelona, Martí i Franquès s/n, 08028 Barcelona, Spain; § Unitat de Difracció de Raig-X, Centres Científics i Tecnològics de la Universitat de Barcelona (CCiTUB), Universitat de Barcelona, Solé i Sabarís, 1-3, 08028 Barcelona, Spain

## Abstract

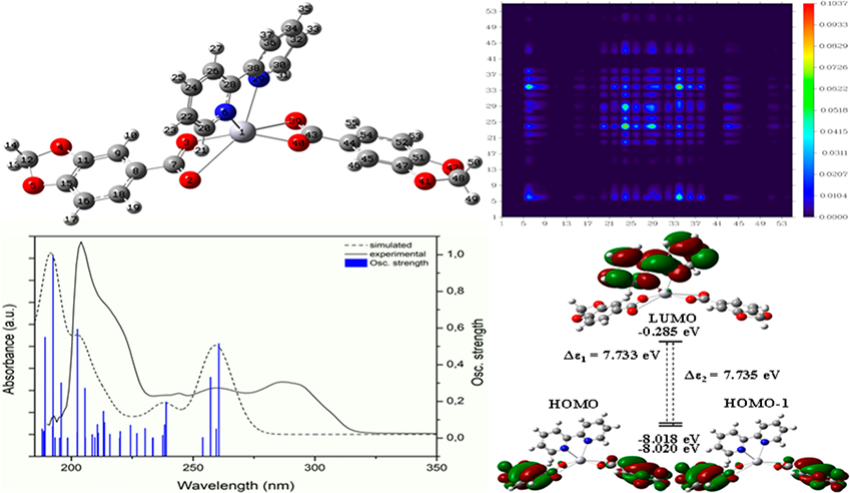

Since many factors influence the coordination around a metal center, steric and electronic effects of the ligands mainly determine the connectivity and, thus, the final arrangement. This is emphasized on Hg(II) centers, which have a zero point stabilization energy and, thus, a flexible coordination environment. Therefore, the unrestricted Hg(II) geometry facilitates the predominance of the ligands during the structural inception. Herein, we synthesized and characterized a series of six Hg(II) complexes with general formula (Hg(Pip)_2_(dPy)) (Pip = piperonylate, dPy = 3-phenylpyridine (3-phpy) (**1**), 4-phenylpyridine (4-phpy) (**2**), 2,2′-bipyridine (2,2′-bipy) (**3**), 1,10-phenanthroline (1,10-phen) (**4**), 2,2′:6′,2′-terpyridine (terpy) (**5**), or di(2-picolyl)amine (dpa) (**6**)). The elucidation of their crystal structures revealed the arrangement of three monomers (**3**, **5**, and **6**), one dimer (**4**), and two coordination polymers (**1** and **2**) depending on the steric requirements of the dPy and predominance of the ligands. Besides, the study of their photophysical properties in solution supported by TD-DFT calculations enabled us to understand their electronic effects and the influence of the structural arrangement on them.

## Abstract 2

Six Hg(II) complexes containing 1,3-benzodioxole-5-carboxylic acid and pyridine derivatives bearing *N*-, *N*^*N′*-, and *N*^*N*^*N*-donor sites have been characterized and their crystal structure elucidated. Steric crowding defined the predominance of the linkers in the structural arrangement, varying from monomers to coordination polymers. Their photophysical properties were analyzed through experimental and TD-DFT calculations, identifying the character and regions involved on each electronic transition. In addition, seclusion of the HOMO from the LUMO defined them as appropriate to selective band gap tuning.

## Introduction

Since the rise of crystal engineering,^1^ the development of structural design strategies has experienced a major breakthrough. The understanding and use of either supramolecular interactions^2,3^ or coordination bonds^4,5^ and their synergy in the crystal packing has led to the formation of solids with desired physical and chemical properties.^6^

Within this frame, the selection of the appropriate metal ion as well as the linkers will determine the molecular array, the intermolecular interactions, and, thus, the final crystal packing. While metal ions act as nodes and mainly determine the dimensionality through its geometry, modulation of the organic linkers (flexibility, length, or symmetry) drives the assembly of different architectures and therefore functionalities.^7−10^

Among all of the potential metal nodes, those belonging to group 12 with a d^10^ electronic configuration, and therefore zero crystal field stabilization energy, stand out for their flexible coordination environment and wide range of geometries,^11,12^ usually presenting severe distortions. They enable metal–ligand rearrangement during the structural inception, which gives rise to highly ordered networks.^13−15^ Furthermore, complexes with d^10^ metal nodes excel at presenting photoluminescence properties and, hence, have potential applications as fluorescence-emitting materials.^16^

It is essential to stress the lack of knowledge on the coordination behavior and photophysics of Hg(II) nodes. Even though Hg(II) can adopt coordination numbers from 2 to 10,^17^ it tends to form low coordinated linear structures,^18^ with the formation of Hg(II) coordination polymers being scarce compared to that of Zn(II) and Cd(II). In addition, Hg(II) complexes have potential applications in paper, paints, and cosmetics industry or in the production of manometers, mercury batteries, and energy efficient fluorescent light bulbs.

In terms of the linkers, carboxylic acids are a recognized class of versatile organic ligands for their potential coordination modes, from monodentate to bidentate bridging, which can form a large variety of structures. Besides, the addition of pyridine derivative ligands (dPy) with different denticity, electronic donor properties, conjugation, and planarity or steric requirements could result in diverse arrangements and functionalities.^19−22^

In this scenario, the photophysical properties of these systems could be improved by adding conjugated π aromatic linkers by tuning their band gap and also driving the crystal packing through C–H···π and π···π interactions.^23^ In particular, modulating the bite angle combined with a strong chelate effect using *N*^*N*-bidentate or *N*^*N*^*N*-tridentate chelate ligands has proven to be a determining factor of the final molecular geometry with a concomitant effect in the emission spectra.^24^

In a previous paper,^25^ our group studied and analyzed the structure and photophysics from the reaction of M(OAc)_2_ (M = Zn(II), Cd(II)) with 1,3-benzodioxole-5-carboxylic acid (piperonylic acid, HPip) and two pyridine derivative ligands (dPy = 3-phenylpyridine (3-phpy) and 4-phenylpyridine (4-phpy)), which resulted in the formation of two Zn(II) dimeric paddle-wheels ([Zn(μ-Pip)_2_(dPy)]_2_) and two Cd(II) dimers ([Cd(μ-Pip)(Pip)(dPy)_2_]_2_).

Recently, we have reported the reaction between Zn(II), Cd(II), Hg(II), and HPip, leading to the formation of one Zn(II) monomer ([Zn(Pip)_2_(H_2_O)_2_]) and three coordination polymers (two of Cd(II): [Cd(μ-Pip)_2_(H_2_O)]_*n*_ and [Cd_3_(μ-Pip)_6_(MeOH)_2_]_*n*_ and one of Hg(II): [Hg(μ-Pip)_2_]_*n*_]). All of them showed different topologies, coordination numbers, and modes, depending on the metal preferences.^26^

**Scheme 1:**
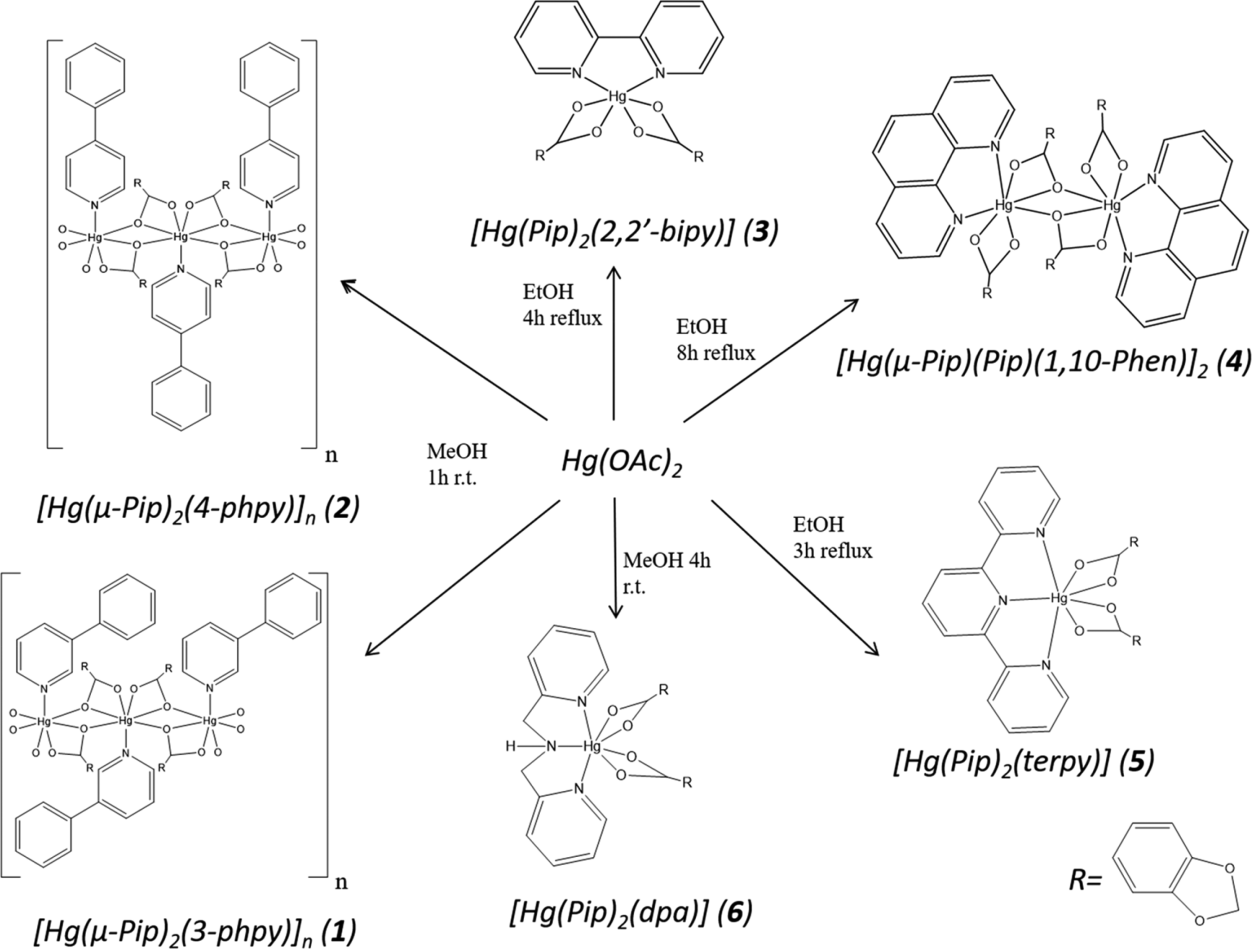
Outline of the Synthesis of Complexes **1**–**6**

Herein, we carried out the reaction of Hg(OAc)_2_ with HPip and a comprehensive range of pyridine derivative ligands (dPy). The choice of the dPy was made considering that (a) the increasing denticity of dPy combined with the chelate effect minimizes ligand dissociation at lower concentrations; (b) bulkier ligands avoid solvent attack and, therefore, solvent quenching by complexation as well as reduce geometric changes; and (c) to minimize aggregation and, thus, self-quenching by avoiding the presence of potential hydrogen bond donors.^27,28^ Therefore, the selected dPy were *N*-donor (3-phenylpyridine, 3-phpy; 4-phenylpyridine, 4-phpy), *N*^*N*-donor (2,2′-bipyridine, 2,2′-bipy; 1,10-phenanthroline, 1,10-phen), and *N*^*N*^*N*-donor sites (2,2′:6′,2′-terpyridine, terpy; 1-(2-pyridinyl)-*N*-(2-pyridinylmethyl)methanamine, di(2-picolyl)amine (dpa)). We successfully isolated six Hg(II) complexes: [Hg(μ-Pip)_2_(3-phpy)]_*n*_ (**1**), [Hg(μ-Pip)_2_(4-phpy)]_*n*_ (**2**), [Hg(Pip)_2_(2,2′-bipy)] (**3**), [Hg(μ-Pip)(Pip)(1,10-phen)]_2_·C_6_H_5_F (**4a**), [Hg(Pip)_2_(terpy)]·EtOH (**5**), and [Hg(Pip)_2_(dpa)]·^1^/_2_H_2_O·^1^/_2_MeOH (**6a**) (Scheme 1), which were fully characterized and their crystal structures elucidated. We further investigated their photophysical properties in MeOH solution, and we performed TD-DFT calculations to identify the electronic transitions and comprehend how the structural and electronic effect of the pyridine derivative influences the band gap, aggregation, and absorption spectrum.

## Experimental Section

### Chemical Risks

Hg(II) complexes are toxic, and any manipulation of the samples has to be carried out in the fume hood and wearing gloves.

### Materials and General Details

Hg(II) acetate (Hg(OAc)_2_), 1,3-benzodioxole-5-carboxylic acid (piperonylic acid, HPip), 3-phenylpyridine (3-phpy), 4-phenylpyridine (4-phpy), 2,2′-bipyridine (2,2′-bipy), 1,10-phenanthroline (1,10-phen), 2,2′:6′,2′-terpyridine (terpy), and di(2-picolyl)amine (dpa) ligands and methanol (MeOH), ethanol (EtOH), dichloromethane (CH_2_Cl_2_), diethyl ether (Et_2_O), and fluorobenzene (C_6_H_5_F, Fbz) solvents were purchased from Sigma-Aldrich. Deuterated chloroform (CDCl_3_) and deuterated dimethyl sulfoxide (dmso-*d*_6_) were used for the NMR experiments and were purchased from Eurisotop. All of them were used without further purification. Reactions and manipulation were carried out in air at room temperature (RT) for compounds **1**, **2**, and **6** and under reflux conditions for **3**–**5**. Thermal decomposition temperature (d.T.) was measured on a Stuart Melting Point Apparatus SMP30 (Cole-Parmer, U.K.) with a heating ramp of 2.0 °C/min in a temperature range from 20 to 210 °C. Elemental analyses (EA, C, H, N) were carried out on a Euro Vector 3100 instrument. HR-ESI^+^-MS measurements of complexes **1** and **2** in MeOH solution and **3**–**6** in MeOH/DMSO (80/20) were recorded in a MicroTOF-Q (Bruker Daltonics GmbH, Bremen, Germany) instrument equipped with an electrospray ionization source (ESI) in positive mode. Na^+^ ions come from the MeOH solvent which can contain <50 ppb. The conditions were those used in routine experiments. The nebulizer pressure was 1.5 bar, the desolvation temperature was 180 °C, the dry gas was at 6 L·min^–1^, the capillary counter-electrode voltage was 5 kV, and the quadrupole ion energy was 5.0 eV. Simultaneous TG/DTA determination of compound **5** was carried out in a Netzsch STA 409 instrument, with an aluminum oxide powder (Al_2_O_3_) crucible and heating at 5 °C·min^–1^ from 25 to 350 °C, under a nitrogen atmosphere with a flow rate of 80 mL·min^–1^. Al_2_O_3_ (PerkinElmer 0419-0197) was used as a standard. The FTIR-ATR spectra were recorded on a PerkinElmer spectrometer, equipped with a universal attenuated total reflectance (ATR) accessory with a diamond window in the range 4000–500 cm^–1^. ^1^H, ^13^C{^1^H}, and DEPT-135 NMR spectra were recorded on an NMR-FT Bruker 360 MHz spectrometer in CDCl_3_ or dmso-*d*_6_ solution at RT. All chemical shifts (δ) are given in ppm. The electronic spectra in solution of MeOH (**1**–**6**) were run on an Agilent HP 8453 UV–vis spectrophotometer with a quartz cell having a path length of 1 cm in the range 200–600 nm. Molar absorptivity values have been calculated as log(ε). Fluorescence measurements were carried out at 25 °C with a PerkinElmer LS 55 50 Hz fluorescence spectrometer using a 1 cm quartz cell, in MeOH solution. The samples were excited at their absorption maxima, and the emission was recorded between 200 and 440 nm. Dilution effects on data were corrected by Origin Pro 8 software.

#### Synthesis of Compound [Hg(μ-Pip)_2_(3-phpy)]_*n*_ (**1**)

To a solution of Hg(OAc)_2_ (100 mg, 0.314 mmol) in MeOH (30 mL), a solution of HPip (104 mg, 0.627 mmol) and 3-phpy (197 mg, 1.27 mmol) in MeOH (30 mL) was added dropwise under vigorous stirring at RT. Immediately, a white solid appeared. The reaction remained under stirring for 1 h. The solid obtained was filtered and washed with 10 mL of cold diethyl ether. Suitable crystals were obtained by recrystallization in MeOH for 15 days. Yield: 127 mg (59%). d.T. = 172 °C. Anal. Calcd for C_27_H_19_HgNO_8_ (686.04 g mol^–1^): C, 47.27; H, 2.79; N, 2.04. Found: C, 46.99; H, 2.84; N, 1.97%. HR-MS (ESI^+^, MeOH): *m*/*z* (%) = 156.0813 (100%) (calcd for [3-phpy + H]^+^ = 156.0808); 554.9998 (100%) (calcd for {[Hg(Pip)_2_] + Na}^+^ = 554.9977); 1085.0047 (77%) (calcd for {[Hg(Pip)_2_]_2_ + Na}^+^ = 1085.0046). FTIR-ATR (wavenumber, cm^–1^): 3090(w) [ν(CH)]_ar_, 3035(w) [ν(CH)]_ar_, 2918(w) [ν(CH)]_al_, 1628(w), 1604(w) [ν_as_(COO)], 1555(m) [ν_as_(COO)], 1521(m), 1504(w), 1487(s) [ν(C=C), ν(C=N)], 1475(m), 1461(m), 1437(s) [ν_s_(COO)], 1416(m), 1372(s) [δ(C=C), δ(C=N)], 1343(s), 1258(s), 1238(s), 1205(m), 1189(m), 1167(m), 1131(m), 1112(s) [ν(C—O—C)], 1072(m), 1032(s) [δ_ip_(C—H)], 934 (m), 921(m), 882(s), 839(w), 817(w), 804(m) [δ_oop_(C—H)], 772(s) [δ_oop_(C—H)], 753(s) [δ_oop_(C—H)], 721(m), 697(s), 680(s), 656(m), 625(m). ^1^H NMR (360 MHz, CDCl_3_, 298 K): δ = 6.06 [4H, s, O—C*H*_2_—O], 6.89 [2H, d, ^3^*J* = 8.2 Hz, O_2_C—CH—C***H***], 7.52 [8H, m, O_2_C—C***H***—CO + *m*-***H***_py(3-Phpy)_ + ***H***_ph(3-Phpy_)], 7.76 [2H, d, ^3^*J* = 8.2 Hz, O_2_C—C***H***—CH], 8.01 [1H, ddd, ^3^*J* = 7.9 Hz, ^4^*J* = 2.3 Hz, ^4^*J* = 1.7 Hz, *p*-***H***_py(3-Phpy)_], 8.64 [1H, dd, ^*3*^*J* = 5.4, ^4^*J* = 1.7 Hz, *o*-***H***_py(3-Phpy)_—CH], 8.89 [1H, d, ^4^*J* = 2.1, *o*-***H***_py(3-Phpy)_—C]. ^13^C{^1^H} NMR (360 MHz; dmso-*d*_6_; 298 K): δ = 169.20 [O_2_***C***—C], 150.57 [O_2_C—C—(CH)_2_—***C***], 148.89 [N—***C***H—C], 148.05 [N—***C***H—CH], 147.49 [O_2_C—C—CH—***C***], 137.05 [N—CH—C—***C***], 136.42 [N—CH—***C***], 135.52 [N—(CH)_2_—***C***H], 129.59 [C—C—CH—***C***H—CH or C—C—(CH)_2_—***C***H]_3-phpy_, 128.74 [C—C—(CH)_2_—***C***H or C—C—CH—***C***H—CH]_3-phpy_, 127.58 [O_2_C—***C***], 127.26 [C—C—***C***H]_3-phpy_, 125.54 [O_2_C—C—***C***H—CH], 124.80 [N—CH—***C***H], 109.91 [O_2_C—C—***C***H—C], 108.18 [O_2_C—C—CH—***C***H], 102.02 [O—***C***H_2_—O]. UV–vis (MeOH) λ_max_ (ε) = 212 nm (4.52); 258 nm (4.43); 278 nm (4.13); 293 nm (4.19).

#### Synthesis of Compound [Hg(μ-Pip)_2_(4-phpy)]*_n_* (**2**)

To a solution of Hg(OAc)_2_ (95.4 mg, 0.299 mmol) in MeOH (30 mL), a solution of HPip (103 mg, 0.623 mmol) and 4-phpy (191 mg, 1.23 mmol) in MeOH (30 mL) was added dropwise under vigorous stirring at RT. Immediately, a white solid appeared. The reaction remained under stirring for 1 h. The solid obtained was filtered and washed with 10 mL of cold diethyl ether. Suitable crystals were obtained by recrystallization in MeOH for 18 days. Yield: 109 mg (53%). d.T. = 171 °C. Anal. Calcd for C_27_H_19_HgNO_8_ (686.04 g mol^–1^): C, 47.27; H, 2.79; N, 2.04%. Found: C, 46.99; H, 2.64; N, 1.98%. HR-MS (ESI^+^, MeOH): *m*/*z* (%) = 156.0822 (100%) (calcd for [4-phpy·H]^+^ = 156.0761); 554.9990 (100%) (calcd for {[Hg(Pip)_2_] + Na}^+^ = 554.9977); 1085.0045 (71%) (calcd for {[Hg(Pip)_2_]_2_ + Na}^+^ = 1085.0046). FTIR-ATR (wavenumber, cm^–1^): 3073(w) [ν(CH)]_ar_, 2907(w) [ν(CH)]_al_, 1614(w) [ν_as_(COO)], 1560(m), 1550(m) [ν_as_(COO)], 1502(m), 1485(m) [ν(C=C), ν(C=N)], 1433(s) [ν_s_(COO)], 1373(s) [δ(C=C), δ(C=N)], 1336(s), 1256(s), 1227(s), 1168(m), 1109(m) [ν(C—O—C)], 1073(m), 1031(s) [δ_ip_(C—H)], 1012(w), 934 (w), 921(m), 878(m), 845(m), 821(m), 804(m) [δ_oop_(C—H)], 767(s) [δ_oop_(C—H)], 761(s) [δ_oop_(C—H)], 731(m), 719(m), 696(m), 677(s), 665(m), 621(m). ^1^H NMR (360 MHz, CDCl_3_, 298 K): δ = 6.02 [4H, s, O—C***H***_2_—O], 6.83 [2H, d, ^3^*J* = 8.4 Hz, O_2_C—CH—C***H***], 7.51 [5H, m, O_2_C—C***H***—CO + (*m*+*p*)-***H***_ph(4-phpy)_], 7.64 [2H, dd, ^3^*J* = 7.8 Hz, ^4^*J* = 1.7 Hz, *m*-***H***_py(4-Phpy)_], 7.68 [2H, m, *o*-***H***_ph(4-Phpy)_], 7.72 [2H, dd, ^3^*J* = 8.4 Hz, ^4^*J* = 1.7 Hz, O_2_C—C***H***—CH], 8.79 [2H, d, ^3^*J* = 4.7 Hz, *o*-***H***_py(4-Phpy)_]. ^13^C{^1^H} NMR (360 MHz; dmso-*d*_6_; 298 K): δ = 168.97 [O_2_***C***—C], 150.26 [N—***C***H], 150.17 [O_2_C—C—CH—CH—***C***], 148.67 [N—CH—CH—***C***], 147.14 [O_2_C—C—CH—***C***], 136.49 [N—(CH)_2_—C—***C***], 129.83 [C—C—(CH)_2_—***C***H or C—C—CH—***C***H—CH], 129.34 [C—C—(CH)_2_—***C***H or C—C—CH—***C***H—CH], 127.51 [O_2_C—***C***], 127.08 [C—C—***C***H], 125.16 [O_2_C—C—***C***H—CH], 122.12 [N—CH—***C***H], 109.59 [O_2_C—C—***C***H—C], 107.82 [O_2_C—C—CH—***C***H], 101.66 [O—***C***H_2_—O]. UV–vis (MeOH) λ_max_ (ε) = 205 nm (4.78); 259 nm (4.50); 293 nm (4.22).

#### Synthesis of Compound [Hg(Pip)_2_(2,2′-bipy)] (**3**)

To a colorless solution of Hg(OAc)_2_ (100 mg, 0.314 mmol) and HPip (104 mg, 0.628 mmol) in EtOH (25 mL), a solution of 2,2′-bipy (49.0 mg; 0.314 mmol) in MeOH (10 mL) was added dropwise under vigorous stirring and kept under reflux conditions for 4 h. A white powder precipitated. The solid obtained was filtered and washed with 5 mL of cold MeOH and 5 mL of cold diethyl ether. Suitable crystals were grown by recrystallization in MeOH for 5 days. Yield: 160 mg (75%). d.T. = 184 °C. Anal. Calcd for C_26_H_18_HgN_2_O_8_ (687.02 g·mol^–1^): C, 45.45; H, 2.64; N, 4.08. Found: C, 45.30; H, 2.58; N, 3.86%. HR-MS (ESI^+^, MeOH/DMSO): *m*/*z* (%) = 523.0570 (100%) (calcd for {[Hg(Pip)(2,2′-bipy)]}^+^ = 523.0579). FTIR-ATR (wavenumber, cm^–1^): 3107–3019 [ν(CH)]_ar_, 2999–2921(w) [ν(CH)]_al_, 1629(w), 1598(w), 1592(w), 1578(m), 1544(m) [ν_as_(COO)], 1503(m), 1490(m) [ν(C=C), ν(C=N)], 1470(m), 1432(m) [ν_s_(COO)], 1375(s) [δ(C=C), δ(C=N)], 1337(m), 1314(m), 1256(s), 1239(s), 1205(m), 1158(m), 1099(m) [ν(C—O—C)], 1076(w), 1032(s) [δ_ip_(C—H)], 1019(m), 981(w), 919 (m), 883(m), 819(m), 805(m) [δ_oop_(C—H)], 785(m) [δ_oop_(C—H)], 769(s) [δ_oop_(C—H)], 734(m) [δ_oop_(C—H)], 720(m) [δ_oop_(C—H)], 679(m), 651(m), 627(m), 586(m), 540(m). ^1^H NMR (360 MHz, dmso-*d*_6_, 298 K): δ = 6.07 [4H, s, O—C***H***_2_—O], 6.93 [2H, d, ^3^*J* = 8.2 Hz, O_2_C—CH—C***H***], 7.37 [2H, d, ^4^*J* = 0.9 Hz, O_2_C—C***H***—CO], 7.54 [2H, dd, ^3^*J* = 8.0 Hz, ^4^*J* = 1.2 Hz, O_2_C—C***H***—CH], 7.76 [2H, dd, ^3^*J* = 7.6 Hz, ^3^*J* = 5.3 Hz, *m*-***H***_py(2,2′-bipy)_], 8.20 [2H, t, ^3^*J* = 7.7 Hz, *p*-***H***_py(2,2′-bipy)_], 8.63 [2H, d, ^3^*J* = 7.6 Hz, *m*-***H***_py(2,2′-bipy)_—C], 8.87 [2H, d, ^3^*J* = 4.9 Hz, *o*-***H***_py(2,2′-bipy)_]. ^13^C{^1^H} NMR (360 MHz; dmso-*d*_6_; 298 K): δ = 169.31 [O_2_***C***—C], 150.84 [N—***C***—C], 150.06 [O_2_C—C—(CH)_2_—***C***], 149.73 [N—***C***H—CH], 147.11 [O_2_C—C—CH—***C***], 139.43 [N—(CH)_2_—***C***H], 128.20 [N—CH—***C***H], 125.87 [O_2_C—***C***], 124.91 [O_2_C—C—***C***H—CH], 122.11 [N—C—***C***H], 109.43 [O_2_C—C—***C***H—C], 107.77 [O_2_C—C—CH—***C***H], 101.63 [O—***C***H_2_—O]. UV–vis (MeOH) λ_max_ (ε) = 203 nm (4.69); 215 nm (4.66); 244 nm (4.18); 260 nm (4.25); 287 nm (4.29).

#### Synthesis of Compound [Hg(μ-Pip)(Pip)(1,10-phen)]_2_ (**4**)

To a colorless solution of Hg(OAc)_2_ (101 mg, 0.316 mmol) and HPip (105 mg, 0.633 mmol) in EtOH (25 mL), a solution of 1,10-phen (62.6 mg; 0.316 mmol) in MeOH (5 mL) was added dropwise under vigorous stirring and kept under reflux conditions for 8 h. A white powder precipitated. The solid obtained was filtered and washed with 5 mL of cold MeOH. Suitable crystals were grown by recrystallization in fluorobenzene for 6 days, obtaining the compound [Hg(μ-Pip)(Pip)(1,10-phen)]_2_·C_6_H_5_F (**4a**). The characterization corresponds to **4**. Yield: 172 mg (76%). d.T. = 190 °C. Anal. Calcd for C_56_H_36_Hg_2_N_4_O_16_ (1422.09 g·mol^–1^): C, 47.28; H, 2.55; N, 3.94. Found: C, 47.22; H, 2.43; N, 3.82%. HR-MS (ESI^+^, MeOH/DMSO): *m*/*z* (%) = 547.0576 (100%) (calcd for {[Hg(Pip)(1,10-phen)]}^+^ = 523.0580). FTIR-ATR (wavenumber, cm^–1^): 3064–3014 [ν(CH)]_ar_, 2903–2782(w) [ν(CH)]_al_, 1622(w) [ν_as_(COO)], 1603(w), 1585(m), 1574(m), 1543(m) [ν_as_(COO)], 1501(m), 1485(m) [ν(C=C), ν(C=N)], 1436(s) [ν_s_(COO)], 1367(s) [δ(C=C), δ(C=N)], 1319(s), 1260(s), 1240(s), 1163(m), 1142(w), 1107(m) [ν(C—O—C)], 1075(w), 1038(s) [δ_ip_(C—H)], 937(m), 917(m), 892(w), 879(w), 863(w), 853(m), 821(w), 802(m) [δ_oop_(C—H)], 768(s) [δ_oop_(C—H)], 725(s) [δ_oop_(C—H)], 680(m) [δ_oop_(C—H)], 640(w), 580(m). ^1^H NMR (360 MHz, CDCl_3_, 298 K): δ = 5.97 [4H, s, O—C***H***_2_—O], 6.76 [2H, d, ^3^*J* = 8.1 Hz, O_2_C—CH—C***H***], 7.57 [2H, d, ^4^*J* = 1.5 Hz, O_2_C—C***H***—CO], 7.71 [2H, dd, ^3^*J* = 8.1 Hz, ^4^*J* = 1.6 Hz, O_2_C—C***H***—CH], 8.0 [2H, s, ***H***_ph(1,10-phen)_], 8.01 [2H, m, *m*-***H***_py(1,10-phen)_]_,_ 8.53 [2H, dd, ^3^*J* = 8.2 Hz, ^4^*J* = 1.5 Hz, *p*-***H***_py(1,10-phen)_], 9.42 [2H, dd, ^3^*J* = 4.8 Hz, ^4^*J* = 1.7 Hz, *o*-***H***_py(1,10-phen)_]. ^13^C{^1^H} NMR (360 MHz; dmso-*d*_6_; 298 K): δ = 169.16 [O_2_***C***—C], 150.37 [N—***C***H], 149.49 [O_2_C—C—CH—CH—***C***], 146.74 [O_2_C—C—CH—***C***], 139.51 [N—(CH)_2_—***C***H], 138.47 [N—***C***], 128.89 [N—C—***C***], 128.66 [O_2_C—***C***], 127.07 [N—CH—***C***H], 125.47 [N—C—C—***C***H], 124.60 [O_2_C—C—***C***H—C], 109.32 [O_2_C—C—***C***H—CH], 107.39 [O_2_C—C—CH—***C***H], 101.28 [O—***C***H_2_—O]. UV–vis (MeOH) λ_max_ (ε) = 206 nm (4.78); 216 nm (5.03); 266 nm (5.05); 294 nm (4.93); 326 nm (3.68).

#### Synthesis of Compound [Hg(Pip)_2_(terpy)]·EtOH (**5**)

To a colorless solution of Hg(OAc)_2_ (100 mg, 0.316 mmol) and HPip (105 mg, 0.632 mmol) in EtOH (25 mL), a colorless solution of terpy (73.3 mg, 0.314 mmol) in EtOH (15 mL) was added dropwise under vigorous stirring, and the solution turned yellow. The reaction was kept under reflux conditions for 3 h. The solution was concentrated until half of the volume and cooled down in an ice bath until a yellowish powder precipitated. The solid was filtered and washed with 5 mL of cold methanol. Suitable crystals were obtained by slow evaporation of mother liquors on air for 12 days. Yield: 214 mg (89%). d.T. = 197 °C. Anal. Calcd for C_33_H_26_HgN_3_O_9_ (810.17 g·mol^–1^): C, 48.92; H, 3.36; N, 5.19. Found: C, 48.74; H, 3.23; N, 5.04%. HR-MS (ESI^+^, MeOH/DMSO): *m*/*z* (%) = 600.0837 (100%) (calcd for {[Hg(Pip)(terpy)]}^+^ = 600.0846). FTIR-ATR (wavenumber, cm^–1^): 3415(br) [ν(OH)], 3099–3016(w) [ν(CH)]_ar_, 2964–2789(w) [ν(CH)]_al_, 1624(w), 1591(w), 1575(w), 1534(m) [ν_as_(COO)], 1506(w), 1478(m) [ν(C=C), ν(C=N)], 1432(s) [ν_s_(COO)], 1366(s) [δ(C=C), δ(C=N)], 1347(s), 1312(s), 1253(s), 1196(w), 1160(m), 1114(w) [ν(C—O—C)], 1071(m), 1053(m), 1038(s) [δ_ip_(C—H)], 1010(m), 974 (w), 936(m), 916(m), 882(m), 852(w), 820(m), 805(m) [δ_oop_(C—H)], 776(s) [δ_oop_(C—H)], 740(w), 721(w), 680(m), 650(m), 636(w), 608(w), 586(m), 516(w), 507(w). ^1^H NMR (360 MHz; CDCl_3_; 298 K): δ = 3.72 [2H, q, ^3^*J* = 7.0 Hz, HO—C***H***_2_—CH_3_], 1.62 [1H, br, ***H***O—CH_2_—CH_3_], 1.24 [3H, t, ^3^*J* = 7.0 Hz, HO—CH_2_—C***H***_3_], 5.94 [4H, s, O—C***H***_2_—O], 6.72 [2H, d, ^3^*J* = 8.2 Hz, O_2_C—CH—C***H***], 7.52 [2H, d, ^4^*J* = 1.5 Hz, O_2_C—C***H***—CO], 7.65 [2H, dd, ^3^*J* = 8.1 Hz, ^4^*J* = 1.6 Hz, O_2_C—C***H***—CH], 7.66 [2H, dd, ^3^*J* = 8.2 Hz, ^4^*J* = 1.6 Hz, *m*-***H***_py-side(terpy)_], 8.0 [2H, td, ^3^*J* = 7.7 Hz, ^4^*J* = 1.8 Hz, *p*-***H***_py-side(terpy)_], 8.23 [5H, m, *m*-***H***_py-side(terpy)_ + (*m+p*)-***H***_py-center(terpy)_]_,_ 9.23 [2H, dd, ^3^*J* = 5.8 Hz, ^4^*J* = 1.6 Hz, *o*-***H***_py(terpy)_]. ^13^C{^1^H} NMR (360 MHz; dmso-*d*_6_; 298 K): δ = 169.38 [O_2_***C***—C], 150.65 [N—***C***H], 149.85 [O_2_C—C—(CH)_2_—***C***], 147.27 [O_2_C—C—CH—***C***], 140.30 [N—C—CH—***C***H_side_ and N—C—CH—***C***H_center_], 129.93 [N—CH—***C***H and N—C—***C***H_center_], 126.90 [O_2_C—***C***], 125.09 [O_2_C—C—***C***H—CH], 123.78 [N—***C***_side_ and N—***C***_center_], 123.22 [N—C—***C***H_side_], 109.95 [O_2_C—C—***C***H—C], 107.89 [O_2_C—C—CH—***C***H], 101.80 [O—***C***H_2_—O], 56.49 [HO—***C***H_2_], 19.02 [HO—CH_2_—***C***H_3_]. UV–vis (MeOH) λ_max_ (ε) = 205 nm (4.85); 253 nm (4.28); 282 nm (4.30); 298 nm (4.15); 322 nm (4.17).

#### Synthesis of Compound [Hg(Pip)_2_(dpa)] (**6**)

To a solution of Hg(OAc)_2_ (95.6 mg, 0.300 mmol) in MeOH (10 mL), a solution of HPip (103 mg, 0.618 mmol) in MeOH (20 mL) was added under stirring. Then, a yellow solution of dpa (59.8 mg, 0.300 mmol) in MeOH (5 mL) was added and stirred for 4 h. The resulting yellow solution was concentrated under vacuum until a dark yellow oil-like reaction crude was formed, which was dissolved in 10 mL of CH_2_Cl_2_, forced to precipitate with 15 mL of cold diethyl ether, and filtered (repeated twice). The final brownish powder was washed twice with 5 mL of cold diethyl ether. Recrystallization of the solid in MeOH and cooling down to 4 °C for 5 days resulted in suitable crystals of [Hg(Pip)_2_(dpa)]·^1^/_2_H_2_O·^1^/_2_MeOH (**6a**). The characterization corresponds to **6**. Yield: 118 mg (54%). d.T. = 178 °C. Anal. Calcd for C_28_H_23_HgN_3_O_8_ (730.09 g·mol^–1^): C, 46.06; H, 3.18; N, 5.76. Found: C, 46.14; H, 3.22; N, 5.80%. HR-MS (ESI^+^, MeOH/DMSO): *m*/*z* (%) = 566.0994 (100%) (calcd for {[Hg(Pip)(dpa)]}^+^ = 566.1001). FTIR-ATR (wavenumber, cm^–1^): 3312(w) [ν(NH)], 3069 (w) [ν(CH)]_ar_, 2902 (m) [ν(CH)]_al_, 1622(w), 1600(w), 1571(m) [ν_as_(COO)], 1545(m), 1502(w), 1485(m) [ν(C=C), ν(C=N)], 1433(s) [ν_s_(COO)], 1365–1321(s) [δ(C=C), δ(C=N)], 1285(m), 1252(s), 1237(s), 1159(m), 1126(w), 1104(m) [ν(C—O—C)], 1072(w), 1036(s) [δ_ip_(C—H)], 1012(m), 986(w), 935 (m), 920(m), 885(w), 880(w), 804(m) [δ_oop_(C—H)], 769(s) [δ_oop_(C—H)], 719(m) [δ_oop_(C—H)], 678(m), 638(w), 583(m), 537(w), 524(w), 505(w). ^1^H NMR (360 MHz; dmso-*d*_6_; 298 K): δ = 4.16 [4H, s, NH—C***H***_2_], 5.10 [1H, br, N***H***], 6.03 [4H, s, O—C***H***_2_—O], 6.87 [2H, d, ^3^*J* = 8.2 Hz, O_2_C—CH—C***H***], 7.37 [2H, br, *m*-***H***_py_], 7.51 [2H, d, ^3^*J* = 8.2 Hz, O_2_C—C***H***—CH], 7.55 [4H, m, O_2_C—C***H***—CO + *m*-***H***_py_—CH_2_], 7.97 [2H, t, ^3^*J* = 7.7 Hz, *p*-***H***_py_], 8.75 [2H, d, ^3^*J* = 5.3 Hz, *o*-***H***_py_]. ^13^C{^1^H} NMR (360 MHz; dmso-*d*_6_; 298 K): δ = 169.47 [O_2_***C***—C], 155.34 [N—***C***—CH_2_], 149.61 [O_2_C—C—(CH)_2_—***C***], 149.42 [N—***C***H], 147.25 [O_2_C—C—CH—***C***], 139.50 [N—(CH)_2_—***C***H], 130.98 [O_2_C—***C***], 124.96 [O_2_C—C—***C***H—CH], 124.58 [N—C—***C***H + N—CH—***C***H], 110.04 [O_2_C—C—***C***H—C], 107.85 [O_2_C—C—CH—***C***H], 101.74 [O—***C***H_2_—O], 50.77 [N—***C***H_2_]. UV–vis (MeOH) λ_max_ (ε) = 202 nm (4.17); 238 nm (4.03); 281 nm (3.88); 307 nm (3.99).

### X-ray Crystallography

Colorless needle-like (**1** and **2**), colorless prism-like (**3**, **4a**, and **6a**), and yellow needle-like (**5**) specimens were used for the X-ray crystallographic analysis. The X-ray intensity data were measured on a D8 Venture system equipped with a multilayer monochromator and a Mo microfocus (λ = 0.71073 Å). For **1**–**6a**, the frames were integrated with the Bruker SAINT Software package using a narrow-frame algorithm. For **1**, the integration of the data using a triclinic unit cell yielded a total of 17,255 reflections to a maximum θ angle of 23.27° (0.90 Å resolution), of which 3230 were independent (average redundancy 5.342, completeness = 99.5%, *R*_int_ = 2.12%, *R*_sig_ = 1.54%) and 3127 (96.81%) were greater than 2σ(|*F*|^2^). The calculated minimum and maximum transmission coefficients (based on crystal size) are 0.5894 and 0.7449. For **2**, the integration of the data using a triclinic unit cell yielded a total of 17,834 reflections to a maximum θ angle of 27.12° (0.78 Å resolution), of which 2591 were independent (average redundancy 6.883, completeness = 99.8%, *R*_int_ = 2.91%, *R*_sig_ = 1.73%) and 2437 (94.06%) were greater than 2σ(|*F*|^2^). The calculated minimum and maximum transmission coefficients (based on crystal size) are 0.5114 and 0.7461.

For **3**, the integration of the data using a monoclinic unit cell yielded a total of 22,978 reflections to a maximum θ angle of 30.56 (0.70 Å resolution), of which 3445 were independent (average redundancy 6.670, completeness = 98.6%, *R*_int_ = 4.64%, *R*_sig_ = 2.87%) and 3237 (93.96%) were greater than 2σ(|*F*|^2^). The calculated minimum and maximum transmission coefficients (based on crystal size) are 0.3609 and 0.7461. For **4a**, the integration of the data using a monoclinic unit cell yielded a total of 58,489 reflections to a maximum θ angle of 30.46° (0.70 Å resolution), of which 7936 were independent (average redundancy 7.370, completeness = 99.4%, *R*_int_ = 3.32%, *R*_sig_ = 2.02%) and 7288 (96.83%) were greater than 2σ(|*F*|^2^). The calculated minimum and maximum transmission coefficients (based on crystal size) are 0.5115 and 0.7461. For **5**, the integration of the data using a monoclinic unit cell yielded a total of 64,064 reflections to a maximum θ angle of 30.56° (0.70 Å resolution), of which 4497 were independent (average redundancy 14.248, completeness = 99.8%, *R*_int_ = 2.96%, *R*_sig_ = 1.35%) and 4274 (95.04%) were greater than 2σ(|*F*|^2^). The calculated minimum and maximum transmission coefficients (based on crystal size) are 0.6261 and 0.7461. For **6a**, the integration of the data using a triclinic unit cell yielded a total of 159,036 reflections to a maximum θ angle of 36.35° (0.60 Å resolution), of which 25,925 were independent (average redundancy 6.134, completeness = 99.3%, *R*_int_ = 3.86%, *R*_sig_ = 2.53%) and 23005 (88.74%) were greater than 2σ(|*F*|^2^). The calculated minimum and maximum transmission coefficients (based on crystal size) are 0.5108 and 0.7471.

The structures were solved using the Bruker SHELXTL software package and refined using SHELX (version-2018/3).^29^ For **1**, the final anisotropic full-matrix least-squares refinement on |*F*|^2^ with 334 variables converged at *R*_1_ = 1.18% for the observed data and *wR*_2_ = 4.68% for all data. For **2**, the final anisotropic full-matrix least-squares refinement on |*F*|^2^ with 128 variables converged at *R*_1_ = 4.59% for the observed data and *wR*_2_ = 13.46% for all data. For **3**, the final anisotropic full-matrix least-squares refinement on |*F*|^2^ with 168 variables converged at *R*_1_ = 1.96% for the observed data and *wR*_2_ = 4.93% for all data. For **4a**, the final anisotropic full-matrix least-squares refinement on |*F*|^2^ with 334 variables converged at *R*_1_ = 2.68% for the observed data and *wR*_2_ = 6.10% for all data. For **5**, the final anisotropic full-matrix least-squares refinement on |*F*|^2^ with 224 variables converged at *R*_1_ = 1.39% for the observed data and *wR*_2_ = 3.27% for all data. For **6a**, the final anisotropic full-matrix least-squares refinement on |*F*|^2^ with 755 variables converged at *R*_1_ = 1.87% for the observed data and *wR*_2_ = 4.33% for all data.

For **1**–**6a**, the final cell constants and volume are based upon the refinement of the *XYZ*-centroids of reflections above 20 σ(*I*). Data were corrected for absorption effects using the multiscan method (SADABS). Crystal data and relevant details of structure refinement for compounds **1**–**6a** are reported in Table 1 and Table 2. Complete information about the crystal structure and molecular geometry is available in CIF format via CCDC 2043891 (**1**), 2043892 (**2**), 2043890 (**3**), 2043893 (**4a**), 2043894 (**5**), and 2043895 (**6a**). Molecular graphics were generated with Mercury 4.2.0 software^30−32^ using the POV-Ray image package.^33^ The color codes for all of the molecular graphics are as follows: light gray (Hg), light blue (N), red (O), yellow (F), gray (C), and white (H).

**Table 1:** Crystal Structure Refinement Parameters for **1**–**3**

	**1**	**2**	**3**
empirical formula	C_27_H_19_HgNO_8_	C_27_H_19_HgNO_8_	C_26_H_18_HgN_2_O_8_
formula weight	686.02	686.02	687.01
*T* (K)	100(2)	100(2)	100(2)
wavelength (Å)	0.71073	0.71073	0.71073
system, space group	triclinic, *P̅*1	monoclinic, *C*2/*c*	monoclinic, *P*2/*c*
unit cell dimensions			
*a* (Å)	8.8386(3)	19.3137(6)	11.2979(6)
*b* (Å)	11.7440(5)	14.5623(4)	8.3924(5)
*c* (Å)	12.0985(5)	8.3428(2)	12.9283(8)
α (deg)	112.6490(10)	90	90
β (deg)	97.1300(10)	95.1020(10)	111.306(2)
γ (deg)	97.3600(10)	90	90
*V* (Å^3^)	1128.60(8)	2337.13(11)	1142.04(12)
*Z*	2	4	2
*D*_calc_ (g cm^3^)	2.019	1.950	1.998
μ (mm^–1^)	6.876	6.641	6.797
*F* (000)	664	1328	664
crystal size (mm^3^)	0.198 × 0.081 × 0.072	0.402 × 0.048 × 0.045	0.315 × 0.189 × 0.135
*hkl* ranges	–9 ≤ *h* ≤ 9	–24 ≤ *h* ≤ 24	–16 ≤ *h* ≤ 14
	–13 ≤ *k* ≤ 13	–18 ≤ *k* ≤ 18	–11 ≤ *k* ≤ 11
	–13 ≤ *l* ≤ 13	–10 ≤ *l* ≤ 9	–18 ≤ *l* ≤ 18
θ range (deg)	2.366–23.274	2.117–27.124	2.427–30.557
reflections collected/unique/[*R*_int_]	17254/3229/[*R*_int_] = 0.0212	17834/2591/[*R*_int_] = 0.0291	22978/3445/[*R*_int_] = 0.0464
completeness to θ (%)	99.5	99.8	99.1
absorption correction	semiempirical	semiempirical	semiempirical
max. and min. transmis.	0.7449 and 0.5894	0.7461 and 0.5114	0.7461 and 0.3609
refinement method	full-matrix least-squares on |*F*|^2^	full-matrix least-squares on |*F*|^2^	full-matrix least-squares on |*F*|^2^
data/restraints/parameters	3229/0/334	2591/4/128	3445/0/168
goodness of fit (GOF) on |*F|*^2^	1.104	1.169	1.044
final *R* indices [*I* > 2σ(*I*)]	*R*_1_ = 0.0114,	*R*_1_ = 0.0459,	*R*_1_ = 0.0196,
	*wR*_2_ = 0.0272	*wR*_2_ = 0.1238	*wR*_2_ = 0.0478
*R* indices (all data)	*R*_1_ = 0.0134	*R*_1_ = 0.0504,	*R*_1_ = 0.0218,
	*wR*_2_ = 0.0275	*wR*_2_ = 0.1346	*wR*_2_ = 0.0493
extinction coefficient	n/a	n/a	n/a
largest. diff. peak and hole (e Å^–3^)	0.318 and −0.587	2.485 and −1.987	0.635 and −1.564

**Table 2:** Crystal Structure Refinement Parameters for **4a**–**6a**

	**4a**	**5**	**6a**
empirical formula	C_62_H_41_FHg_2_N_4_O_16_	C_33_H_27_HgN_3_O_9_	C_57_H_52_Hg_2_N_6_O_18_
formula weight	1518.17	810.16	1510.22
*T* (K)	100(2)	100(2)	100(2)
wavelength (Å)	0.71073	0.71073	0.71073
system, space group	monoclinic *C*2/*c*	monoclinic, *C*2/*c*	triclinic, *P̅*1
unit cell dimensions			
*a* (Å)	28.224(4)	22.9864(8)	9.5659(10)
*b* (Å)	15.6228(19)	13.2575(5)	17.2009(19)
*c* (Å)	12.2354(14)	10.7806(3)	17.2979(19)
α (deg)	90	90	100.768(4)
β (deg)	103.270(6)	116.3960(10)	100.641(4)
γ (deg)	90	90	99.745(4)
*V* (Å^3^)	5250.9(11)	2942.79(17)	2686.1(5)
*Z*	4	4	2
*D*_calc_ (g cm^3^)	1.920	1.829	1.867
μ (mm^–1^)	5.926	5.294	5.792
*F* (000)	2952	1592	1480
crystal size (mm^3^)	0.296 × 0.128 × 0.067	0.124 × 0.064 × 0.030	0.321 × 0.184 × 0.120
*hkl* ranges	–40 ≤ *h* ≤ 40	–32 ≤ *h* ≤ 32	–15 ≤ *h* ≤ 15
	–22 ≤ *k* ≤ 22	–18 ≤ *k* ≤ 18	–28 ≤ *k* ≤ 28
	–17 ≤ *l* ≤ 17	–14 ≤ *l* ≤ 15	–28 ≤ *l* ≤ 28
θ range (deg)	2.578–30.463	3.073–30.556	2.466–36.354
reflections collected/unique/[*R*_int_]	58489/7936/[*R*_int_] = 0.0332	64074/4497/[*R*_int_] = 0.0296	159036/25925/[*R*_int_] = 0.0386
completeness to θ (%)	99.5	99.7	99.5
absorption correction	semiempirical	semiempirical	semiempirical
max. and min. transmis.	0.7461 and 0.5115	0.7461 and 0.6261	0.7471 and 0.5108
refinement method	full-matrix least-squares on |*F*|^2^	full-matrix least-squares on |*F*|^2^	full-matrix least-squares on |*F*|^2^
data/restraints/parameters	7936/6/448	4497/0/2244	25925/3/755
goodness of fit (GOF) on |*F|*^2^	1.137	1.081	1.049
final *R* indices [*I* > 2σ(*I*)]	*R*_1_ = 0.0268,	*R*_1_ = 0.0139,	*R*_1_ = 0.0187,
	*wR*_2_ = 0.0594	*wR*_2_ = 0.0320	*wR*_2_ = 0.0406
*R* indices (all data)	*R*_1_ = 0.0304,	*R*_1_ = 0.0160	*R*_1_ = 0.0242
	*wR*_2_ = 0.0610	*wR*_2_ = 0.0327	*wR*_2_ = 0.0433
extinction coefficient	n/a	n/a	n/a
largest. diff. peak and hole (e Å^–3^)	2.118 and −2.068	0.486 and −0.910	1.266 and −1.758

### Methodology and Computational Details

All of the calculations have been performed using Gaussian 09 software, version D.01.^34^ Since the chemistry of Hg(II) is still largely unexplored, there is a lack of systematic computational research to better understand experimental results, especially from an electronic perspective as in the field of photochemistry. It is worth mentioning that the reported examples of TD-DFT calculations with Hg(II) complexes are scarce and no previously reported data have been found regarding electronic transition calculations. Geometry optimization of the ground state (GS) and vertical absorptions from the electronically excited state (EES) for **1**–**5** have been done with density functional theory (DFT) and time-dependent DFT (TD-DFT), respectively, using the ωB97X-D^35,36^ functional (Supporting Information: Tables S3–S7 and Figures S37–S41). A correlation consistent basis set was used for the Hg(II) atom, the effective core potential CrenbL,^37^ while the 6-311G(2d,p)^38,39^ basis set was used with C, H, N, and O atoms. MeOH solvation effects were incorporated using the polarizable continuum model-linear response (PCM-LR).^40,41^ The frequencies were also computed for each optimized structure to ensure that the geometries corresponded to an energy minimum. All of the optimized geometries in MeOH solution are similar to their corresponding ones determined by the single crystal X-ray diffraction method. The most significant conformational changes are observed in **5**. For instance, this is reflected in its pyridyl–pyridyl torsion angles. Unfortunately, all of the efforts to reach the geometry energy minima of complex **6** were unsuccessful. The highest occupied molecular orbital (HOMO) and lowest unoccupied molecular orbital (LUMO) were first examined and the band gap energies calculated. For **1**–**5**, the first 60 vertical absorptions from the ground state to the excited states have been calculated and only the most probable transitions (higher *f* values) have been selected for the electronic analysis.

Electronic transitions are composed of multiple non-negligible contributions from molecular orbitals (MOs), and their representation and evaluation as canonical orbitals could be intricated. Therefore, the use of natural transition orbitals (NTOs)^42^ is a useful tool to identify and represent the molecular orbitals implied on the transition. The generation of NTOs is based on separately performing unitary transformation for occupied MOs and virtual MOs outstanding those pairs with predominant contributions, which makes the orbitals’ inspection much easier. NTOs of the selected transitions for **1**–**5** have been generated using an isovalue of 0.02. Likewise, the identification of each transition type—(i) charge transfer (CT), in which the transition leads to evident movement of charge density, or (ii) local excitation, where the electronic movement occurs in the same spatial region (LE)—is not straightforward, and it has been afforded by calculating the Δ*r* index^43^ (smaller Δ*r* values (<2.0) define likely LE modes, while higher values (>2.0) pertain to charge transfers) and mapping the transition density matrix (TDM) as a color filled 2D plot. The combination of both allows the electronic excitation mode to be established. The diagonal terms of the 2D plot highlight the primary atoms involved in the transition, and the spatial extent can be graphically evaluated. The Δ*r* index and TDM mapping have been calculated using Multiwfn 3.7 software.^44^

## Results and Discussion

### General Materials and Methods

Complexes **1**–**6** were prepared via combination of Hg(OAc)_2_, HPip, and the corresponding dPy. Reactions were performed in a 1:2:4 (Hg:Pip:dPy) molar ratio for compounds **1** and **2**, while **3**–**6** were synthesized in a 1:2:1 proportion. The different molar ratio between the syntheses of **1** and **2** and complexes **3**–**6** could lie in the different coordination modes of the ligands (monodentate in **1** and **2** and chelate in **3**–**6**). The synthesis of **1**, **2**, and **6** was done in MeOH at room temperature (RT), while the synthesis of **3**–**5** in EtOH was done under reflux conditions. Therefore, the synthesis of the complexes depends mainly on the molar ratio and on the reaction conditions. The corresponding crystals suitable for X-ray crystallographic analysis were grown via recrystallization in MeOH for **1**–**3** and **6**, in fluorobenzene for **4**, or by slow evaporation of mother liquors for **5**. Complexes **4** and **6** crystallized occluding solvent molecules, yielding **4a** and **6a**. As aforementioned, Hg(II) ions having a d^10^ electronic configuration entail a zero crystal field stabilization energy and enable an easier ligand rearrangement. Thus, the incorporation of the dPy ligands has led to different arrangements, coordination numbers, and geometries and the concomitant variation of their photophysical properties.

All of the six compounds were characterized by EA, FTIR-ATR, ^1^H and ^13^C{^1^H}, and DEPT-135 NMR spectroscopies and single crystal X-ray diffraction method. Compounds **1** and **2** were also characterized by HR-ESI^+^-MS in MeOH solution. In addition, the thermal stability of complex **5** was studied via TG/DTA determinations. Finally, the UV–vis and photoluminescence spectra of **1**–**6** were recorded in MeOH solution and their quantum yields calculated. The photophysical properties of **1**–**5** were analyzed using TD-DFT calculations via 2D color mapping of TDM supported by NTO analysis.

EA of all of the compounds agree with the proposal formula. Besides, ESI^+^-MS of complexes **1**–**6** have been recorded. For complexes **1** and **2**, the weaker monodentate Hg–*N* coordination bond of 3-phpy (**1**) or 4-phpy (**2**) is broken during the ESI^+^ fragmentation, resulting in the formation of fragments with *m*/*z* 156.0813 (100%) [3-phpy + H]^+^ and 156.0822 (100%) [4-phpy + H]^+^, respectively. As a consequence, the formation of {[Hg(Pip)_2_] + Na}^+^ species is observed at *m*/*z* 554.9998 (100%, **1**) and *m*/*z* 554.9990 (100%, **2**). Besides, {[Hg_2_(Pip)_4_] + Na}^+^ species are also identified at *m*/*z* 1087.0053 (77%, **1**) and *m*/*z* 1087.0057 (71%, **2**) (Supporting Information: Figure S1). On the other hand, for complexes **3**–**6**, the bidentate and tridentate dPy ligands are kept during ESI^+^ and a Pip linker is removed displaying values of *m*/*z* 523.0570 (100%, **3**), *m*/*z* 547.0576 (100%, **4**), *m*/*z* 600.0837 (100%, **5**), and *m*/*z* 566.0994 (100%, **6**) corresponding to {[Hg(Pip)(dPy)]}^+^ fragments (Supporting Information: Figure S2).

The thermal decomposition temperature was recorded for complexes **1**–**6**, showing decomposition between 171 and 197 °C (see the Experimental Section). In addition, simultaneous TG/DTA determination of **5** was carried out to confirm the presence of an occluded ethanol molecule in the powder sample (Supporting Information: Figure S3). The measurement was performed using 46.0 mg of sample. The complex starts to lose the occluded ethanol molecule at 90 °C (weight loss: exptl 4.9%, calcd 5.6%) until 140 °C, followed up by decomposition at about 200 °C by the loss of the terpy ligand (weight loss: exptl 29.1%, calcd 28.8%) and one Pip unit (weight loss: exptl 18.8%, calcd 20.3%).

In the FTIR-ATR spectra, the absence of bands between 2630 and 2518 cm^–1^ attributable to hydrogen bonded ν(O—H)_HPip_ and at 1667 cm^–1^ from ν(COOH) indicates that the HPip is deprotonated in the six complexes. The spectra of **1**–**6** display the characteristic carboxylate bands in the range 1626–1530 cm^–1^ for ν_as_(COO) and 1436–1415 cm^–1^ for ν_s_(COO) (Supporting Information: Figures S4–S9). The difference between these bands (Δ = ν_as_(COO) – ν_s_(COO)) reveals the coordination modes of the carboxylate linkers.^45^ Compounds **1**, **2**, and **4** display two different Δ values, 168 and 120, 179 and 114, and 188 and 101 cm^–1^, suggesting bidentate bridged and bidentate chelate (μ_2_-η^2^:η^1^) and chelate (μ_1_-η^2^) coordination modes of the carboxylate groups. Compounds **3**, **5**, and **6** have a Δ value of 112, 102, and 102 cm^–1^, indicating a bidentate chelate (μ_1_-η^2^) coordination mode. All of these values are in line with the data from the X-ray analysis. Moreover, in **6**, the peak corresponding to the ν(N—H) of the dpa is sharper compared to the free ligand, suggesting the *N*-amine coordination to the Hg(II) center. Further bands assigned to ν(C=C)/(C=N), δ(C=C)/(C=N), δ_ip_(C—H), and δ_oop_(C—H) from the aromatic rings have also been identified.^46^

The ^1^H NMR spectra of complexes **1**, **2**, **4**, and **5** were recorded in CDCl_3_ solvent, while those of **3** and **6** were recorded in dmso-*d*_6_ due to their lower solubility (Supporting Information: Figures S10–S15). The spectra of all of the complexes show three signals assigned to the aromatic protons of the Pip ligand between 7.77 and 6.71 ppm and to the −C*H*_2_– between 6.07 and 5.94 ppm. The bands of the corresponding dPy are located between 9.43 and 7.37 ppm, with the chemical shifts of *o*-*H*_py_ being consistent with the presence of *N*-coordinated ligands (8.91 (**1**), 8.76 (**2**), 8.88 (**3**), 9.43 (**4**), 9.23 (**5**), and 8.75 (**6**) ppm).^46^

The ^13^C{^1^H} NMR spectra of complexes **1**–**6** have been recorded in dmso-*d*_6_ solution as well as DEPT-135 experiments, which were performed only for those complexes required to ensure the correct assignation of the carbon atoms from the aromatic rings (Supporting Information: Figures S16–S21). The spectra of all of the complexes display the band assignable to the carbon atom of the carboxylate group at 169.20 (**1**), 168.97 (**2**), 169.01 (**3**), 169.16 (**4**), 169.38 (**5**), and 169.47 (**6**) ppm. The position of the pyridyl *o*-*C* atoms sorted in pairs of *N*-donors (148.89, 148.05 (**1**), and 150.26 (**2**)), *N*^*N*-donors (149.73 (**3**) and 150.37 (**4**)), and *N*^*N*^*N*-donors (150.65 (**5**) and 149.42 ppm (**6**)) enable us to better understand the electron donating ability of those ligands.^47^ These differences with the consequent upfield shift of those complexes containing 4-phpy (**2**), 1,10-phen (**4**), and terpy (**5**) are essentially caused either by resonance stabilization, inductive effects (−*I*), or both. In the case of 3-phpy (**1**) and 4-phpy (**2**), the better resonance stabilization, the more −*I* effect (with the p*K*_a_ value of the conjugate acid being 4.8 and 5.5,^48^ respectively), and the less steric hindrance confer to 4-Phpy a better donor character. Regarding the *N*^*N*-donors, the additional phenyl ring of 1,10-phen (**4**) with respect to 2,2′-bipy (**3**) results in a −*I* effect. Finally, the pyridyl substituents have a −*I* effect in terpy (**5**) compared to that of the −*CH*_2_– substituents in dpa (**6**), which is reflected in the upfield shift.

### Structural Description

#### Crystal and Extended Structure of Compounds **1** and **2**

**Figure 1:**
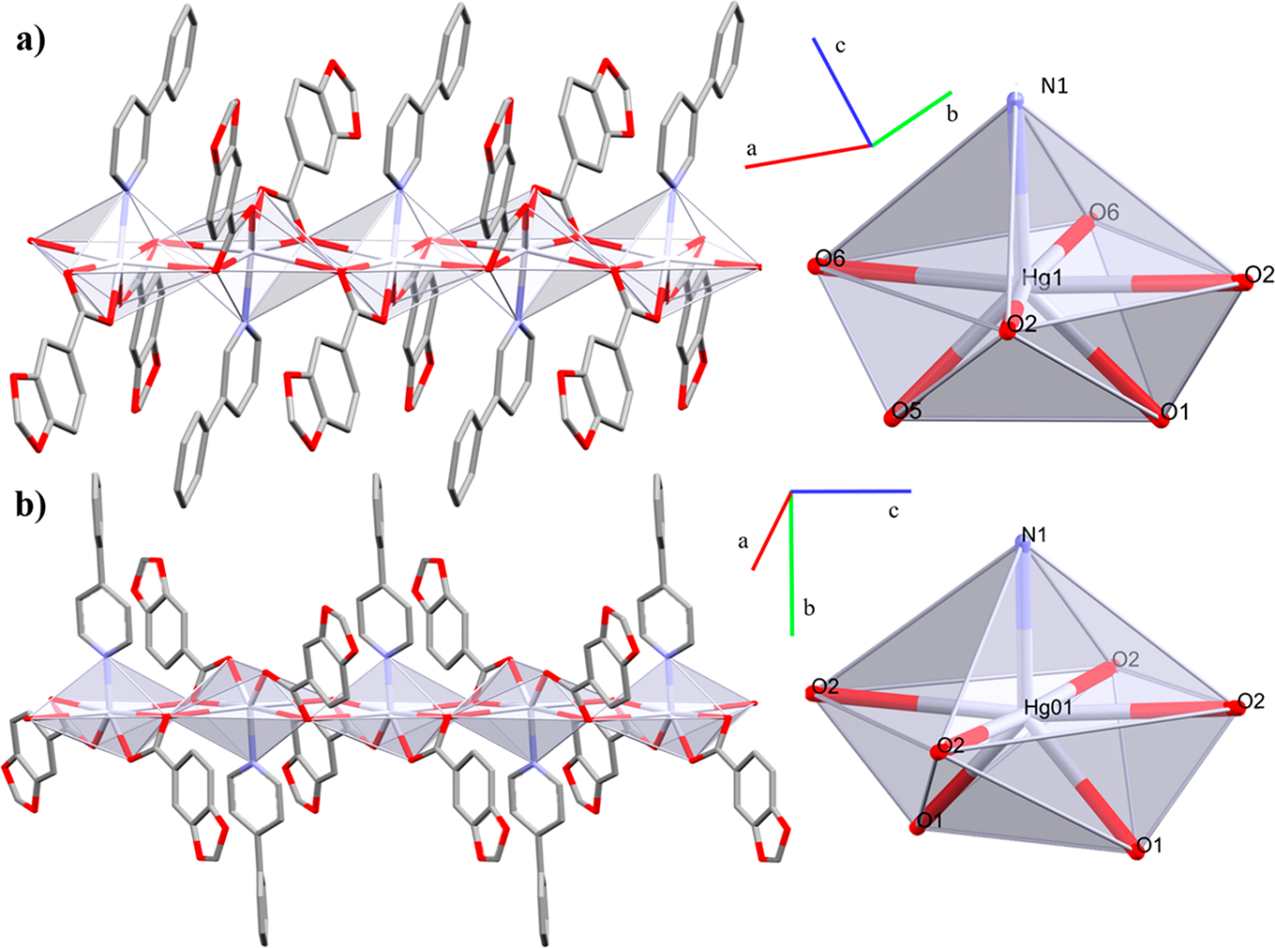
1D coordination polymers (a) **1** and (b) **2**. In detail, the coordination environment around the Hg(II) node. Hydrogen atoms are omitted for clarity.

They belong to the triclinic *P̅*1 (**1**) and monoclinic *C*2/*c* (**2**) space groups, with both being linear coordination polymers which expand through the *a* or *c* axis, respectively. All of the distances of less than the sum of the van der Waals radii have been considered to define the coordination sphere^49^ (Table 3). The Hg(II) centers have a [HgO_6_N] *core* with a distorted capped octahedral geometry composed of two Pip and either one 3-phpy (**1**) or 4-phpy (**2**) ligand. The Pip ligands define the formation of the edge-sharing polymeric array acting at the same time as bidentate bridged and chelate (μ_2_-η^2^:η^1^) linkers (Figure 1). Both have Hg–*N* bond lengths within the range of other reported coordination polymers.^23^

**Table 3:** Selected Bond Lengths (Å) and Bond Angles (deg) for **1** and **2**^a^

**1**			
Bond Lengths (Å)			
Hg(1)–N(1)	2.175(2)	Hg(1)–O(1)	2.222(2)
Hg(1)–O(5)	2.205(2)	Hg(1)–O(2)#1	2.733(2)
Hg(1)–O(6)	2.665(2)	Hg(1)–O(2)	2.7779(2)
Hg(1)–O(6)#2	2.855(2)		
Bond Angles (deg)			
N(1)–Hg(1)–O(5)	133.96(7)	O(5)–Hg(1)–O(1)	89.57(6)
N(1)–Hg(1)–O(1)	136.47(7)	O(1)–Hg(1)–O(2)	51.40(6)
O(1)–Hg(1)–O(2)#1	88.69(6)	O(1)–Hg(1)–O(6)#2	128.13(6)
O(1)–Hg(1)–O(6)	86.00(6)	O(2)–Hg(1)–O(5)	136.39(6)
O(2)–Hg(1)–O(2)#1	71.39(5)	O(2)–Hg(1)–O(6)#2	168.97(5)
O(2)–Hg(1)–O(6)	95.17(5)	O(2)*–Hg(1)–O(6)	165.88(5)
O(2)#1–Hg(1)–O(5)	91.77(6)	O(5)–Hg(1)–O(6)	101.25(6)
O(2)#1–Hg(1)–O(6)#2	119.19(5)	O(6)–Hg(1)–O(6)#2	74.02(5)
O(5)–Hg(1)–O(6)#2	50.38(6)	O(2)#1–Hg(1)–N(1)	90.02(6)
O(2)–Hg(1)–N(1)	87.24(6)	O(6)#2–Hg(1)–N(1)	89.57(6)
O(6)–Hg(1)–N(1)	84.88(6)		

**2**			
Bond Lengths (Å)			
Hg(01)–N(1)	2.204(7)	Hg(01)–O(2)#2	2.656(9)
Hg(01)–O(1)	2.258(6)	Hg(01)–O(2)	2.728(9)
Bond Angles (deg)			
N(1)–Hg(01)–O(1)	135.26(15)	O(1)–Hg(01)–O(1)#1	89.5(2)
N(1)–Hg(01)–O(2)#2	88.66(13)	O(1)–Hg(01)–O(2)#2	86.7(2)
O(1)–Hg(01)–O(2)#3	95.2(2)	O(2)#2–Hg(01)–O(2)#3	177.32(18)
O(1)–Hg(01)–O(2)	51.4(2)	O(2)–Hg(01)–N(1)	86.37(12)
O(2)–Hg(01)–O(1)#1	135.5(2)	O(2)–Hg(01)–O(2)#2	78.2(3)
O(2)–Hg(01)–O(2)#3	101.6(3)	O(2)–Hg(01)–O(2)#1	172.74(17)

a **1**: #1 1 – *x*, 1 – *y*, 1 – *z*; #2 2 – *x*, 1 – *y*, 1 – *z*. **2**: #1 *x* + 1, −*y*, −*z* + ^1^/_2_; #2 *x*, *y* + 1, *z* – ^1^/_2_; #3 −*x* + 1, −*y* + 1, −*z* + 1.

**Figure 2:**
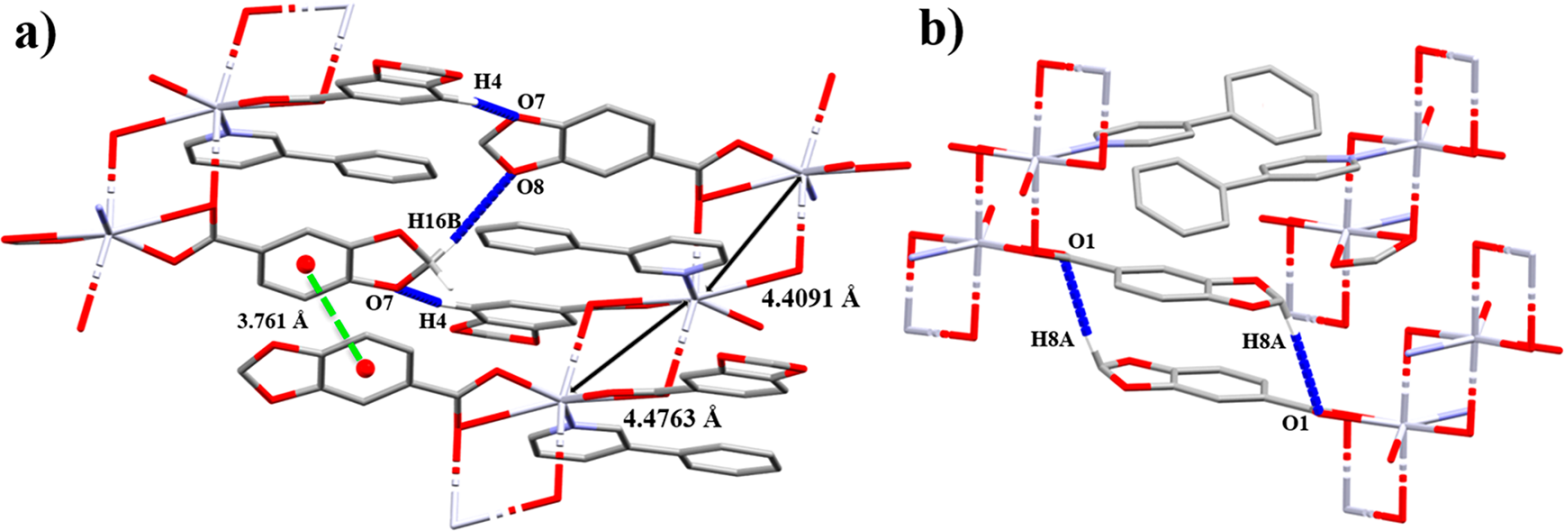
Supramolecular associations between polymeric chains of (a) **1** and (b) **2** through π···π and C–H···O interactions. Hg···Hg distance is indicated as a dark solid double arrow line. Hydrogen atoms and negligible aromatic rings are omitted for clarity.

With the 4-Phpy being less bulky than the 3-Phpy ligand, it facilitates a closer packing of the structure, which is reflected in the shortening of the Hg···Hg distance in **2** (4.1780(1) Å) with respect to **1** (4.4091(3) and 4.4763(3) Å). In **1**, the polymeric chains are stacked throughout the *b* axis by weak π···π interactions (Figure 2a) between the Pip rings (Cg(1)···Cg(1)′ 3.761 Å), while, in **2**, the Pip rings are too far (Cg(2)···Cg(2)′ 4.679 Å) as well as the phenyl groups of the 4-Phpy ligands (Cg(3)···Cg(3)′ 4.274 Å) to be considered as an effective π···π interaction (Figure 2b). Additional intermolecular C–H···O interactions support the final crystal packing (Table 4).

**Table 4:** Intermolecular Interactions of **1** and **2**^a^

**1**	H···A (Å)	D···A (Å)	D–H (Å)	>D–H···A (deg)
C16–H16A···O5	2.631	3.581(3)	0.990	160.97
C16–H16B···O8	2.565	3.263(3)	0.990	127.43
C8–H8B···O1	2.549	3.485(3)	0.990	157.67
C4–H4···O7	2.547	3.254(3)	0.951	131.29
C12–H12···O4	2.698	3.571(3)	0.950	153.03
C24–H24···O8	2.619	3.521(3)	0.950	158.82
C17–H17···O3	2.639	3.289(3)	0.950	126.02
π···π Interactions				
Cg(1)···Cg(1)	3.761 Å		71.14°	

**2**	H···A (Å)	D···A (Å)	D–H (Å)	>D–H···A (deg)
C8–H8A···O1	2.324	3.31(2)	0.99	173.22
π···π Interactions				
Cg(2)···Cg(2)	4.679 Å		50.36°	
Cg(3)···Cg(3)	4.274 Å		77.41°	

a **1**: Cg(1) = C10–C11–C12–C13–C14–C15. **2**: Cg(2) = C2–C3–C4–C5–C6–C7; Cg(3) = C12–C13–C14–C15–C3–C12.

#### Crystal and Extended Structure of Compound **3**

**Figure 3:**
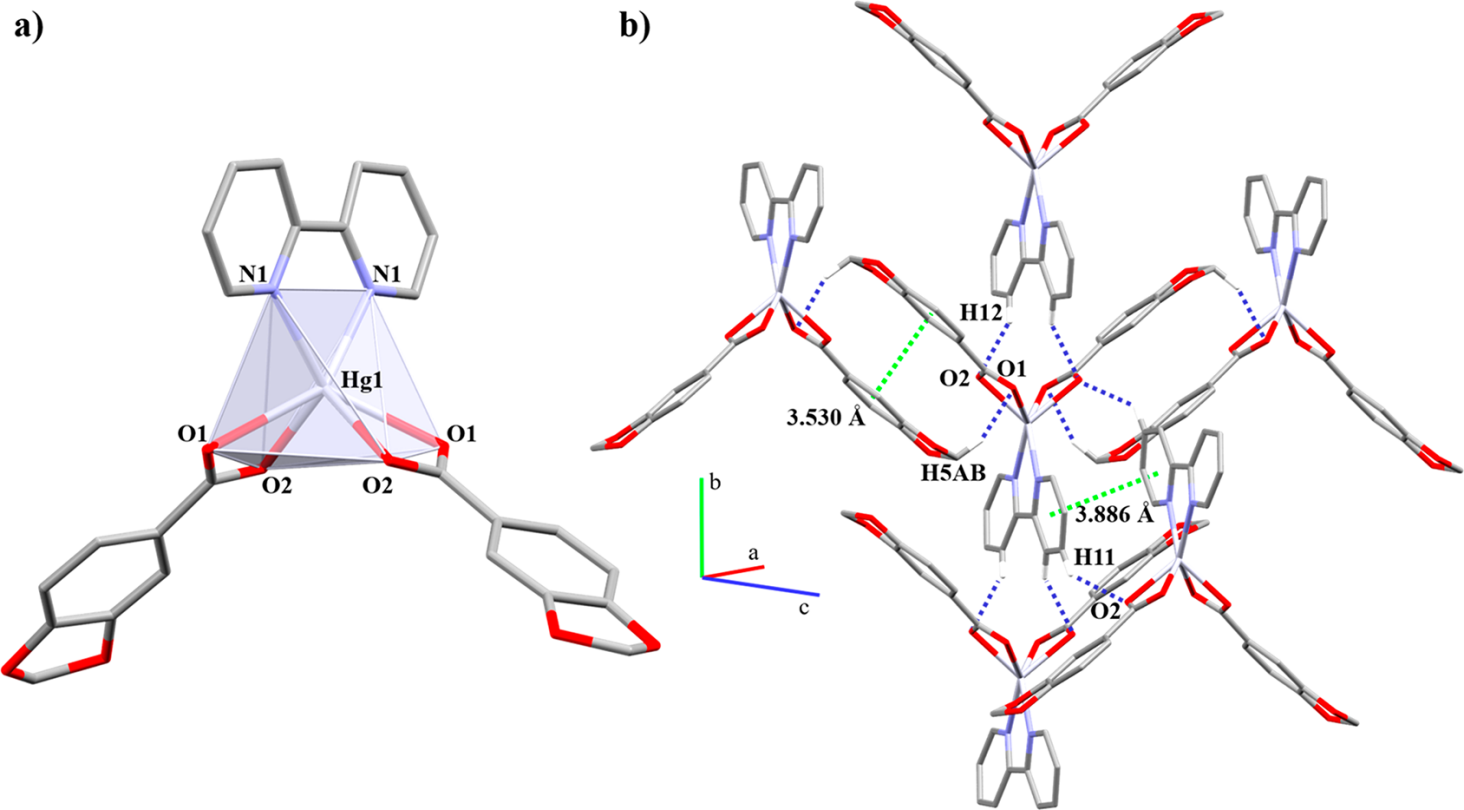
(a) Molecular structure highlighting the coordination environment of the Hg(II) ion and (b) C–H···O and π···π intermolecular interactions in **3**. Hydrogen atoms are omitted for clarity.

It belongs to the monoclinic *P*2/*c* space group. It is a Hg(II) monomer with a [HgO_4_N_2_] *core* (Table 5) and a distorted trigonal prismatic geometry composed of three bidentate chelate ligands (μ_1_-η^2^), two Pip, and one 2,2′-bipy (Figure 3a). The Hg–N bond lengths are slightly shorter compared to other similar Hg(II) complexes with 2,2′-bipy.^50,51^

**Table 5:** Selected Bond Lengths (Å) and Bond Angles (deg) for **3**^a^

bond lengths (Å)			
Hg(1)–O(1)#1	2.6281(16)	Hg(1)–N(1)	2.3096(17)
Hg(1)–O(2)	2.2527(16)		

bond angles (deg)			
O(2)#1–Hg(1)–O(2)	99.04(8)	O(2)–Hg(1)–O(1)#1	91.50(5)
O(2)#1–Hg(1)–N(1)	120.64(6)	N(1)–Hg(1)–O(1)#1	144.76(6)
O(2)–Hg(1)–N(1)	122.68(6)	N(1)–Hg(1)–O(1)	83.61(5)
N(1)–Hg(1)–N(1)#1	72.12(8)	O(1)#1–Hg(1)–O(1)	128.26(7)
O(2)#1–Hg(1)–O(1)#1	53.66(5)		

a **3**: #1 −*x*, *y*, −*z* + ^1^/_2_.

These monomeric units are held together forming chains along the *b* axis via double C–H···O interactions between the *m-*H of the 2,2′-bipy ligand and a carboxylate *O* atom of each Pip ligand (3.342(3) Å). These Pip ligands stack by π···π interactions (Cg(1)···Cg(1)′ 3.530 Å) and a double C–H···O association between one hydrogen atom of each dioxole group and the remaining carboxylate oxygen atom, which expands the crystal packing into 2D layers along the (1̅ 0 1) plane. Finally, a π···π interaction between the 2,2′-bipy (Cg(2)···Cg(2)′ 3.886 Å) and a C–H···O interaction between the *p-*H of the 2,2′-bipy and the remaining carboxylate *O* atom (3.288(3) Å) define the 3D supramolecular net (Figure 3b, Table 6).

**Table 6:** Intermolecular Interactions of **3**^a^

	H···A (Å)	D···A (Å)	D–H (Å)	>D–H···A (deg)	
C11–H11···O2	2.487	3.288(3)	0.950	141.95	
C12–H12···O2	2.465	3.342(3)	0.950	153.63	
C5–H5AB···O1	2.470	3.335(3)	0.990	145.62	
π···π Interactions					
Cg(1)···Cg(1)′	3.530 Å	92.13°	Cg(2)···Cg(2)′	3.886 Å	69.04°

a Cg(1) = Cg(1)′: C2–C3–C4–C6–C7–C8. Cg(2) = Cg(2)′: N1–C9–C10–C11–C12–C13.

#### Crystal and Extended Structure of Compound **4a**

**Figure 4:**
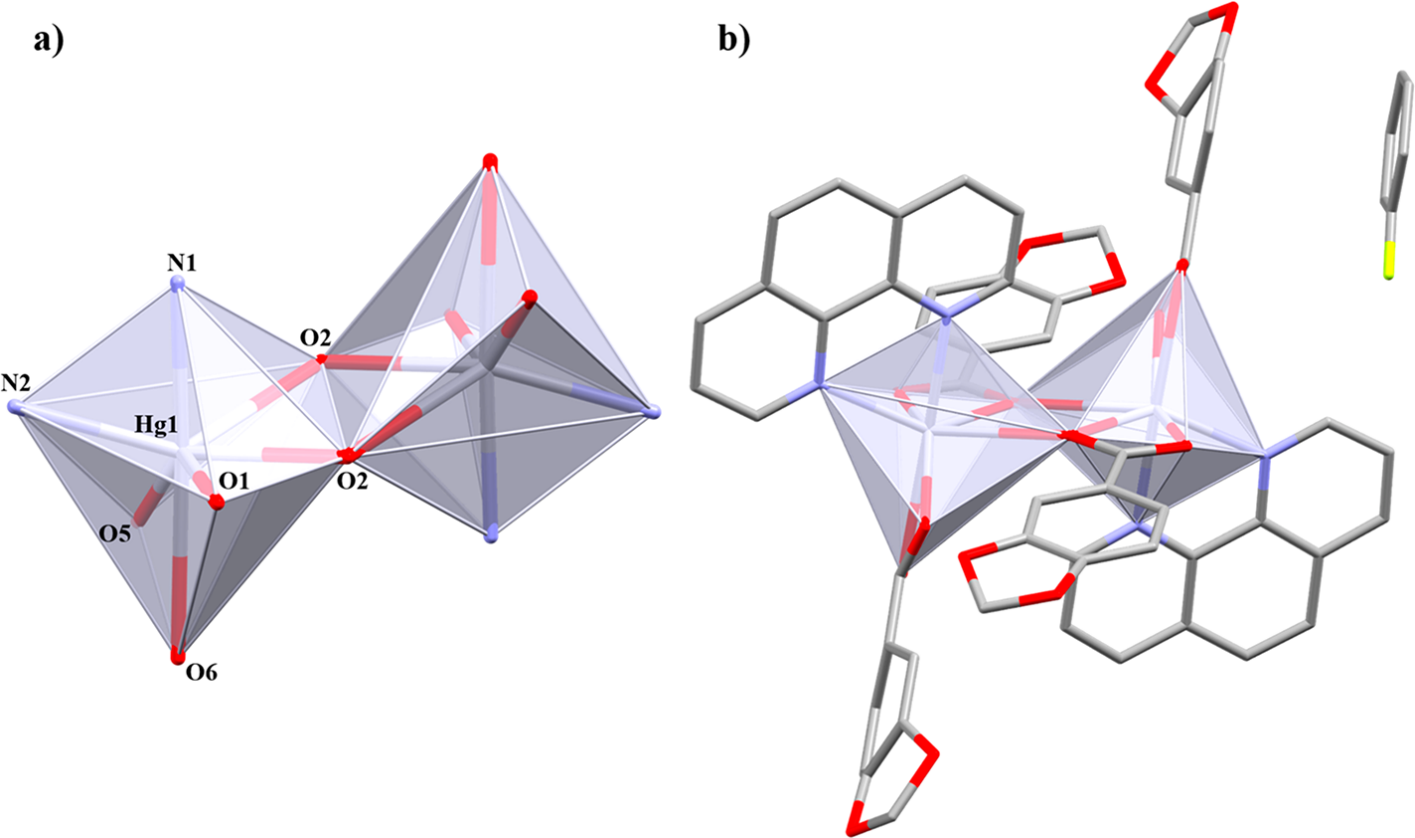
(a) Coordination environment of the Hg(II) ions and (b) molecular structure of **4**. Hydrogen atoms are omitted for clarity.

It belongs to the monoclinic *C*2/*c* space group. It has a dimeric array in which each Hg(II) has a [HgO_5_N_2_] *core* (Figure 4a), composed of two Pip ligands and one 1,10-phen linker in a distorted square antiprism geometry with bond angles ranging between 54.76(8) and 140.16(10)° (Table 7). One Pip unit and one 1,10-phen unit have a bidentate chelate (μ_1_-η^2^) coordination mode, occupying four coordination positions, while the remaining Pip linker joins the two Hg(II) centers through both bidentate chelate and bridged (μ_2_-η^2^:η^1^) coordination modes. In addition, there is an occluded fluorobenzene (Fbz) molecule (Figure 4b). The Hg–N bond lengths are in range with similar reported complexes.^50^

**Table 7:** Selected Bond Lengths (Å) and Bond Angles (deg) for **4a**^a^

bond lengths (Å)			
Hg(1)–O(1)	2.381(2)	Hg(1)–N(1)	2.344(3)
Hg(1)–O(2)	2.440(2)	Hg(1)–N(2)	2.420(3)
Hg(1)–O(2)	2.942(3)	Hg(1)–O(5)	2.176(2)
Hg(1)–O(6)	2.866(2)	Hg(1)–O(6)′	2.796(6)

bond angles (deg)			
O(5)–Hg(1)–N(1)	127.28(10)	O(6)′–Hg(1)–O(2)	116.64(17)
O(5)–Hg(1)–O(1)	137.79(9)	O(1)–Hg(1)–N(2)	90.58(8)
N(1)–Hg(1)–O(1)	93.94(9)	O(5)–Hg(1)–O(2)	126.41(9)
O(5)–Hg(1)–N(2)	94.79(10)	N(1)–Hg(1)–O(2)	87.35(9)
N(1)–Hg(1)–N(2)	70.65(9)	O(1)–Hg(1)–O(2)	54.76(8)
O(1)–Hg(1)–O(2)	127.04(8)	N(2)–Hg(1)–O(2)	138.04(8)
N(1)–Hg(1)–O(2)	76.28(8)	O(2)–Hg(1)–O(2)	76.68(7)
O(5)–Hg(1)–O(2)	77.72(8)	N(2)–Hg(1)–O(2)	131.20(8)
O(6)–Hg(1)–O(2)	103.66(17)	O(6)–Hg(1)–O(2)	95.95(14)
O(6)–Hg(1)–O(1)	88.85(14)	O(6)–Hg(1)–O(5)	49.64(15)
O(6)–Hg(1)–N(2)	107.30(17)	O(6)–Hg(1)–N(1)	176.52(14)
O(2)–Hg(1)–O(2)	72.68(7)	N(1)–Hg(1)–O(6)′	161.07(17)
N(2)–Hg(1)–O(6)′	90.61(18)	O(2)–Hg(1)–O(6)′	109.24(15)
O(6)′–Hg(1)–O(5)	49.50(16)	O(1)–Hg(1)–O(6)′	88.70(15)

a O(6)′ defines a disordered carboxylate oxygen atom.

**Figure 5:**
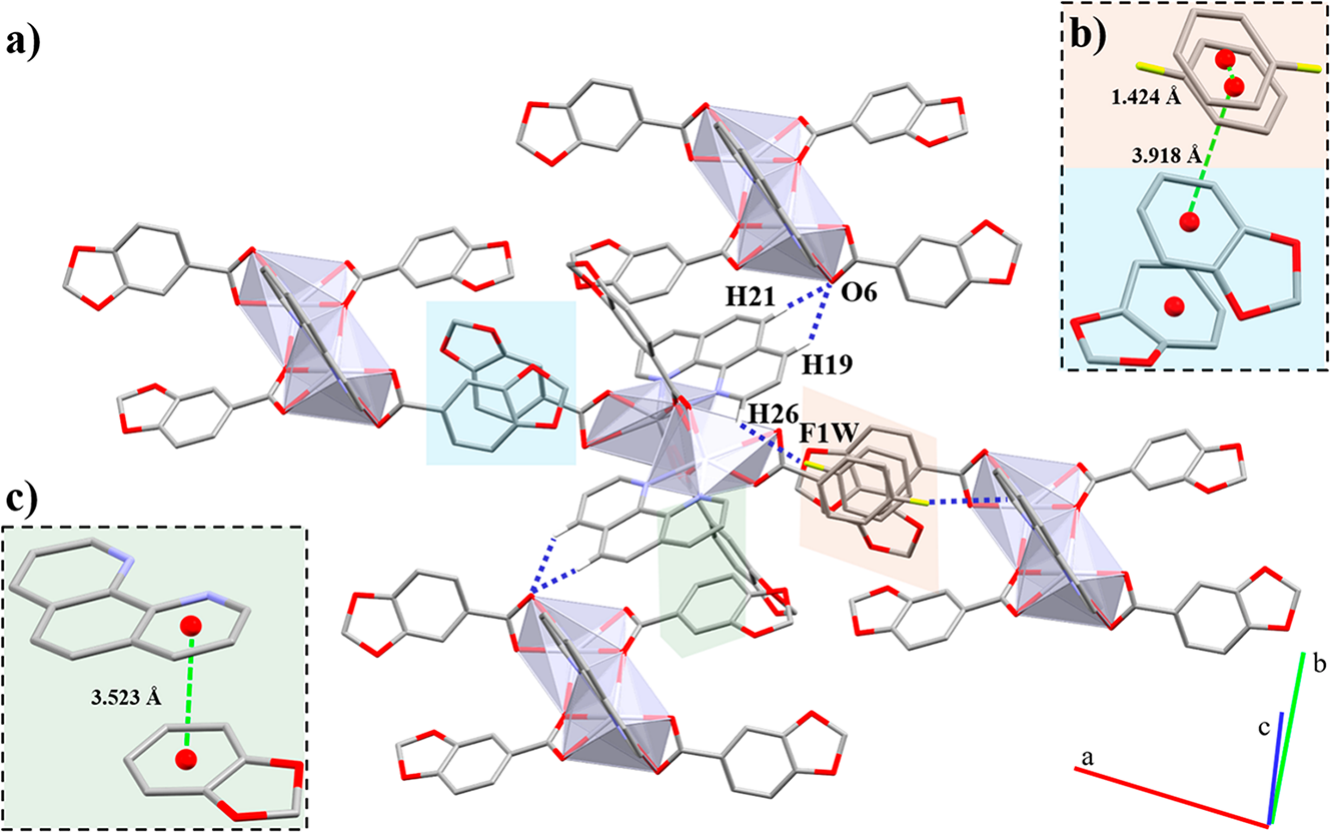
Representation of the double C–H···O interaction in **4** and highlighting of the π···π between Pip···Pip (a, light blue), Fbz and Pip (b, orange), and 1,10-phen···Pip (c, green) aromatic rings. Hydrogen atoms not involved in the intermolecular interactions are omitted for clarity.

The 1,10-phen ligands act as hydrogen bond donors through double and single C–H···O interactions with the Pip linkers (Table 8). The double interaction occurs *via* both the *p*-H and the contiguous H from the phenyl ring with the same *O* atom from the carboxylate (Figure 5a) and arranges the dimeric units into 2D layers along the *bc* plane.

**Table 8:** Intermolecular Interactions of **4a**^a^

	H···A (Å)	D···A (Å)	D–H (Å)		>D–H···A (deg)
C21–H21···O6	2.495	3.278(7)	0.950		139.75
C19–H19···O6	2.445	3.233(7)	0.950		140.29
C25–H25···O8	2.47	3.34(2)	0.950		152.11
C26–H26···F1W	2.398	3.291(7)	0.950		156.48
π···π Interactions					
Cg(1)···Cg(1)′	4.089 Å	95.30°	Cg(3)···Cg(4)	3.523 Å	94.44°
Cg(1)···Cg(2)	3.918 Å	104.68°	Cg(5)···Cg(6)	3.603 Å	96.21°
Cg(2)···Cg(2)′	1.424 Å	30.62°			

a Cg(1) = Cg(1)′: C11–C12–C14–C15–C16–C17. Cg(2) = Cg(2)′: C1W–C2W–C3W–C4W–C5W–C6W. Cg(3): N2–C23–C24–C25–C26–C27. Cg(4): C2–C3–C4–C6–C7–C8. Cg(5): N2–C23–C24–C25–C26–C27. Cg(6): C20–C21–C22–C23–C27–C28.

**Figure 6:**
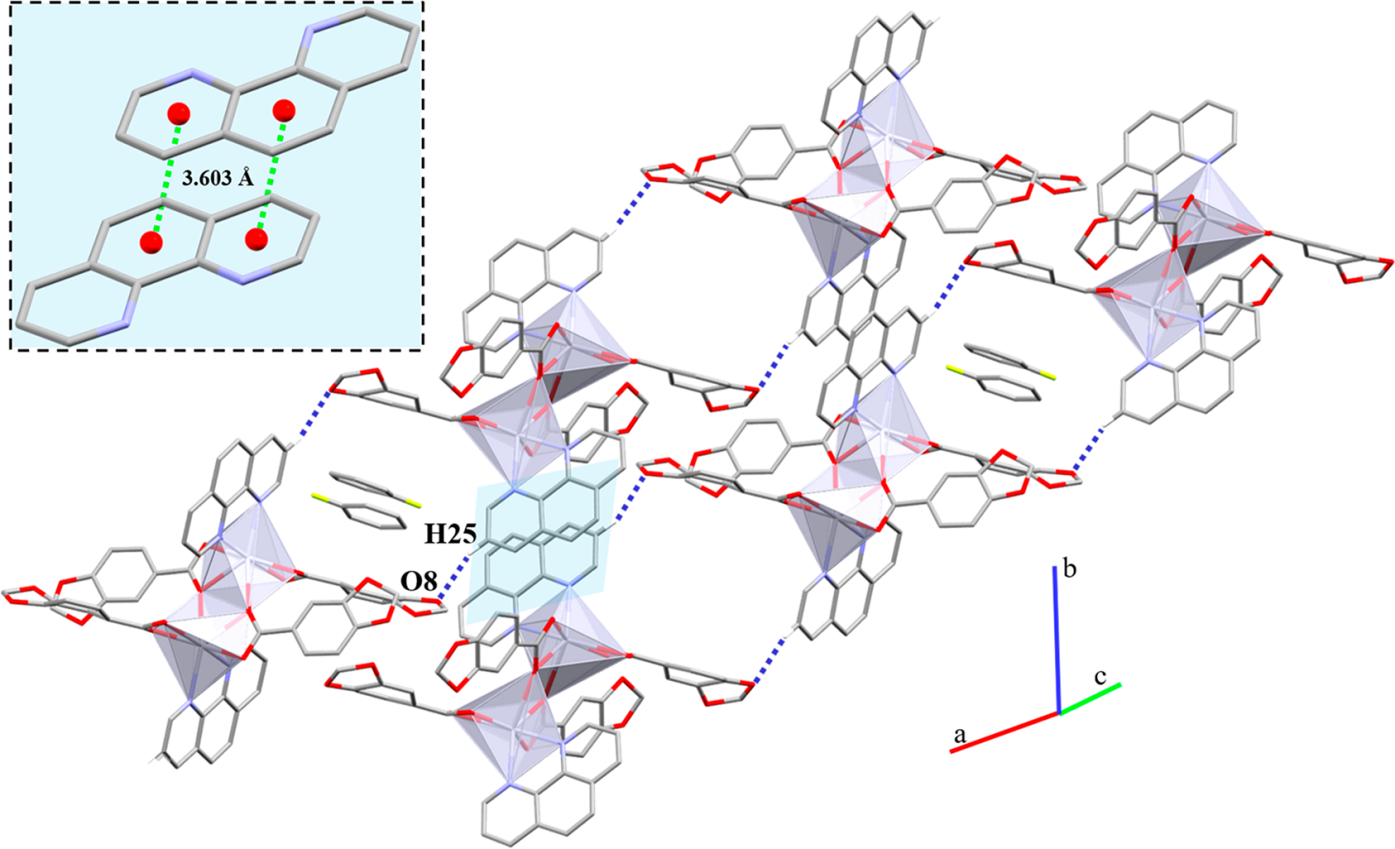
Representation of the C–H···O interaction in **4** and the π···π interaction in pairs between 1,10-phen rings highlighted in light blue. Hydrogen atoms not involved in the intermolecular interactions are omitted for clarity.

The occluded Fbz molecules are π···π stacked (Cg(2)···Cg(2)′, 1.424 Å) in pairs and joined to the dimeric units by π···π with the Pip rings (Cg(1)···Cg(2), 3.918 Å) and a C–H···F interaction (Table 8, Figure 5b). Besides, there is an additional π···π (1,10-phen···Pip (Cg(3)···Cg(4), 3.523 Å)), cooperating in the formation of these layers (Figure 5c). The remaining *m*-H from the opposite pyridyl ring interacts with one dioxole *O* atom along the [101̅] direction, supported by reciprocal π···π interactions between pairs of 1,10-phen aromatic rings (Cg(5)···Cg(6), 3.603 Å) which drive the formation of the 3D net (Figure 6).

#### Crystal and Extended Structure of Compound **5**

**Figure 7:**
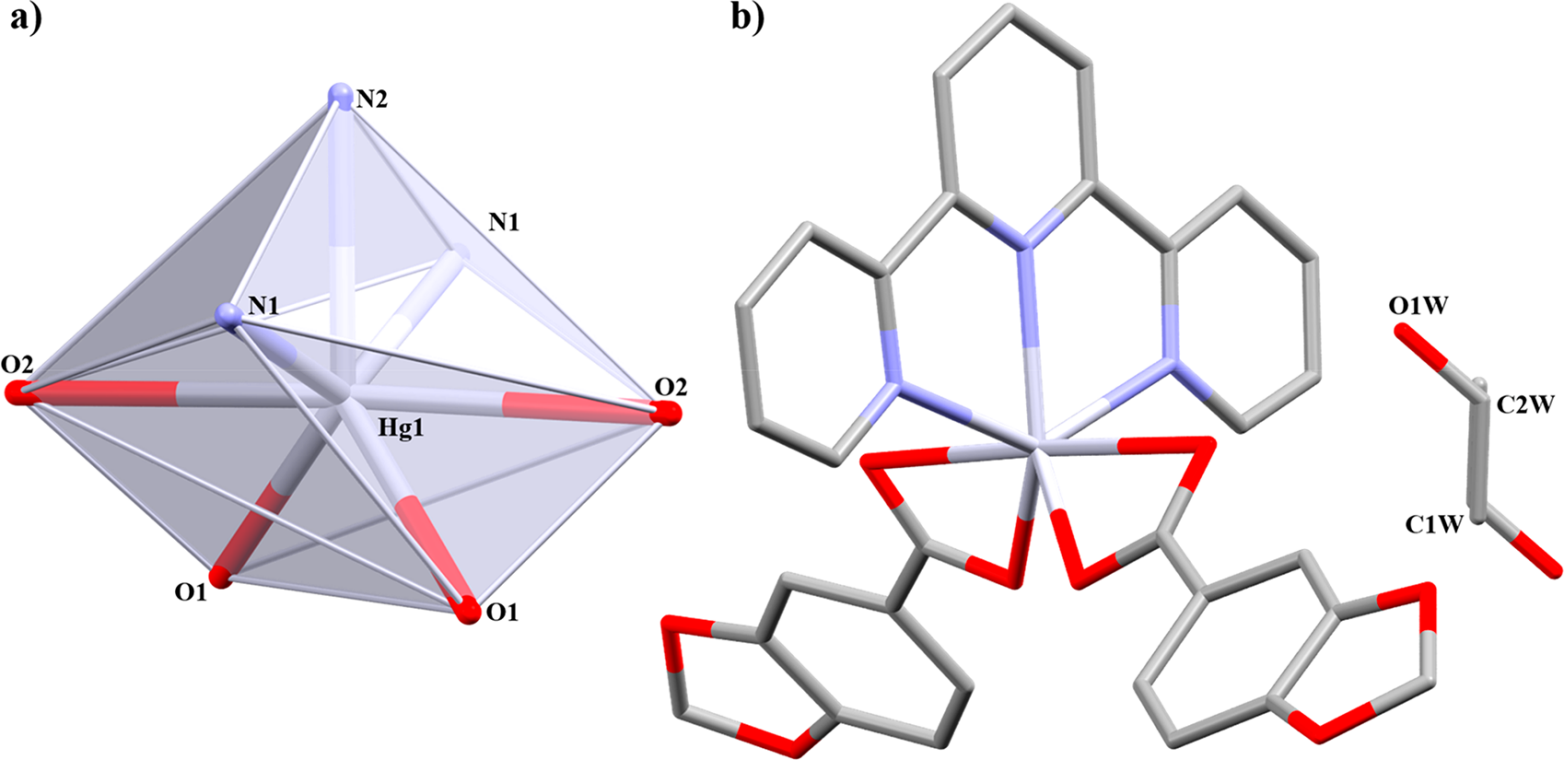
(a) Coordination environment of the Hg(II) ions and (b) molecular structure of **5**. Hydrogen atoms are omitted for clarity.

It belongs to the monoclinic *C*2/*c* space group. It is a Hg(II) monomer with a [HgO_4_N_3_] *core* (Figure 7a) and a distorted capped octahedral geometry composed of two asymmetrically bidentate chelate Pip (μ_1_-η^2^) and a tridentate chelate terpy (μ_1_-η^3^) ligand (Figure 7b) with Hg–O and Hg–N bond lengths within the range of similar complexes^52,53^ (Table 9).

**Table 9:** Selected Bond Lengths (Å) and Bond Angles (deg) for **5**^a^

bond lengths (Å)			
Hg(1)–O(1)#1	2.2819(11)	Hg(1)–N(1)#1	2.3995(12)
Hg(1)–O(2)	2.7229(15)	Hg(1)–N(2)	2.3620(16)

bond angles (deg)			
O(1)–Hg(1)–O(1)#1	93.40(4)	O(1)–Hg(1)–O(2)	51.83(4)
O(1)–Hg(1)–N(2)	133.30(3)	O(1)–Hg(1)–N(1)#1	84.34(4)
N(2)–Hg(1)–N(1)	68.89(3)	O(1)–Hg(1)–N(1)	126.34(4)
O(1)#1–Hg(1)–O(2)	126.00(4)	N(1)#1–Hg(1)–N(1)	137.79(4)
N(1)#1–Hg(1)–O(2)	93.68(4)	O(2)#1–Hg(1)–N(1)#1	87.23(4)
N(2)–Hg(1)–O(2)	91.26(2)	O(2)–Hg(1)–O(2)#1	177.47(3)

a **5**: #1 1 – *x*, *y*, 3/2 – *z*.

**Figure 8:**
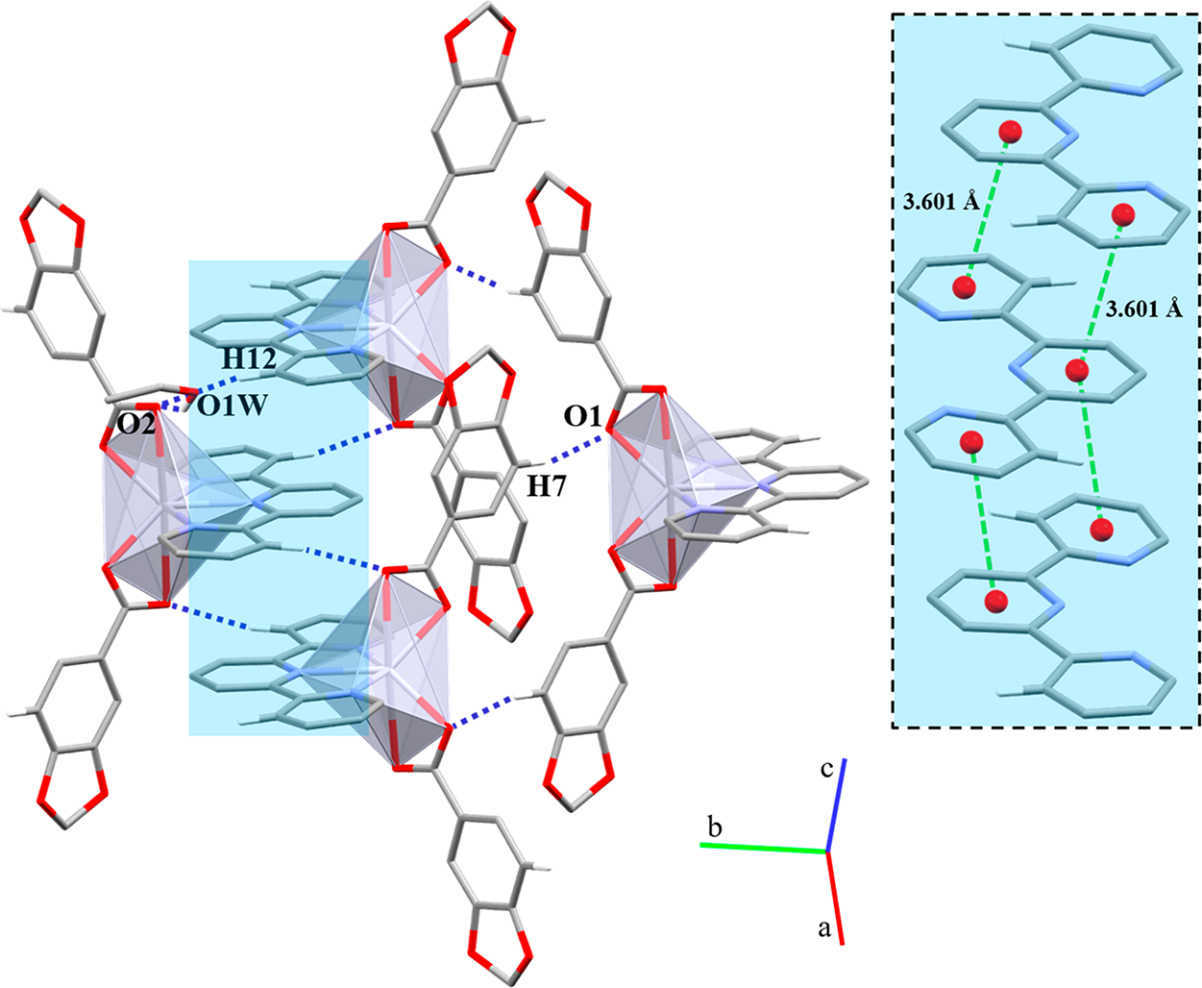
Representation of the C–H···O interactions in **5** and the π···π interaction between terpy rings highlighted in light blue. Hydrogen atoms not involved in the intermolecular interactions are omitted for clarity.

The terpy ligands arrange the monomeric units into 1D chains through reciprocal π···π stacking (Cg(1)···Cg(2), 3.601 Å) along the [1̅01] direction. These chains are joined through the *b* axis by two different C–H···O interactions (terpy···terpy and terpy···Pip) (Table 10). Both *m*-H of the lateral pyridyl rings associate with one carboxylate *O* atom, while the Pip ligands interact between them via the *m*-H and the remaining carboxylate *O* atom. In addition, the EtOH molecule is hydrogen bonded to the same carboxylate *O* atom as the *m*-H (Figure 8).

**Table 10:** Intermolecular Interactions of **5**^a^

	H···A (Å)	D···A (Å)	D–H (Å)	>D–H···A (deg)
O1W–H10···O2	2.025	2.775(3)	0.840	148.14
C12–H12···O2	2.348	3.286(2)	0.950	169.36
C7–H7···O1	2.369	3.293(2)	0.950	163.99
π···π Interactions				
Cg(1)···Cg(2)	3.601 Å		74.99°	

a Cg(1): N1–C9–C10–C11–C12–C13. Cg(2): N2–C14–C15–C16–C15–C14.

#### Crystal and Extended Structure of Compound **6a**

**Figure 9:**
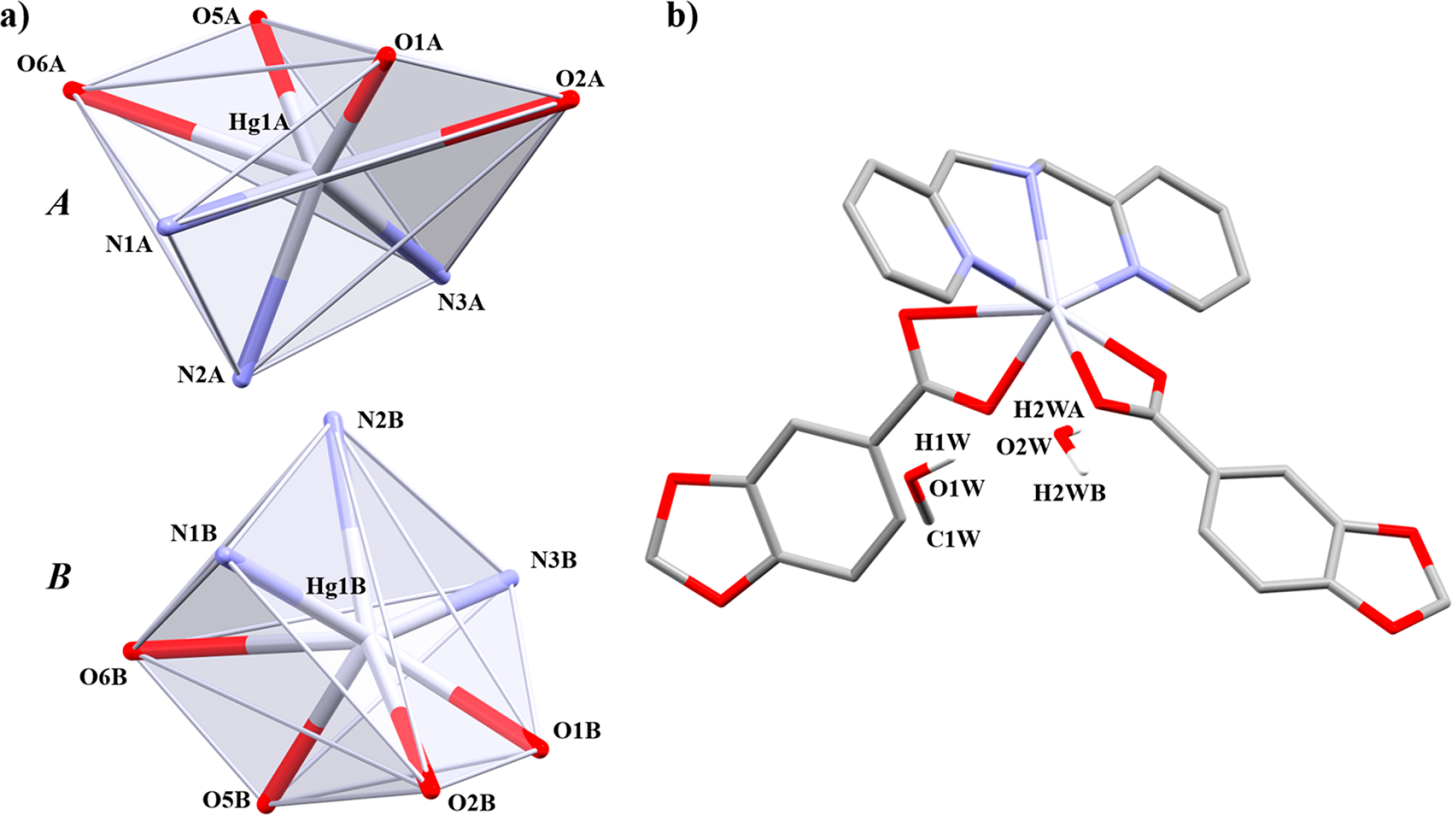
(a) Coordination environment of the Hg(II) ions on the two crystallographically independent monomers and (b) molecular structure of **6a**. Hydrogen atoms except those from the −OH and H_2_O molecules are omitted for clarity.

It belongs to the triclinic *P̅*1 space group, having two crystallographically independent monomeric units (*A* and *B*) and sharing the same [HgO_4_N_3_] *core* (Table 11), both presenting distorted capped octahedral geometries (Figure 9a). The monomeric units are composed of two bidentate chelate Pip (μ_1_-η^2^) linkers and one *mer*-tridentate chelate dpa (μ_1_-η^3^) ligand.^54^ The twisting of the pyridyl rings (angle between rings, 22.11°) is favored by the free rotation of the −*CH*_2_– (Figure 9b).

**Table 11:** Selected Bond Lengths (Å) and Bond Angles (deg) for **6a**

bond lengths (Å)			
**A**			
Hg(1A)–O(1A)	2.2216(11)	Hg(1A)–N(1A)	2.4579(13)
Hg(1A)–O(5A)	2.3068(11)	Hg(1A)–O(6A)	2.9420(10)
Hg(1A)–N(3A)	2.3597(12)	Hg(1A)–O(2A)	2.9530(10)
Hg(1A)–N(2A)	2.3620(13)		
**B**			
Hg(1B)–O(1B)	2.2483(10)	Hg(1B)–N(3B)	2.4243(12)
Hg(1B)–O(5B)	2.2622(11)	Hg(1B)–O(2B)	3.0310(10)
Hg(1B)–N(1B)	2.3232(12)	Hg(1B)–O(6B)	2.9070(10)
Hg(1B)–N(2B)	2.4110(12)		

bond angles (deg)			
**A**			
O(6A)–Hg(1A)–O(5A)	48.40(4)	O(1A)–Hg(1A)–N(1A)	80.33(4)
O(6A)–Hg(1A)–N(1A)	70.93(4)	O(1A)–Hg(1A)–N(2A)	139.75(4)
O(6A)–Hg(1A)–N(2A)	86.12(4)	O(1A)–Hg(1A)–N(3A)	125.63(4)
O(6A)–Hg(1A)–N(3A)	116.98(4)	O(1A)–Hg(1A)–O(2A)	48.51(4)
O(6A)–Hg(1A)–O(1A)	109.80(4)	O(2A)–Hg(1A)–N(1A)	120.15(4)
O(6A)–Hg(1A)–O(2A)	145.44(4)	O(2A)–Hg(1A)–N(2A)	128.21(4)
O(5A)–Hg(1A)–N(1A)	114.71(4)	O(2A)–Hg(1A)–N(3A)	77.12(4)
O(5A)–Hg(1A)–N(2A)	119.31(4)	N(1A)–Hg(1A)–N(2A)	70.14(4)
O(5A)–Hg(1A)–N(3A)	93.72(4)	N(1A)–Hg(1A)–N(3A)	139.70(4)
O(5A)–Hg(1A)–O(1A)	97.42(4)	N(2A)–Hg(1A)–N(3A)	71.14(4)
O(5A)–Hg(1A)–O(2A)	102.30(4)		
**B**			
O(6B)–Hg(1B)–O(5B)	48.94(4)	O(1B)–Hg(1B)–N(1B)	122.81(4)
O(6B)–Hg(1B)–N(1B)	77.04(4)	O(1B)–Hg(1B)–N(2B)	133.64(4)
O(6B)–Hg(1B)–N(2B)	77.62(4)	O(1B)–Hg(1B)–N(3B)	81.33(4)
O(6B)–Hg(1B)–N(3B)	99.83(4)	O(1B)–Hg(1B)–O(2B)	46.94(4)
O(6B)–Hg(1B)–O(1B)	144.87(4)	O(2B)–Hg(1B)–N(1B)	78.97(4)
O(6B)–Hg(1B)–O(2B)	125.46(4)	O(2B)–Hg(1B)–N(2B)	137.24(4)
O(5B)–Hg(1B)–N(1B)	104.28(4)	O(2B)–Hg(1B)–N(3B)	127.79(4)
O(5B)–Hg(1B)–N(2B)	124.46(4)	N(1B)–Hg(1B)–N(2B)	71.84(4)
O(5B)–Hg(1B)–N(3B)	101.08(4)	N(1B)–Hg(1B)–N(3B)	142.06(4)
O(5B)–Hg(1B)–O(1B)	96.14(4)	N(2B)–Hg(1B)–N(3B)	70.60(4)
O(5B)–Hg(1B)–O(2B)	93.31(4)		

Both monomers are associated by reciprocal N–H···O interactions between the amino group and a carboxylate *O* atom. *B* monomers are stacked in pairs between themselves through reciprocal π···π interactions promoted by the dpa ligands, while the corresponding aromatic rings in *A* are too far for an effective π···π stacking (*B*–*B* = Cg(3)···Cg(4), 3.751 Å; *A*–*A* = Cg(1)···Cg(2), 4.203 Å).

**Figure 10:**
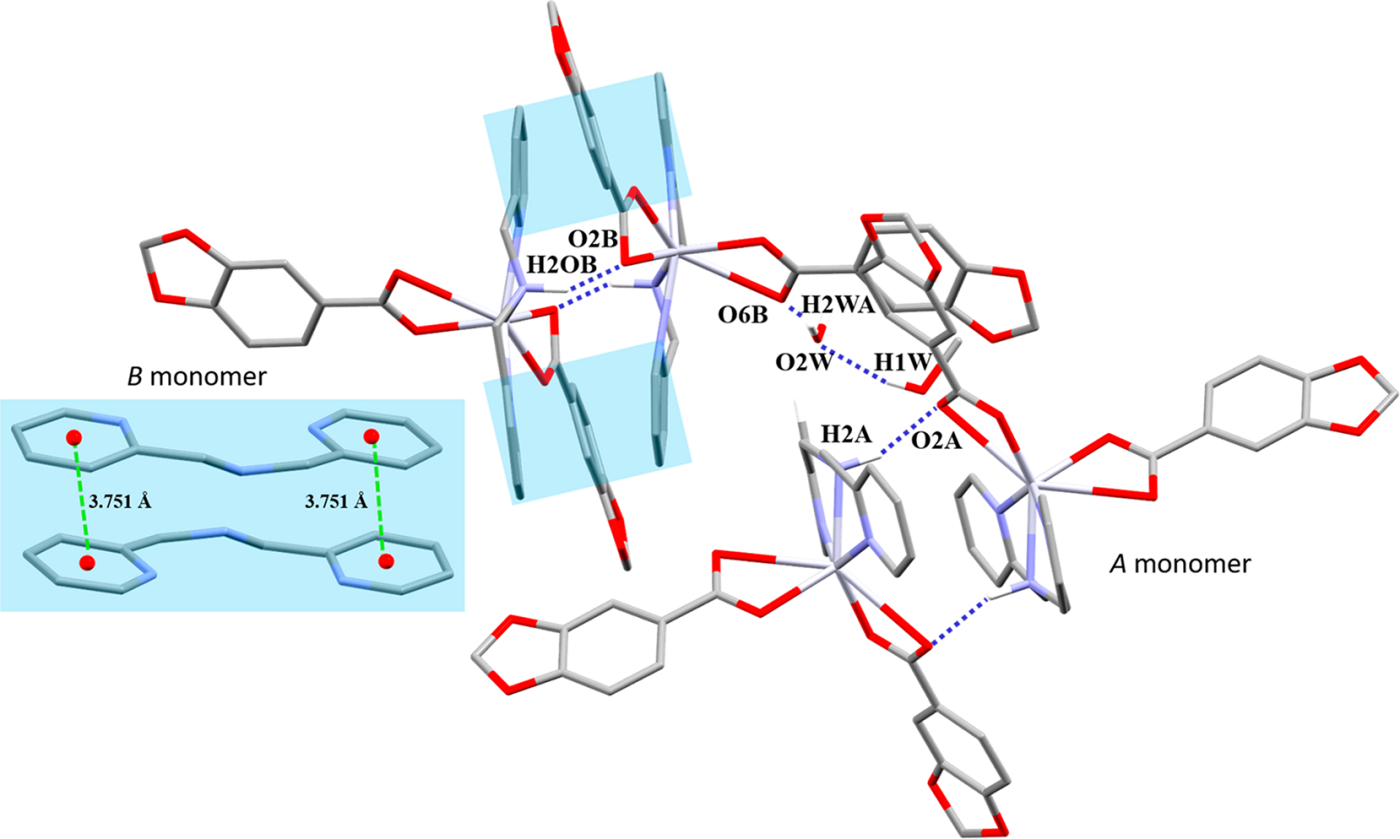
Representation of the N–H···O interactions in **6a** and the π···π interaction between dpa rings highlighted in light blue and light green. Hydrogen atoms not involved in the intermolecular interactions are omitted for clarity.

**Figure 11:**
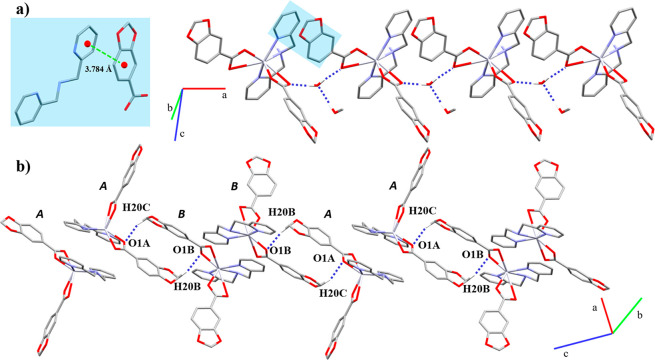
Representation of (a) O–H···O and the π···π interaction between dpa rings in **6a** highlighted in light blue. (b) C–H···O interactions between Pip ligands. Hydrogen atoms not involved in the intermolecular interactions are omitted for clarity.

The occluded water molecule acts simultaneously as an acceptor with a MeOH molecule and as a donor with the carboxylate *O* atom through O–H···O interactions (Figure 10). Both combined with a π···π stacking (Cg(4)···Cg(5), 3.784 Å) between one pyridyl ring, and the aromatic ring of a carboxylate linker (Figure 11a) associate *B* monomers along the *a* axis. *A* and *B* monomers are joined through a C–H···O interaction between the −CH_2_– (*A*) and a carboxylate O atom (*B*) (Table 12, Figure 11b).

**Table 12:** Intermolecular Interactions of **6a**^a^

	H···A (Å)	D···A (Å)	D–H (Å)	>D–H···A (deg)
N2A–H2A···O2A	1.948	2.870(2)	1.000	152.02
N2B–H2B···O2B	2.333	3.126(2)	1.000	135.56
O2W–H2WA···O6B	1.92(2)	2.721(2)	0.81(2)	170.47
O2W–H2WB···O2B	1.92(2)	2.725(2)	0.81(2)	170.03
O1W–H1W···O2W	1.979	2.797(2)	0.840	164.40
C20–H20C···O1A	2.250	3.200(2)	0.990	160.41
π···π Interactions				
*A*				
Cg(1)···Cg(2)	4.203 Å		69.10°	
*B*				
Cg(3)···Cg(4)	3.751 Å		73.54°	
Cg(4)···Cg(5)	3.784 Å		65.44°	

a *A* = Cg(1): N1A–C1A–C2A–C3A–C4A–C5A; Cg(2): N3A–C8A–C9A–C10A–C11A–C12A. *B* = Cg(3): N1B–C1B–C2B–C3B–C4B–C5B; Cg(4): N3B–C8B–C9B–C10B–C11B–C12B; Cg(5): C14B–C15B–C16B–C17B–C18B–C19B.

#### Structural Comparison and Steric Effects

Bidentate *N*^*N′*-donor (2,2′-bipy and 1,10-phen) and tridentate *N*^*N*^*N*-donor (terpy and dpa) ligands formed five-membered rings after chelation with the Hg(II) centers. Since Hg(II) has a big van der Waals radius (1.7–2.0 Å)^55^ allowing high coordination numbers (6 and 7), the steric effects of dPy ligands guide the formation of complexes with diverse structural arrangements, varying from monomers to coordination polymers.

Bite angle (∠*N*–M–*N*) measurements were an initial tool to evaluate steric effects of chelate ligands, but as only the donor atoms are considered, the bulkiness of the ligand tends to be underestimated. Further steric parameters, such as the *inter alia tolman angle*,^56^*exact ligand cone angle*,^57^ and *solid angle*,^58^ better define free rotation ligands. For this reason, we have defined a straightforward angle measurement (*outer atoms angle*) that was done considering the planarity of the linkers and, therefore, assuming that the steric effects are predominantly generated by two hydrogen outer atoms placed at the sides of the dPy ligands, especially remarkable for those having a chelate effect and free rotation restrictions. All of the angles formed by two outer hydrogen atoms of each ligand and its metal center have been examined from the crystallographic data using Mercury software version 4.3.1,^30−32^ and only the bigger ones are listed in Table 13.

**Table 13:** Structural Parameters Regarding the Steric Effects of the Ligands

sample	chelate angle (Pip)	bite angle (dPy)	outer atom angle^a^
**1**	50.38; 51.40		92.40°
**2**	51.41		78.84°
**3**	53.66	72.12	146.25°
**4a**	54.76; 49.6	70.65	141.28°
**5**	51.83	68.89; 137.78	210.68°
**6a**	48.94; 46.94	70.60; 71.85; 142.06	221.68°

a The angles are calculated between the outer atoms of the dPy ligand and the Hg(II) center using Mercury software.^30−32^

This angle permitted a reasonable comparison of the steric effects of the dPy ligands into the final complexes and explains the different nuclearity of the complexes containing the monodentate linkers 3-phpy and 4-phpy having smaller angles (50° less than chelate). Besides, the 1,10-phen linker being almost 5° less bulky than 2,2′-bipy promotes the formation of a dimeric structure instead of the monomeric array promoted by the ligands having bigger angles (2,2′-bipy, terpy, and dpa).

### Photophysical Properties

#### UV–vis Spectroscopy

All of the samples were dissolved in MeOH, and their UV–vis spectra were recorded at 298 K. Since π···π interactions are important noncovalent intermolecular forces and contribute to self-assembly processes,^59,60^ additive UV–vis measurements were performed within a concentration range from ∼1 × 10^–9^ to 1 × 10^–4^ M to ensure the nonaggregation of the ligands and complexes **1**–**6** at the selected concentration for the photoluminescence experiments and avoid the aggregation-caused emission quenching (ACQ) effect^61^ (Supporting Information: Figures S22 and S23). Complexes start to aggregate at 7.67 × 10^–6^ M (**1**), 8.41 × 10^–6^ M (**2**), 1.89 × 10^–7^ M (**4**), 4.44 × 10^–7^ M (**5**), and 1.63 × 10^–5^ M (**6**). Within this tendency, complex **3** which presented weaker interactions seems to avoid aggregation effects at higher concentrations. Those which exhibited stronger interactions in the solid state identified in the structural analysis tend to easily aggregate in solution and promote a bathochromic shift emphasized at higher wavelength absorption peaks.^62^ Especially, complexes **5** and **6** exhibited bathochromic aggregation effects reflected by the shift of absorption bands at 333 and 302 nm, respectively. Likewise, after aggregation of **1**, a band at 293 nm emerged. Hereafter, the UV–vis measurements of complexes **1**–**6** have been done using a concentration around ∼1.00 × 10^–7^ M (Supporting Information: Figure S24). The UV–vis spectra of the ligands and l-tyrosine (l-tyr) have been recorded using the same concentration (Supporting Information: Table S1). The absorption maxima (λ_max_) of the complexes and the ligands have been identified and their molar absorptivity values (ε) calculated (Table 14).

**Table 14:** UV–vis and Fluorescence Data of Complexes **1**–**6**^a^

sample	λ_max-Abs_ (log(ε))	λ_ex_	λ_max-em_	ϕ_S_
**1**	212 (4.52); 258 (4.43); 278 (4.13); 293 (4.19)^b^	230	346	0.03
**2**	205 (4.78); 259 (4.50); 293 (4.22)	250	348	0.01
**3**	203 (4.69); 215 (4.66); 244 (4.18); 260 (4.25); 287 (4.29)	233	340; 353	0.97
**4**	206 (4.78); 216 (5.03);^b^ 266 (5.05); 294 (4.93); 326 (3.68)^b^	230	345	0.05
**5**	205 (4.85); 253 (4.28); 282 (4.30); 298 (4.15);^b^ 322 (4.17)^b^	269	356	0.37
**6**	202 (4.17); 238 (4.03); 281 (3.88); 307 (3.99)^b^	250	316	0.05

a All of the wavelengths are given in nm. ε values are given in M^–1^·cm^–1^. λ_max-Abs_ = maximum of absorption. λ_ex_ = excitation wavelength; λ_max-em_ = maximum of emission; ϕ_S_ = quantum yield.

b Bands arising from aggregation effects.

#### Photoluminescence

**Figure 12:**
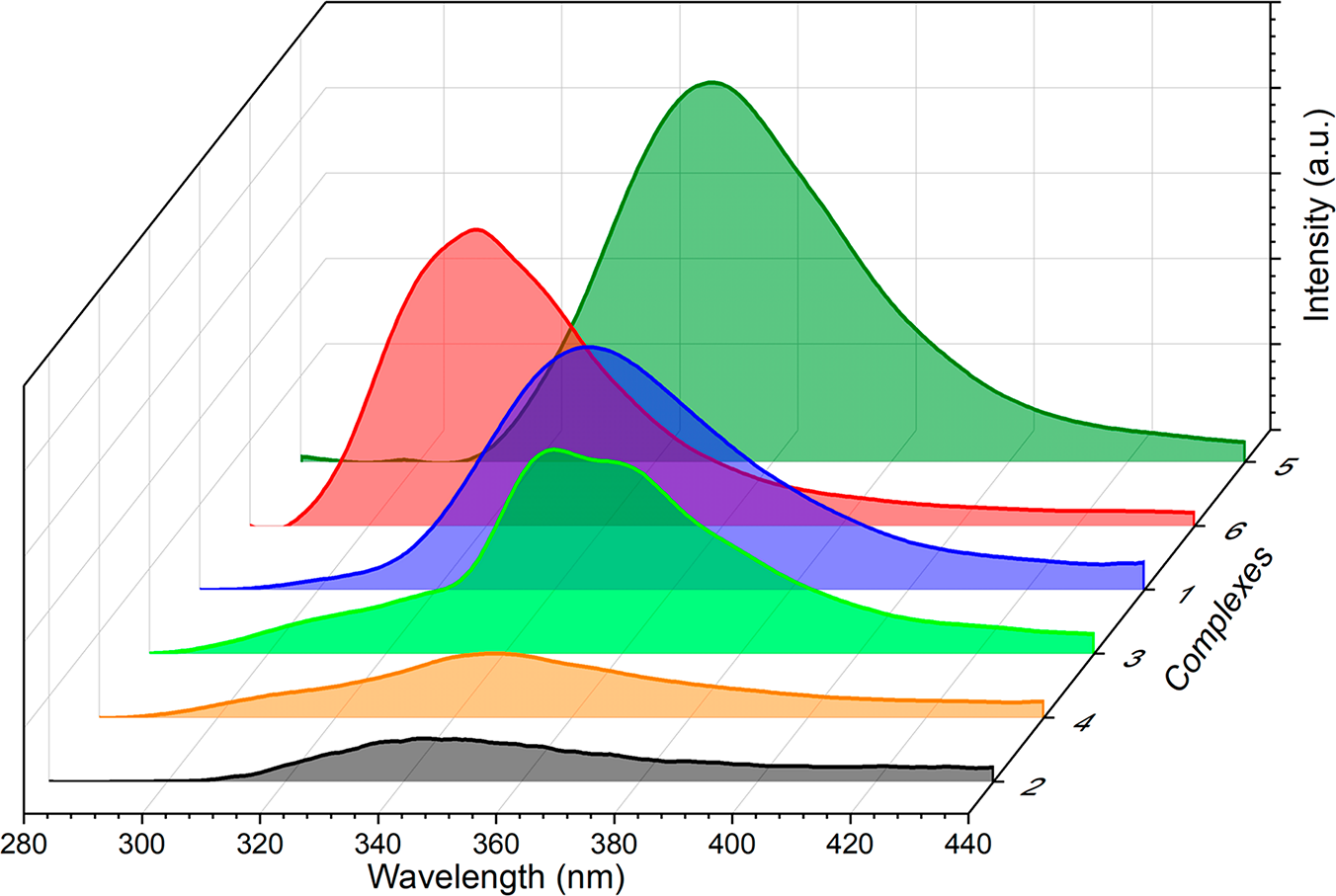
Photoluminescence spectra of complexes **1**–**6** in MeOH solution excited at their corresponding emission maxima.

All of the measurements were performed at 298 K, in MeOH as solvent, and using the concentration extracted from the UV–vis data. The emission spectra of complexes **1**–**6** show that the emission intensity increases in that order: **2** < **4** < **3** < **1** < **6** < **5** (Figure 12). The spectrum of **3** has an unfolded emission band centered at 340 and 353 nm, while the rest of the complexes have only one predominant band centered between 316 and 356 nm. The resultant emission color (λ_max-em_) for complexes **1**–**6** at the selected λ_exc_ is blue, as displayed in CIE1931 chromaticity diagrams.^63^ The effect on the emission spectrum after coordination of the ligands to the Hg(II) center has been analyzed by comparing the emission of the ligands with the corresponding complex being sorted in groups of three including the pair of ligands (Pip + dPy) with their corresponding complex (**1**–**6**), all being excited at the emission maxima of the complexes. These spectra are depicted in Figure S25 of the Supporting Information and show the quenching or enhancement effect after complexation of the ligands. Complex **6** is the only keeping the emission intensity (Supporting Information: Figure S25d). On the other hand, complexes **3** and **5** exhibit an emission enhancement effect (Supporting Information: Figures S25c and e), while complexes **1**, **2**, and **4** display a quenching effect (Supporting Information: Figures S25a, b, and d).

The fluorescence quantum yield (ϕ) is defined as the ratio between the number of photons emitted and the number of photons absorbed and describes how efficient the sample is in converting the excitation light into photon emission.^64^ By comparison with a reference (standard), the relative quantum yield (ϕ_s_) of the selected product can be obtained.^65^

The quantum yield of **1**–**6** is calculated using eq 1
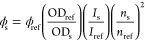
1where ϕ is the quantum yield, OD is the optical density (or absorbance) at the excited wavelength, *I* is the area under the curve of the emission spectra, and *n* is the refractive index of the solvent. In this study, l-tyrosine has been used as the standard (φ_ref_ = 0.14)^66^ and OD_ref_ and *I*_ref_ values have been obtained from a solution at a concentration of 1.01 × 10^–4^ M in Milli-Q water as solvent (*n*_ref_ = 1.3325)^67^ at 298 K. The values of *A*_s_ and *I*_s_ of HPip and dPy ligands as well as those of **1**–**6** have been measured from solutions of 9.95 × 10^–7^ M (HPip) and ∼6.16 × 10^–6^ M (dPy and complexes **1**–**6**) in MeOH as solvent (*n*_s_ = 1.3314)^68^ at 298 K. The values of ϕ_s_ obtained for compounds **1**–**6** are 0.027 (**1**), 0.011 (**2**), 0.968 (**3**), 0.051 (**4**), 0.370 (**5**), and 0.047 (**6**) (Table 14).

Comparing the ϕ_s_ values sorted by the number of dPy *N*-donor sites, **1** has a slightly higher efficiency than **2** but is constant in the same order of magnitude. In the complexes with chelating ligands (**3**–**6**), even if chelation enhanced fluorescence (CHEF) competes with steric crowding, the bigger size of the Hg(II) center enables geometrical distortions without severe alterations of the Hg–*N* bond lengths.^69^ In this sense, the non-negligible better ϕ_s_ of complex **5** with respect to **6** is probably caused by the greater symmetry and rigidity of the terpy linker compared to dpa, in which the aliphatic −(CH_2_)– groups allowed severe bond length distortion of the [HgO_4_N_3_] *core* and, thus, facilitated a nonradiative decay. Finally, the largest difference in ϕ_s_ is reflected in complexes **3** and **4**, with **3** having a significantly larger value. Despite that the 1,10-phen ligand has more rigidity than 2,2′-bipy, it is known that it has close-lying ππ* and nπ* singlet excited states, with nπ* excited states promoting its nonradiative decay and consequently vanishingly low emission ϕ_s_.^70^ On the other hand, the 2,2′-bipy ligand exhibited ππ* relaxation in polar solvents as MeOH.^71^ This is maintained after complexation with d^10^ metal ions, which do not have low-lying MLCT electronic levels and, thus, keep ligand centered transitions. Only 1,10-phen functionalization, especially in 2,9-positions, led to significantly better ϕ_s_ values.^70^

#### Electronic Calculations

All of the calculated UV–vis spectra of complexes **1**–**5** agree reasonably well with the experimental profiles. The shift in the theoretical absorption spectra with respect to the experimental spectra lies within the range of typical TD-DFT calculations (∼0.3 eV) and is caused by computing absorptions as vertical transitions.^72^ Only transitions with higher *f* values have been selected for the 2D color filled mapping of TDM and NTO analysis. The HOMO and LUMO outline as well as energy band gaps can be found in Figure S26 in the Supporting Information. Subsequently, the main contributor transitions, being either Hg, Pip, dPy, or a combination of them, have been analyzed for each absorption band to identify both the character and the regions involved in the electronic transition. Each set of transitions has been represented as a 2D color filled map of the TDM, and these results were confirmed by NTO analysis.

The HOMO and LUMO orbitals of **1**–**5** have π symmetry with the HOMO being distributed among the Pip ligand and the LUMO being uniformly localized over the dPy linkers. It has been previously demonstrated for Pt complexes bearing *N*,*N*- and *N*,*N*,*N*-donor ligands that this MO seclusion allowed the HOMO and LUMO energies to be separately modified by functionalization, and thus, introduction of substituents on the dPy or Pip ligands could selectively tune the band gap.^73,74^

**Figure 13:**
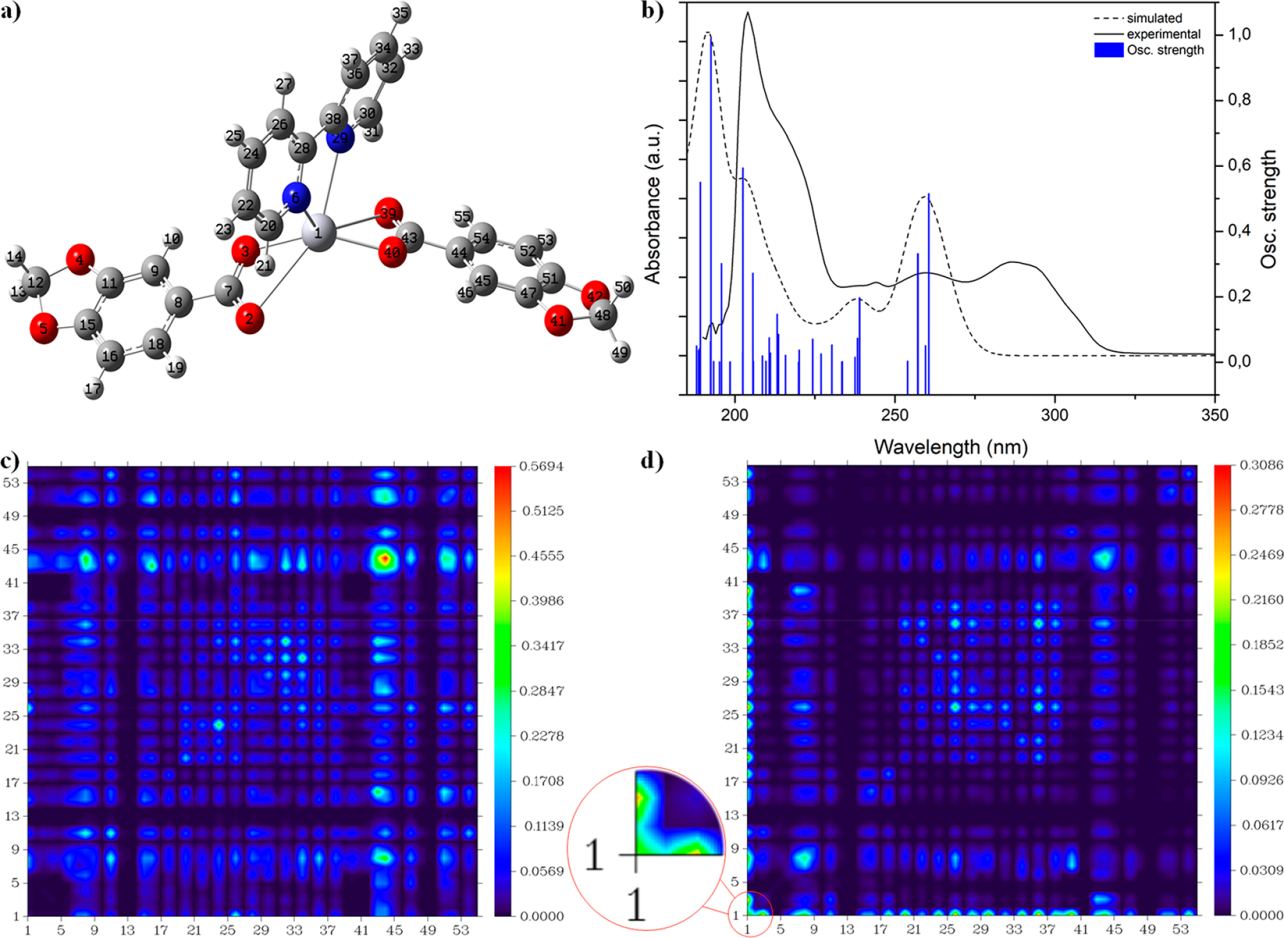
(a) Molecular structure and atom labeling of compound **3**. (b) Experimental and simulated UV–vis spectra of **3**. 2D color filled mapping of TDM for transitions 3 (c) and 33 (d), in detail metal *core* region around the Hg(II) center (*label 1*).

Analysis of complex **3** has been provided in Figure 13 to exemplify the electronic analysis. The optimized molecular geometry of **3** with the corresponding atom labeling scheme is displayed in Figure 13a. The calculated UV–vis spectrum matches with the experimental results (Figure 13b), and the primary significant transitions have been split into the ligands and Hg(II) contributions. Figure 13 shows the 2D color filled map of the TDM for transitions 3 (Figure 13c) and 33 (Figure 13d), which are representative of ligand centered (LC) and Hg(II) involved transitions. Focusing on the diagonal of transition 3 centered at 257 nm (Figure13c), the region around the atom labeled 43 stands out from the rest, indicating where the electronic transition occurs, which corresponds to the Pip ligand, in particular to the carboxylate and the subsequent carbons from the aromatic ring, congruent with the NTO analysis results (Supporting Information: Figure S34). Both the LMCT and MLCT transitions are predominantly responsible for the higher energetic transitions being located around 200 nm. At lower energies, the more red-shifted bands in the spectra arise from Pip in complexes **1**, **2**, and **3**, while, in those containing more pi electron rich ligands (1,10-phen and terpy), dPy are responsible for such transitions (**4** and **5**). Regarding metal centered transitions, these results display that MLCT transitions rise from Hg → Pip in complexes **1**–**4** (identified between 203 and 212 nm), while in **5** this contribution is not observed. The role of dPy on MLCT transitions for **1**–**4** is neglectable, since dPy are not involved in them in complexes **1** and **2** and only minor contributions from 2,2′-bipy and 1,10-phen can be observed in **3** and **4**. What should be noted is the different behavior displayed in complex **5**, in which the terpy ligand facilitates the MLCT to be from Hg → terpy, causing a remarkable bathochromic shift on absorption (253 nm). This difference is probably originated from the better π-acceptor character of the terpy ligand with respect to the remaining dPy. The Supporting Information displays the complete electronic analysis of the remaining complexes with additional 2D plots (Supporting Information: Figures S27–S31 and Table S2) and NTO analysis (Supporting Information: Figures S32–S36).

## Conclusions

We synthesized and characterized a series of Hg(II) coordination complexes bearing HPip and *N*-, *N*^*N*-, and *N*^*N*^*N*-donor ligands, revealing that the use of the Hg(II) metal node allowed the primacy of the geometric demands of the linkers with coordination numbers 6 and 7 to be accommodated. Despite that the Pip linker demonstrated its ability to form polymeric structures by μ_2_-η^2^:η^1^ coordination modes (**1** and **2**), both the increasing bulkiness of the dPy ligands and their chelate effect afforded the predominance of the dPy over the Pip ligand (**3**–**6**) and drove the formation of discrete structures being two monomers and a dimer. A comparison of the steric effects evinced that a slight increase in the bulkiness of dPy is sufficient to promote the formation of a monomer instead of a dimer, along with bulkier ligands *inter alia* terpy and dpa, which unavoidably formed monomers. These complexes were associated by weak C–H···O and π···π interactions, which is reflected in the aggregation patterns, except for complex **3** that avoided aggregation up to ∼1 × 10^–4^ M and presented less and weaker interactions. Furthermore, aggregation drove significant shifts on absorption spectra, especially remarkable for **5** and **6**. The experimental and theoretical photophysical study revealed that **1**–**5** have a localized HOMO on Pip while the LUMO is over the dPy emerging as appropriate candidates for band gap modulation. The 2D and NTO analysis evidences that less energetic absorptions are promoted by Pip in the presence of 3-phpy and 4-phpy, while, from 2,2′-bipy and following, dPy linkers are responsible for such absorptions. Finally, the LMCT transitions lie between 203 and 212 nm originated from Hg → Pip in **1**–**4**, while in complex **5** such a transition arises from Hg → terpy, causing a bathochromic shift up to 253 nm.

## Supporting Information Available

Further details of ESI^+^-MS, TG-DTA, FTIR-ATR, ^1^H NMR, ^13^C{^1^H}, and DEPT-135 NMR experiments as well as coordinates from the DFT geometry optimizations and TD-DFT calculation results (PDF)

## Accession Codes

CCDC 2043890–2043895 contain the supplementary crystallographic data for this paper. These data can be obtained free of charge via www.ccdc.cam.ac.uk/data_request/cif, or by emailing data_request@ccdc.cam.ac.uk, or by contacting The Cambridge Crystallographic Data Centre, 12 Union Road, Cambridge CB2 1EZ, UK; fax: +44 1223 336033.
