# Supplementary material for: Steric and Electronic Effects on the Structure and Photophysical Properties of Hg(II) Complexes
Source: Inorg Chem. 2021 Feb 25;60(6):3851–70. doi: 10.1021/acs.inorgchem.0c03640 (PMC8483444; doi:10.1021/acs.inorgchem.0c03640)
Supplement: Supplementary file 1 — ic0c03640_si_001.pdf [file ic0c03640_si_001.pdf]

Supporting Information

Steric and electronic effects on the structure and photophysical properties of Hg(II) complexes

Francisco Sánchez-Férez [a], Joaquim Mª Rius-Bartra[a], Teresa Calvet[b], Mercè Font-Bardia[c], Josefina Pons[a,].*

[a]Departament de Química, Universitat Autònoma de Barcelona, 08193-Bellaterra, Barcelona, Spain

[b]Departament de Mineralogia, Petrologia i Geologia Aplicada, Universitat de Barcelona, Martí i Franquès s/n, 08028 Barcelona, Spain.

[c]Unitat de Difracció de Raig-X, Centres Científics i Tecnològics de la Universitat de Barcelona (CCiTUB), Universitat de Barcelona, Solé i Sabarís, 1-3, 08028 Barcelona, Spain.

HR-ESI-MS

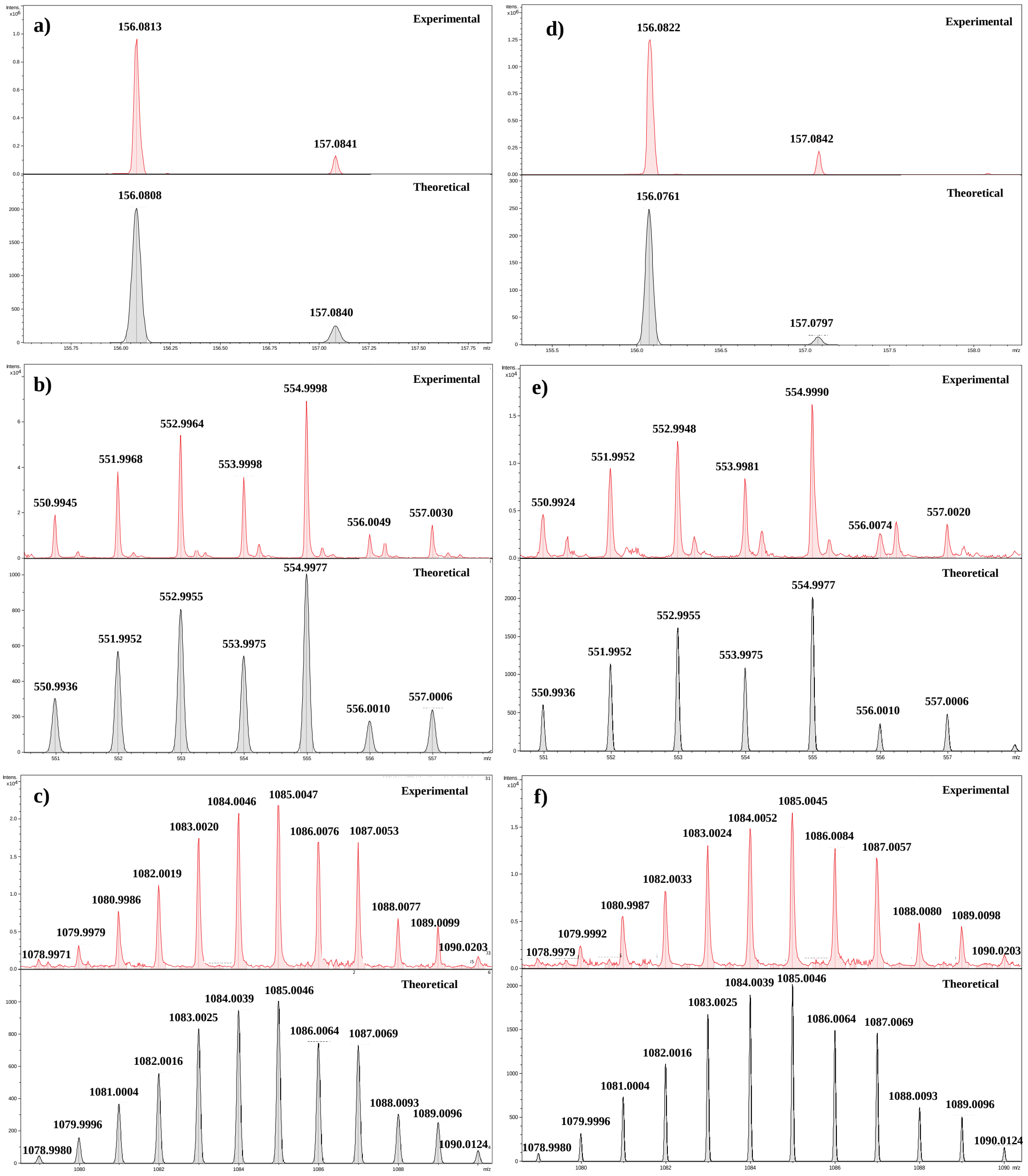

Figure S1. HR-ESI$^+$-MS spectra of compounds **1** (a-c) and **2** (d-f), corresponding to [3-phpy+H]$^+$ (a), [4-phpy+H]$^+$ (d), {[Hg(Pip)$_2$]+Na}$^+$ (b and e) and {[Hg(Pip)$_2$]$_2$+Na}$^+$ (c and f) fragments.

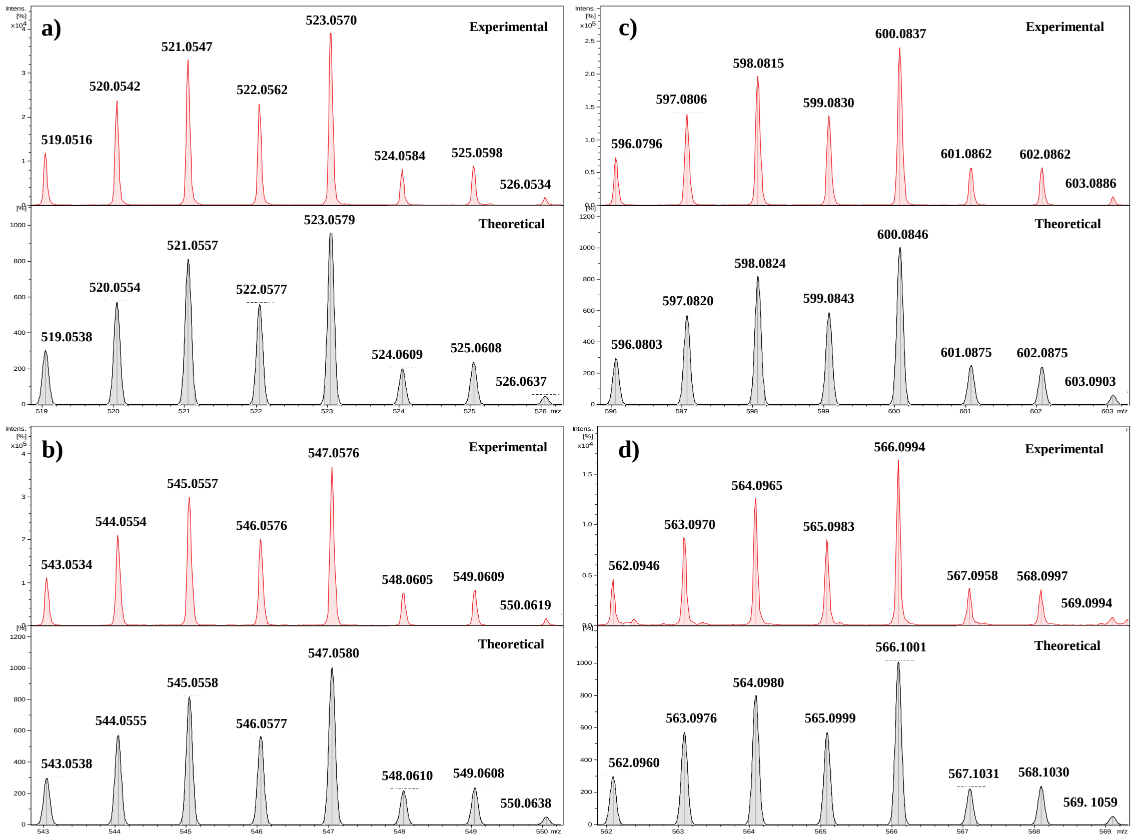

Figure S2. HR-ESI$^+$-MS spectra of compounds **3** (a), **4** (b), **5** (c) and **6** (d), corresponding to $\{[Hg(Pip)(dPy)]\}^+$ fragments.

TG/DTA spectrum

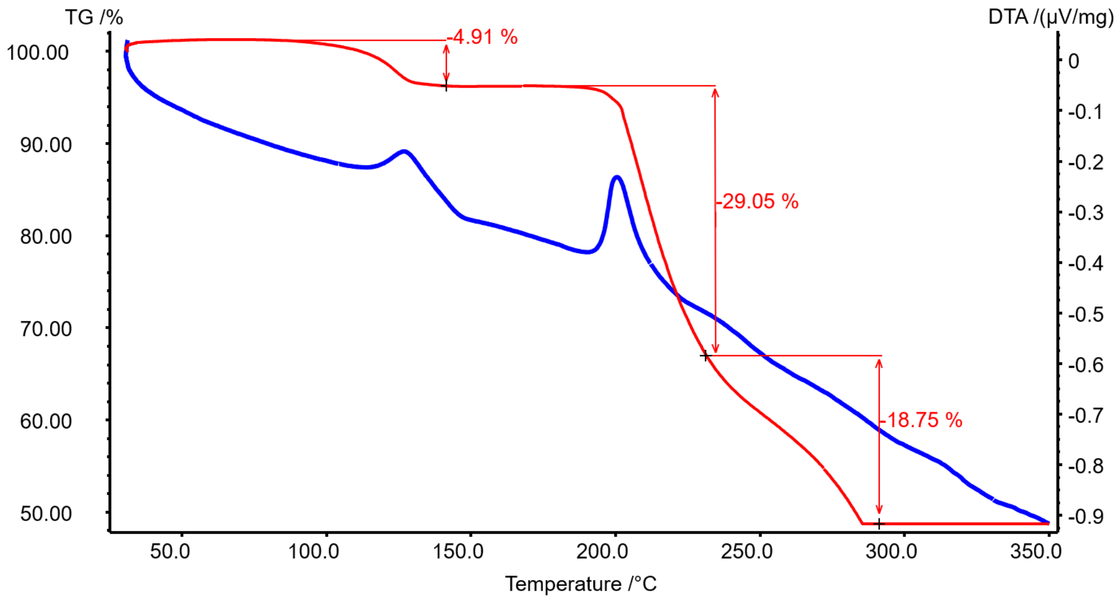

Figure S3. TG (red line)/DTA (blue line) plot of $[Hg(Pip)_2(terpy)]\cdot EtOH$ (**5**) between 25 ºC and 350 ºC.

FTIR-ATR spectroscopic data

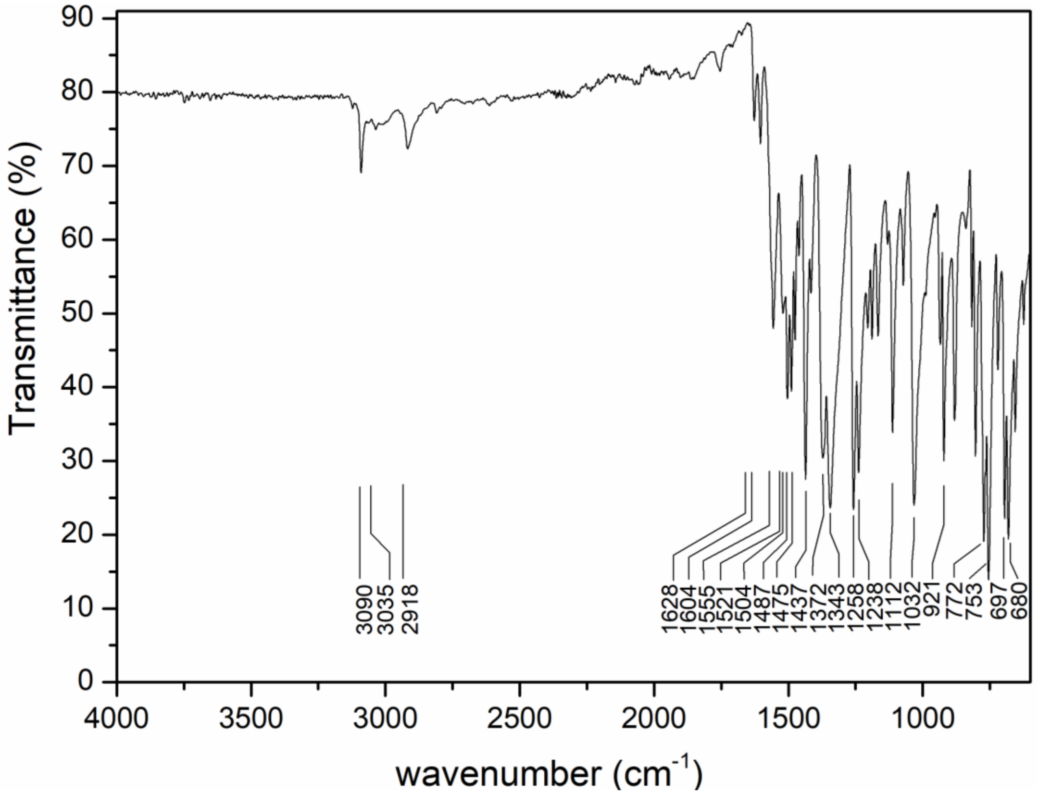

Figure S4. FTIR-ATR spectrum of compound [Hg(μ-Pip)$_2$(3-phpy)]$_n$ (**1**)

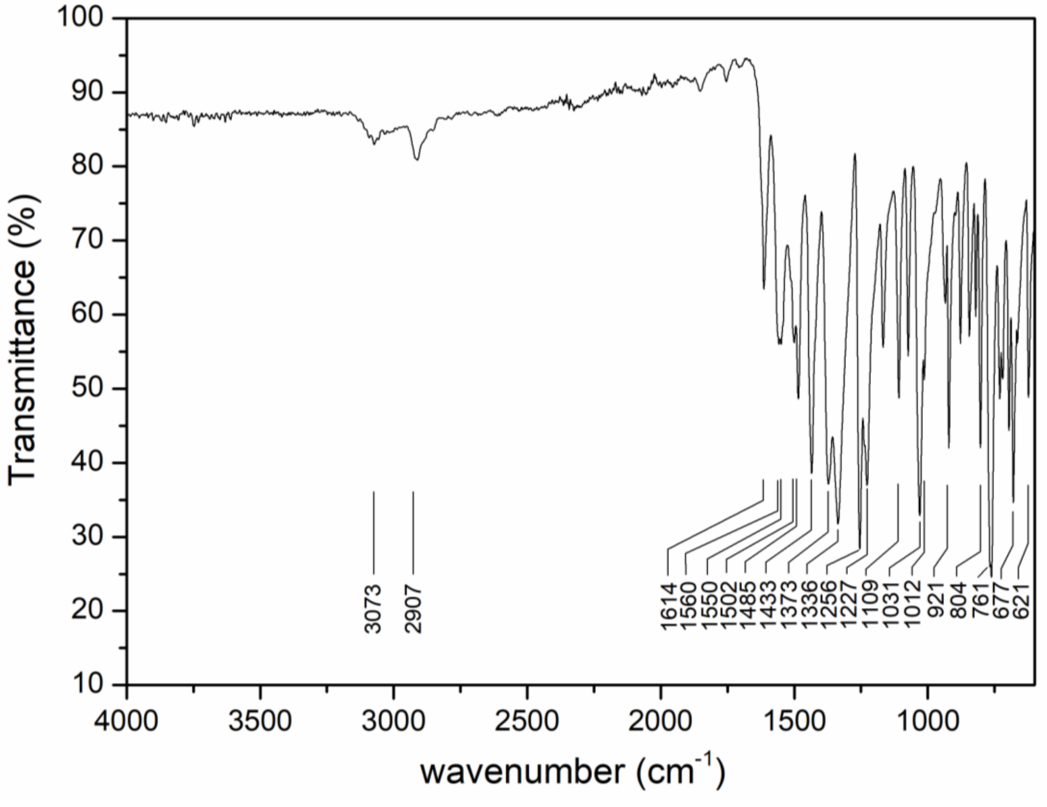

Figure S5. FTIR-ATR spectrum of compound [Hg(μ-Pip)$_2$(4-phpy)]$_n$ (**2**)

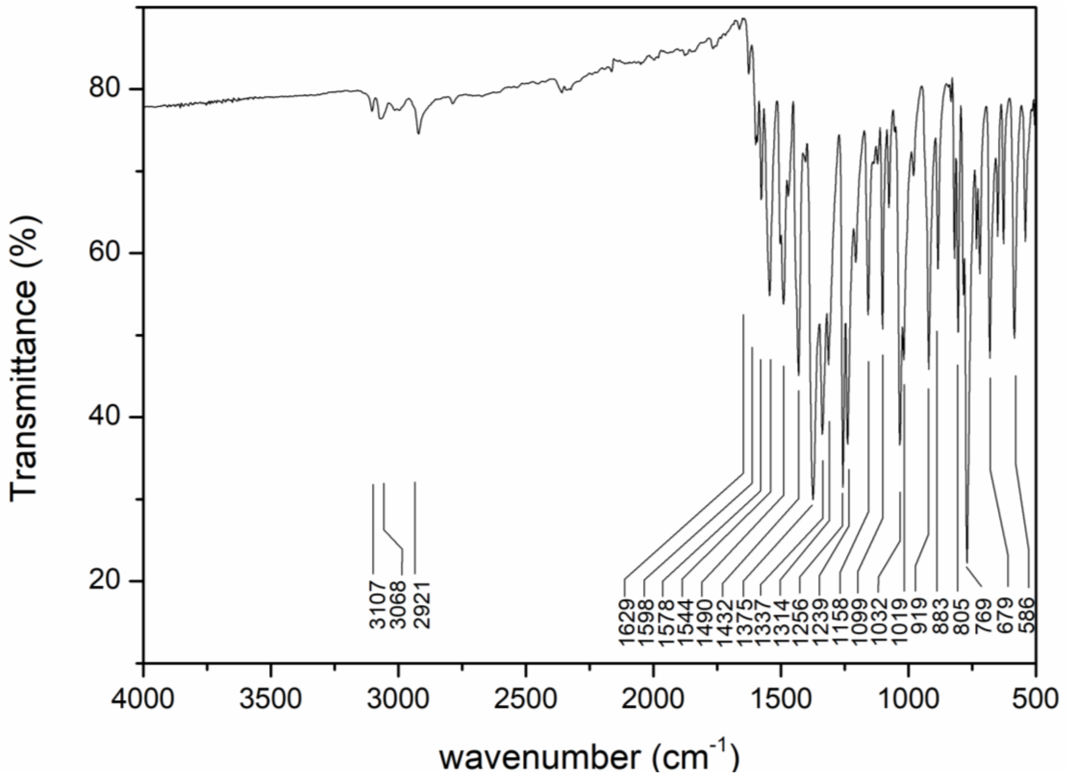

Figure S6. FTIR-ATR spectrum of compound [Hg(Pip)$_2$(2,2'-bipy)] (**3**)

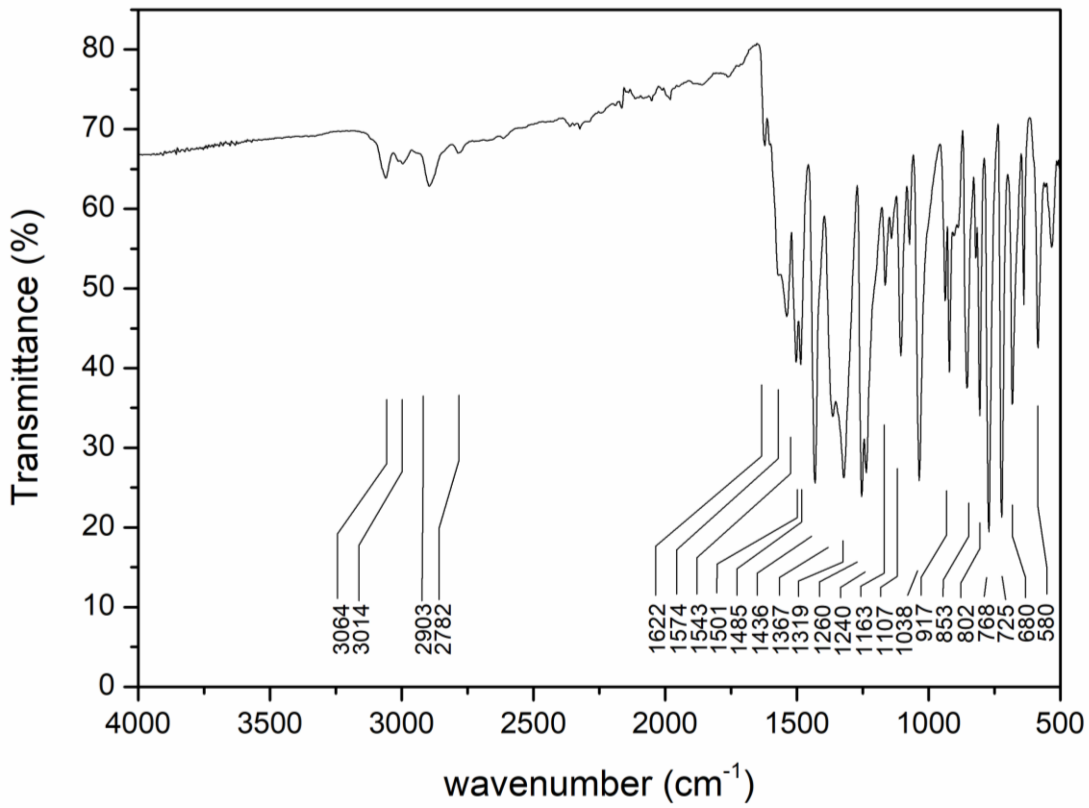

Figure S7. FTIR-ATR spectrum of compound [Hg(μ-Pip)(Pip)(1,10-phen)]$_2$ (**4**)

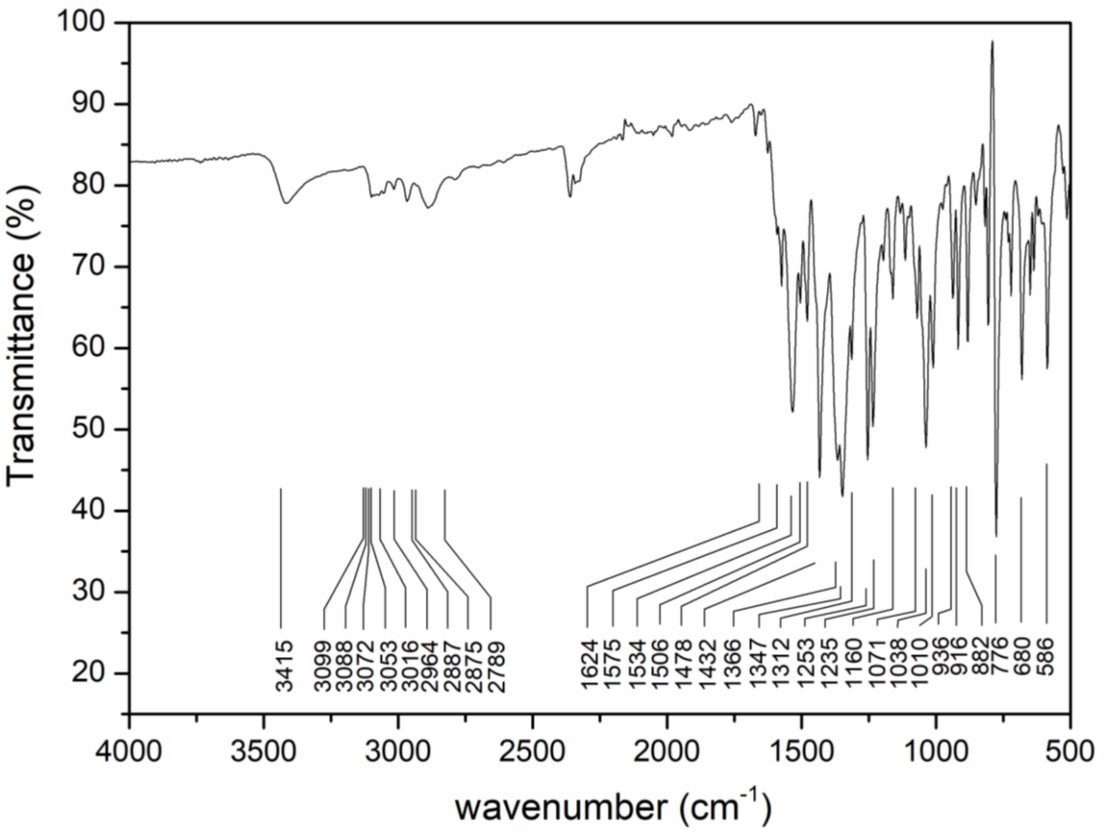

Figure S8. FTIR-ATR spectrum of compound [Hg(Pip)$_2$(terpy)]·EtOH (**5**)

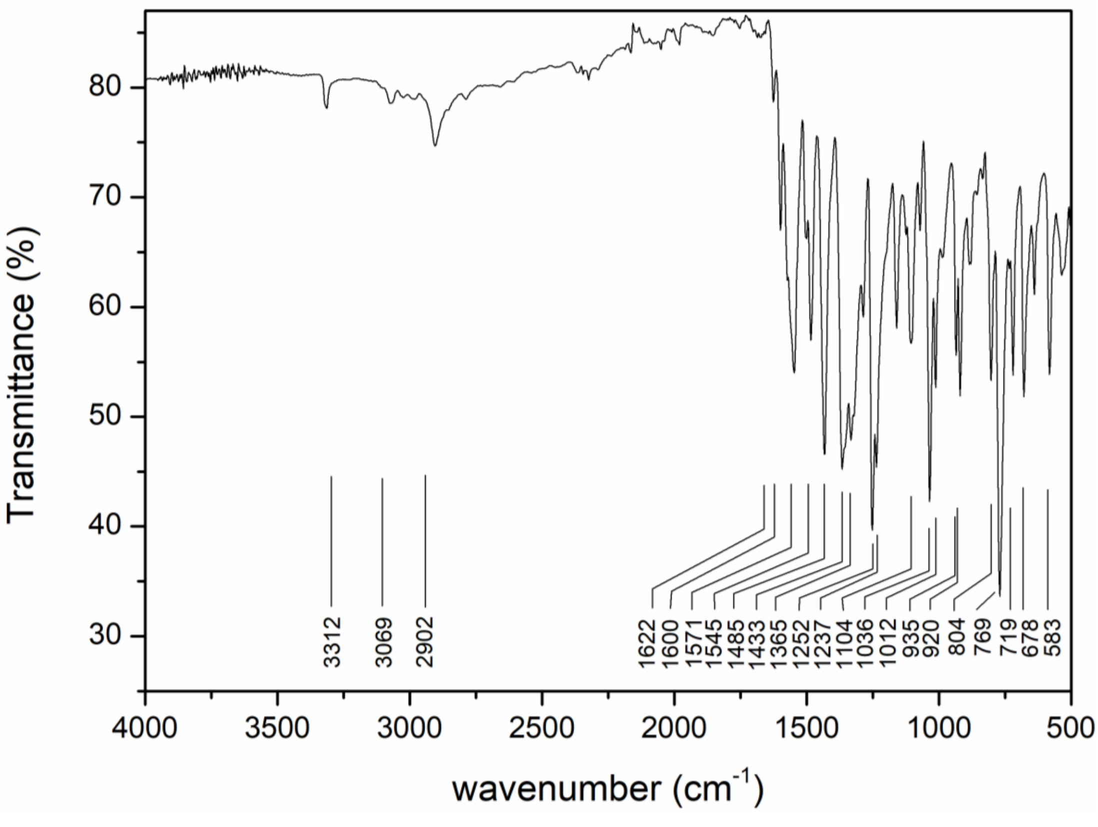

Figure S9. FTIR-ATR spectrum of compound [Hg(Pip)$_2$(dpa)] (**6**)

^{1}H NMR spectroscopic data

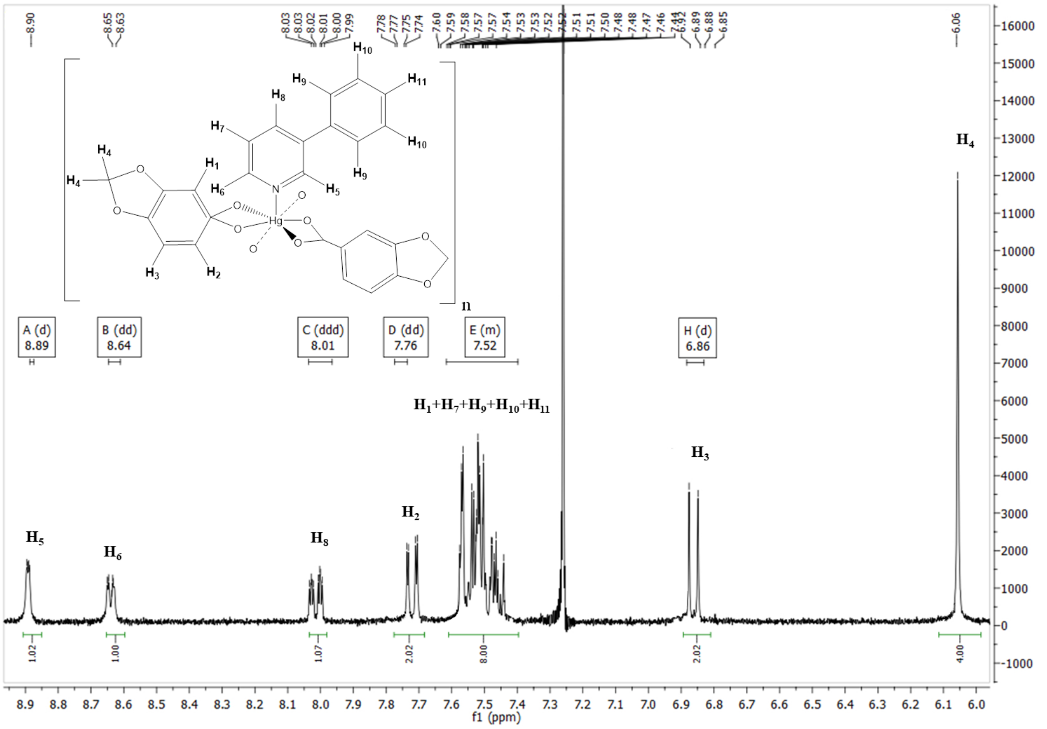

Figure S10. ^{1}H-NMR spectrum of compound [Hg(μ-Pip)$_2$(3-phpy)]$_n$ (**1**) recorded in $CDCl_3$ at 360 MHz.

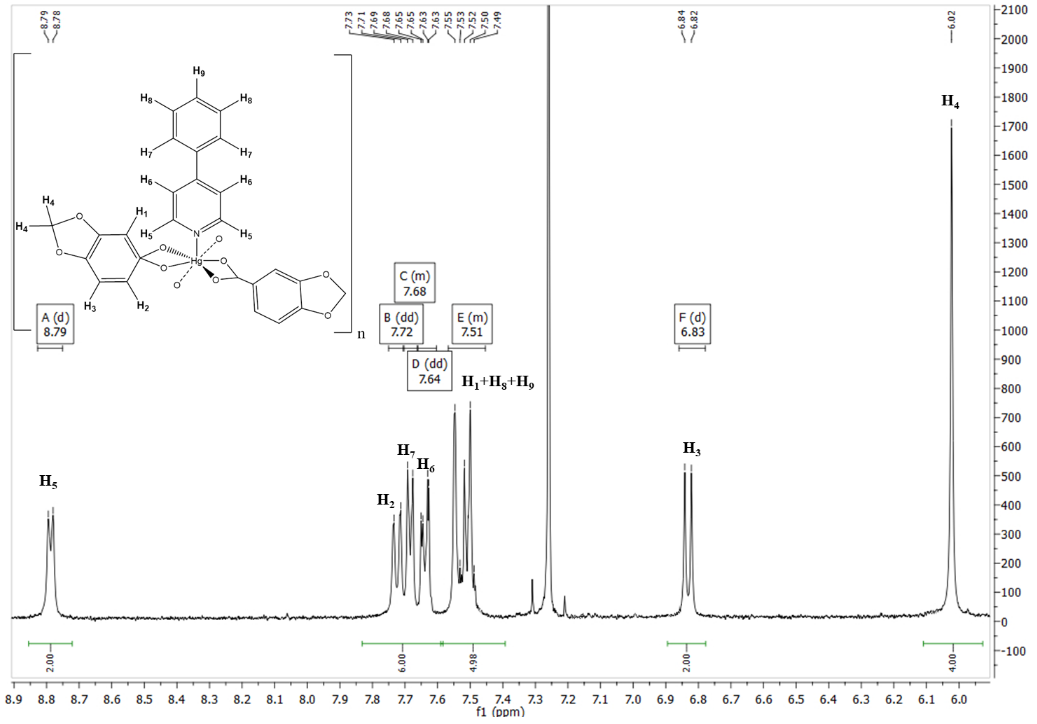

Figure S11. ^{1}H-NMR spectrum of compound [Hg(μ-Pip)$_2$(4-phpy)]$_n$ (**2**) recorded in $CDCl_3$ at 360 MHz.

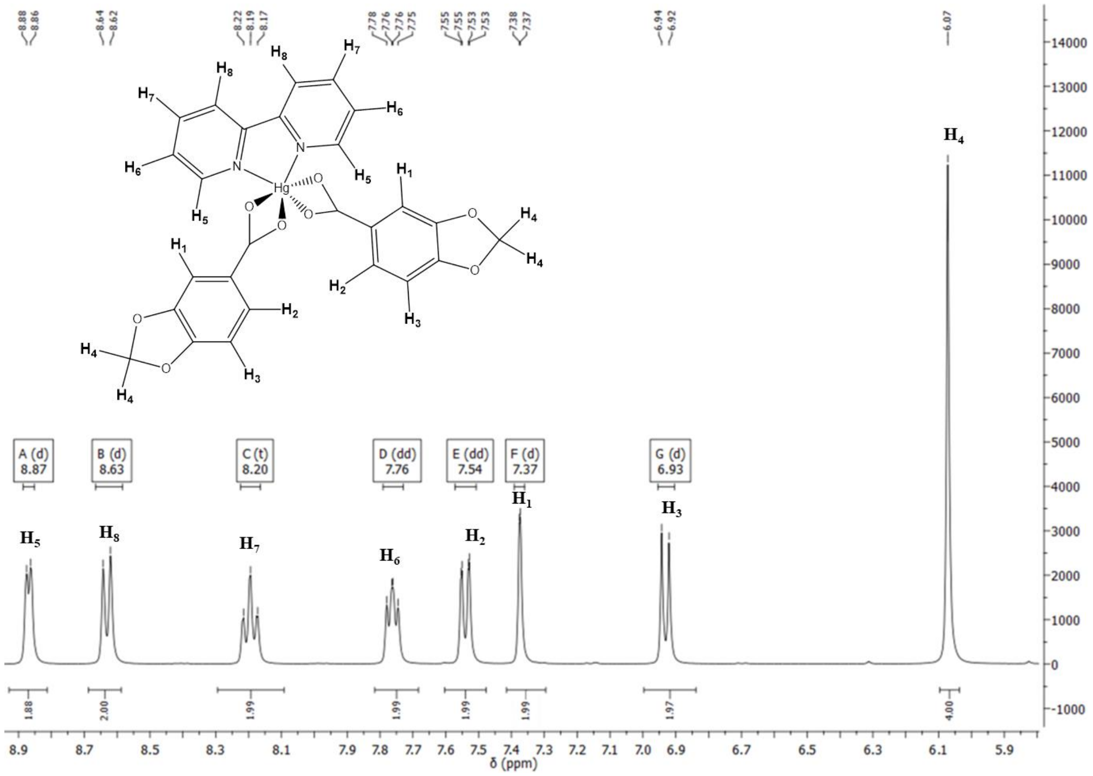

Figure S12. ^{1}H-NMR spectrum of compound [Hg(Pip)$_2$(2,2'-bipy)] (**3**) recorded in dmso-d_6 at 360 MHz.

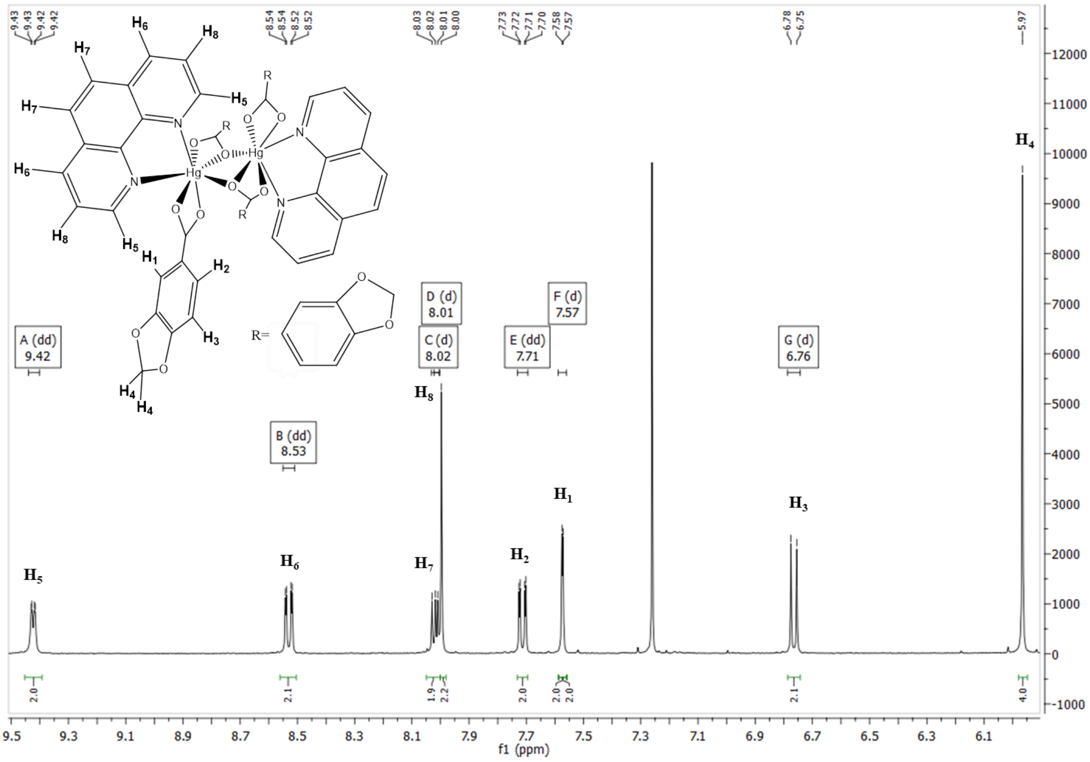

Figure S13. ^{1}H-NMR spectrum of compound [Hg(μ-Pip)(Pip)(1,10-phen)]$_2$ (**4**) recorded in $CDCl_3$ at 360 MHz.

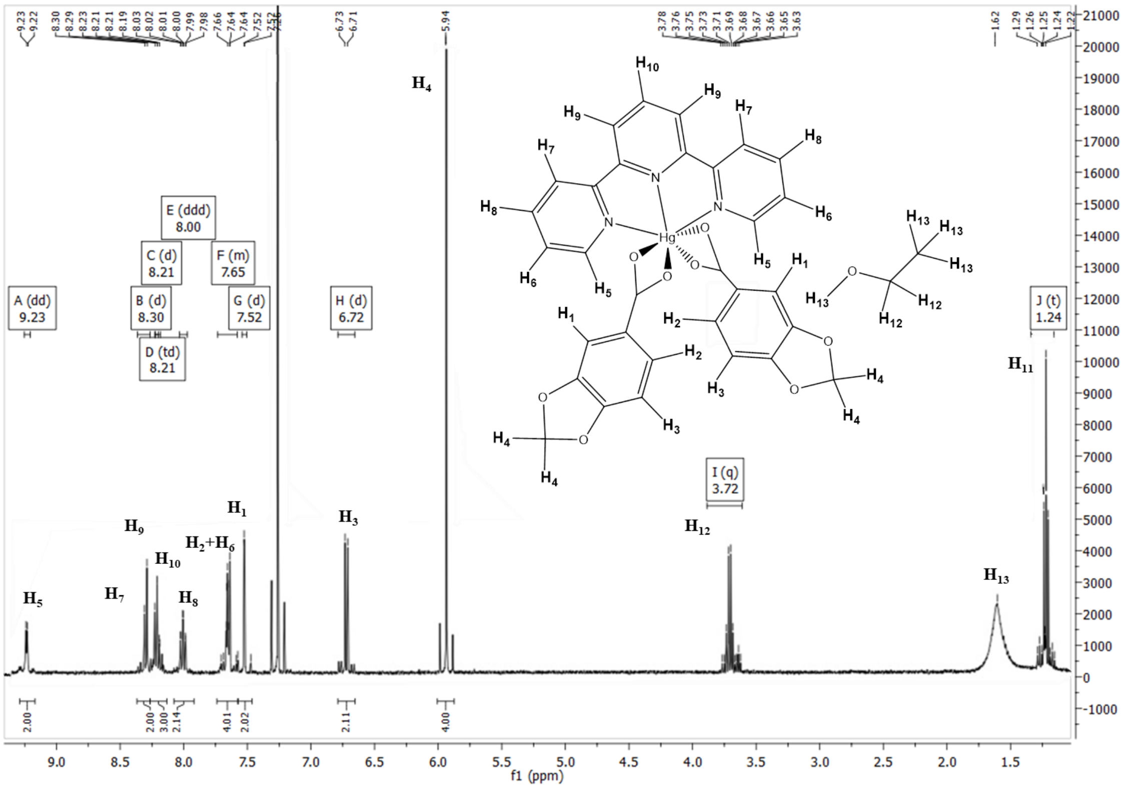

Figure S14. ^{1}H-NMR spectrum of compound [Hg(Pip)$_2$(terpy)]·EtOH (**5**) recorded in $CDCl_3$ at 360 MHz.

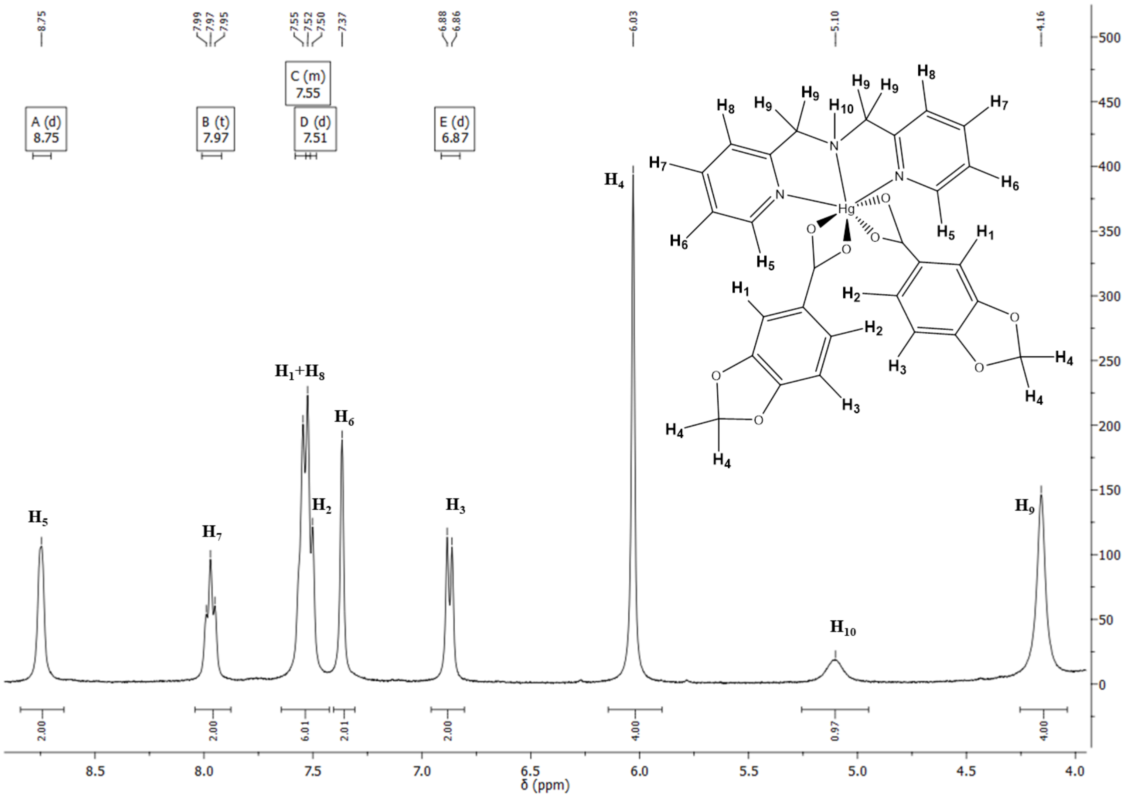

Figure S15. ^{1}H-NMR spectrum of compound [Hg(Pip)$_2$(dpa)] (**6**) recorded in dmso-d_6 at 360 MHz.

$^{13}C\{^{1}H\}$ and DEPT-135 NMR spectroscopic data

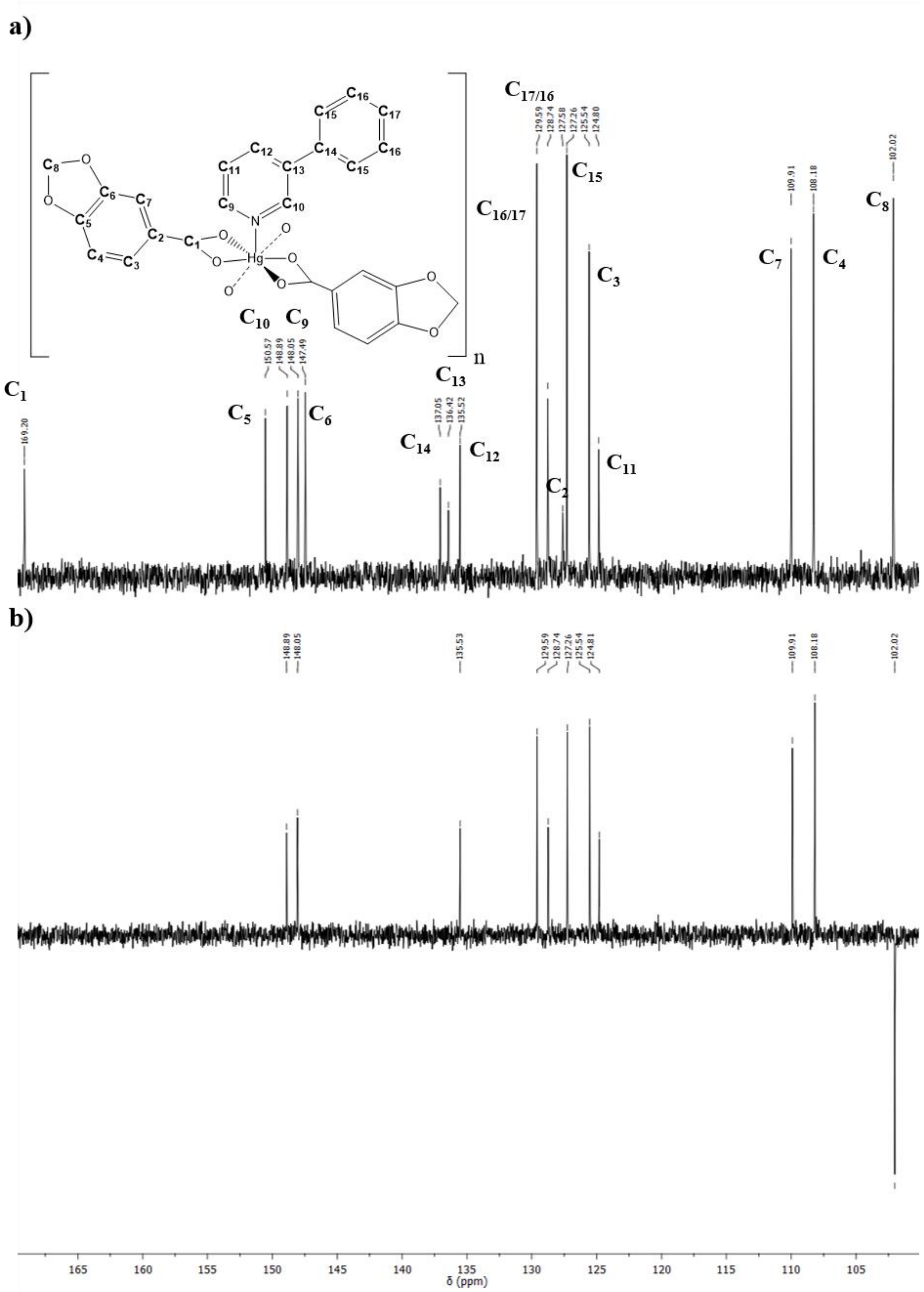

Figure S16. a) ^{13}C {^{1}H} NMR spectrum and b) DEPT-135 spectrum of compound [Hg(μ-Pip)$_2$(3-phpy)]$_n$ (**1**) recorded in dmso-d_6 at 360 MHz.

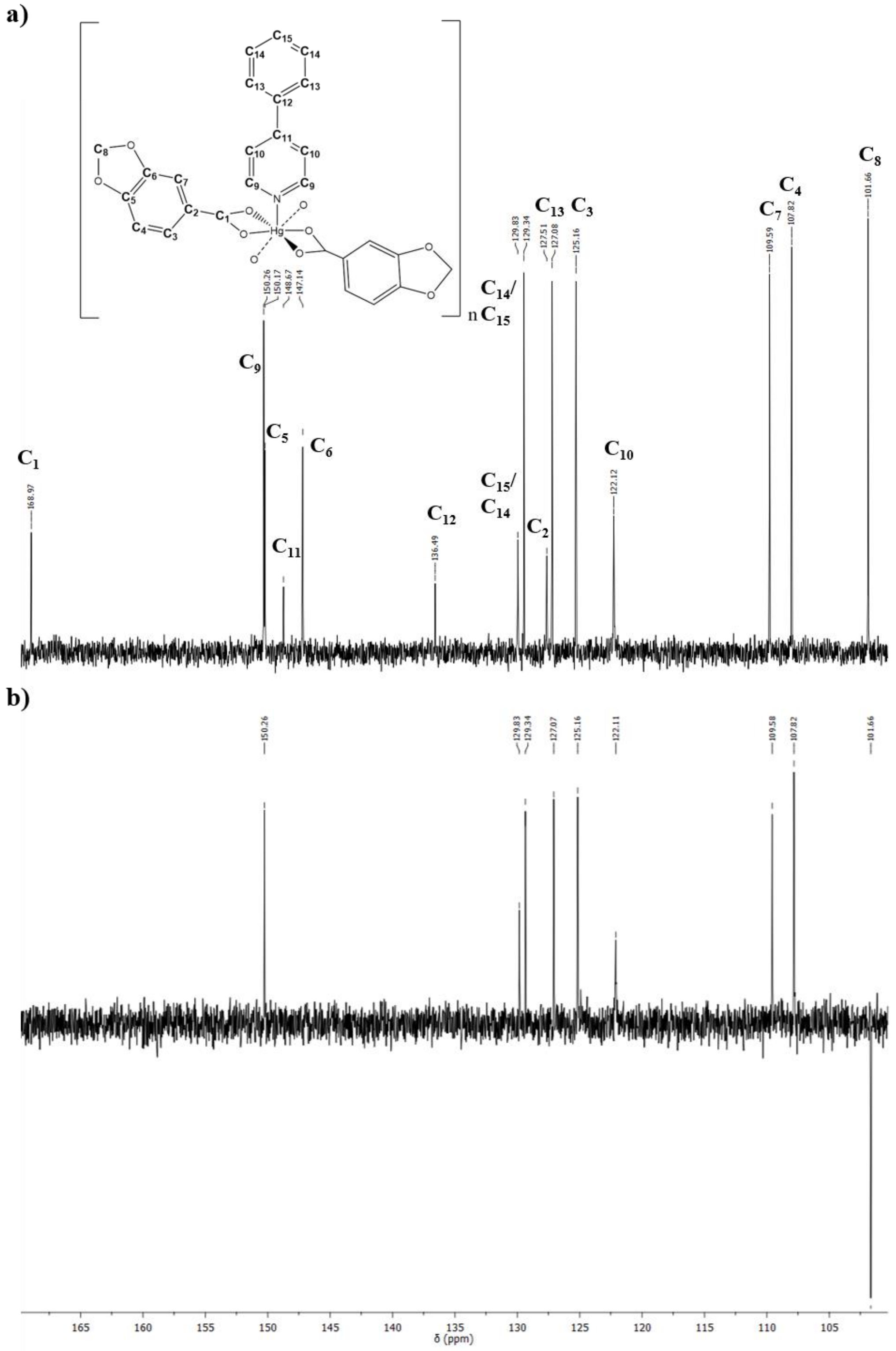

Figure S17. a) ^{13}C {^{1}H} NMR spectrum and b) DEPT-135 spectrum of compound [Hg(μ-Pip)$_2$(4-phpy)]$_n$ (**2**) recorded in dmso-d_6 at 360 MHz.

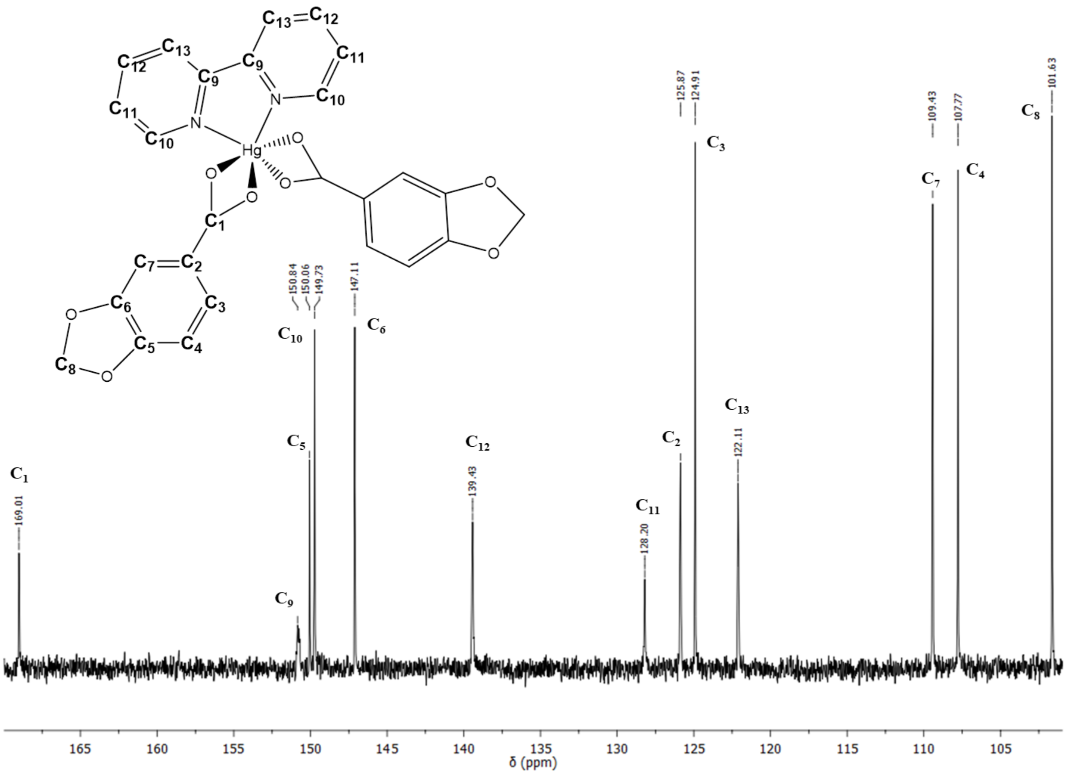

Figure S18. $^{13}C\ \{^{1}H\}$ NMR spectrum of compound [Hg(Pip)$_2$(2,2’-bipy)] (**3**) recorded in dmso-d_6 at 360 MHz.

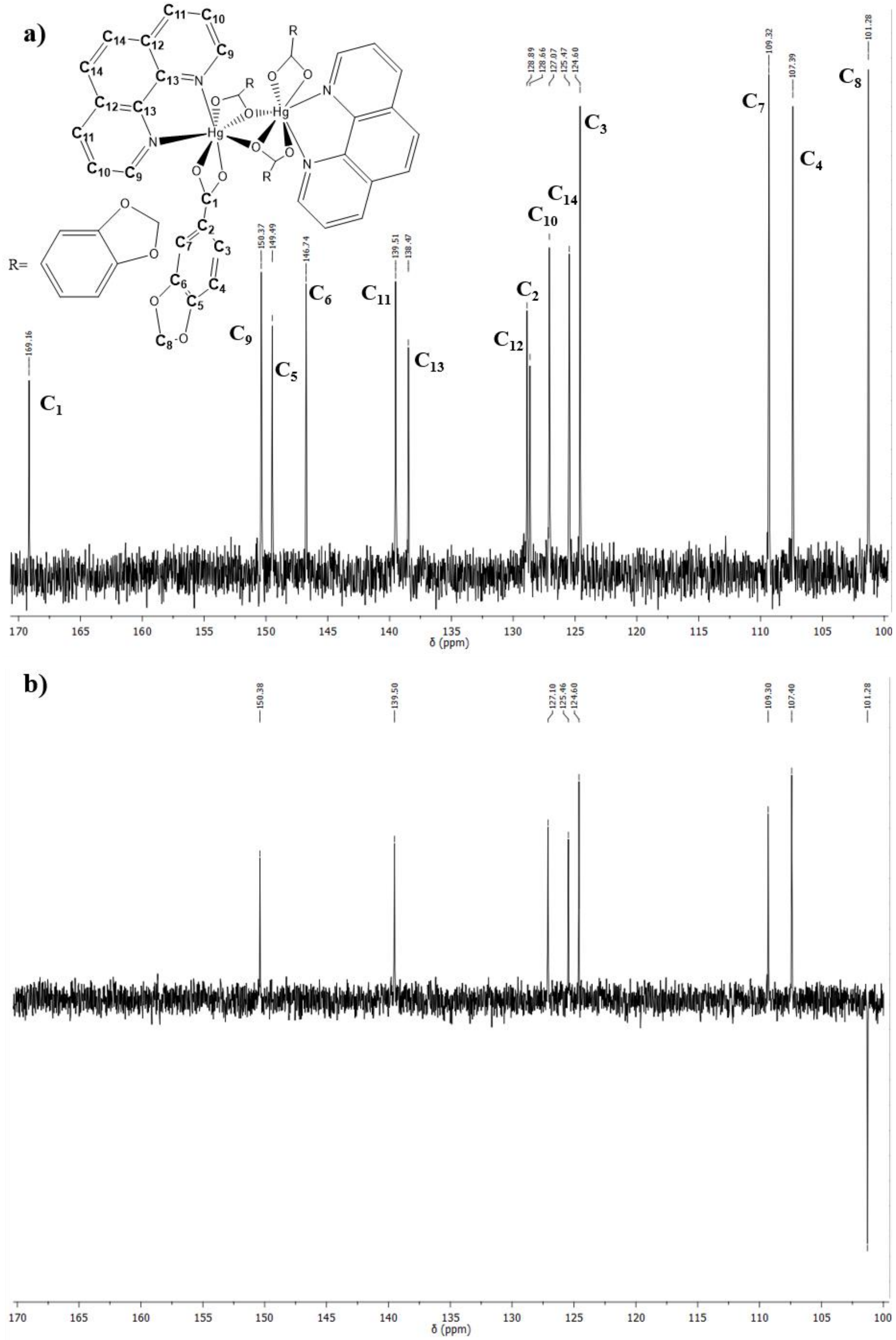

Figure S19. a) $^{13}C\{^{1}H\}$ NMR spectrum and b) DEPT-135 spectrum of compound [Hg(μ-Pip)(Pip)(1,10-phen)]$_2$ (**4**) recorded in dmso-d_6 at 360 MHz.

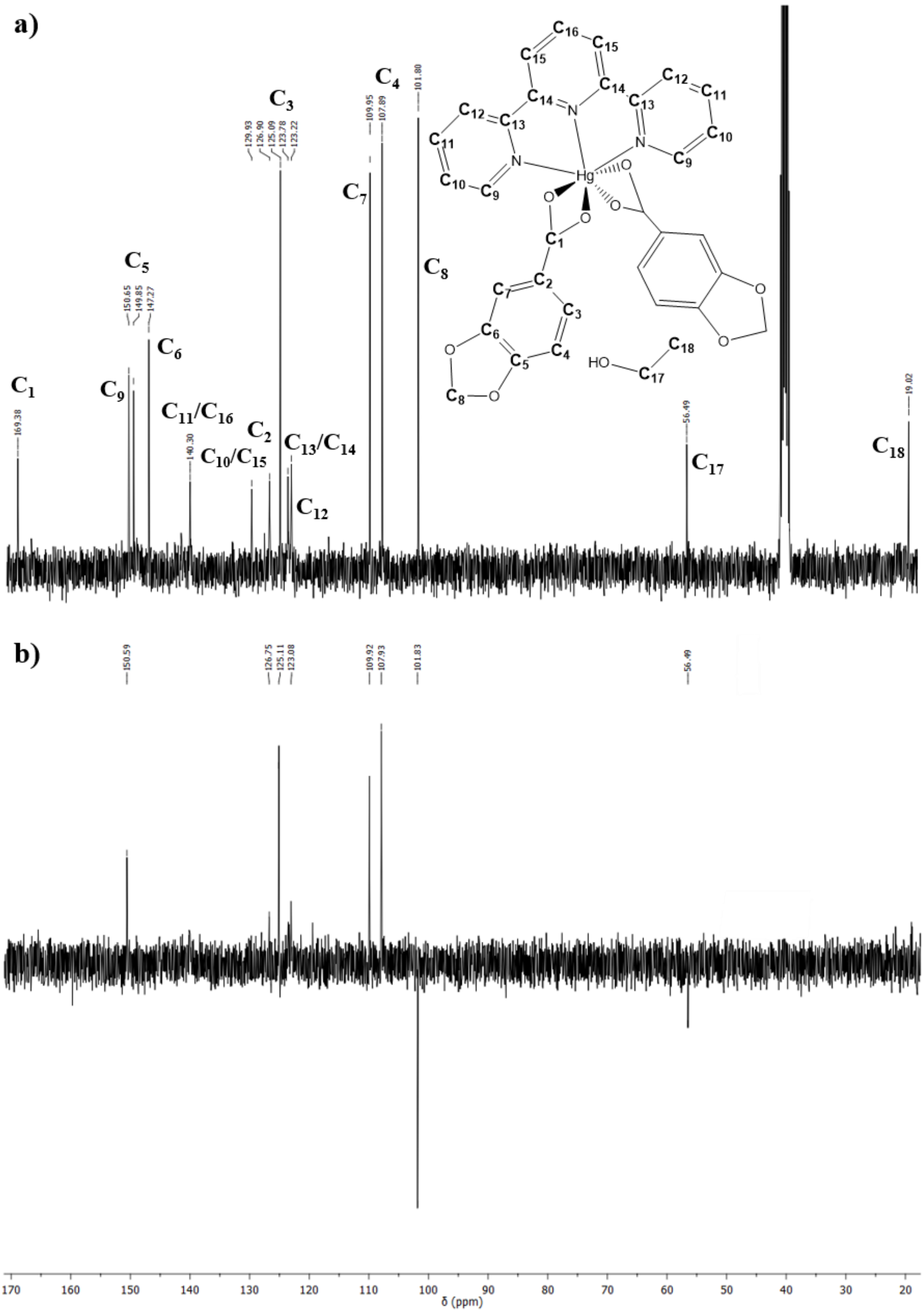

Figure S20. a) $^{13}C\ \{^1H\}$ NMR spectrum and b) DEPT-135 spectrum of compound $[Hg(Pip)_2(terpy)]$ (**5**) recorded in dmso-d_6 at 360 MHz.

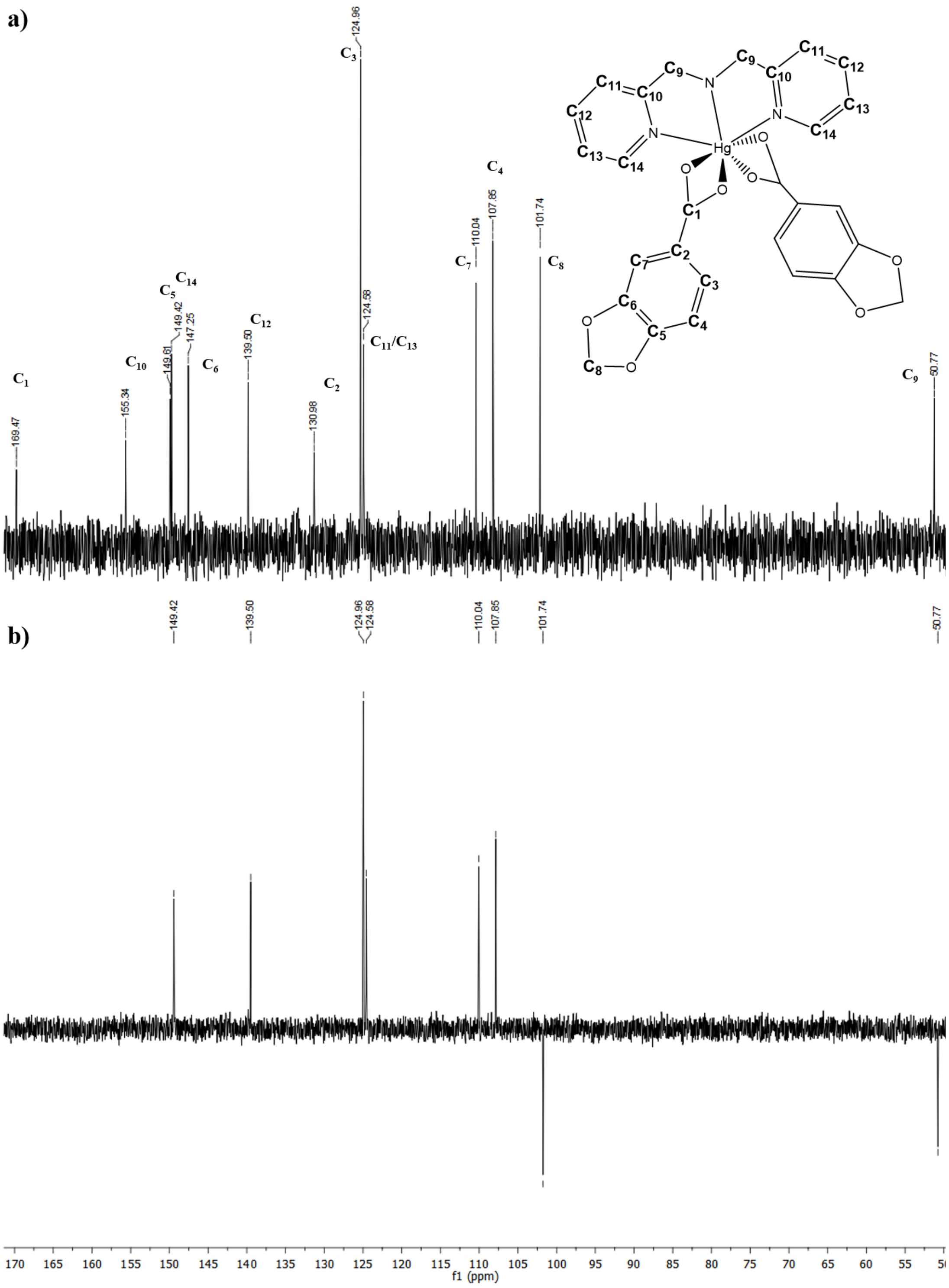

Figure S21. a) ^{13}C {^{1}H} NMR spectrum and b) DEPT-135 spectrum of compound [Hg(Pip)$_2$(dpa)] (**6**) recorded in dmso-d_6 at 360 MHz.

UV-VIS and photoluminescence data

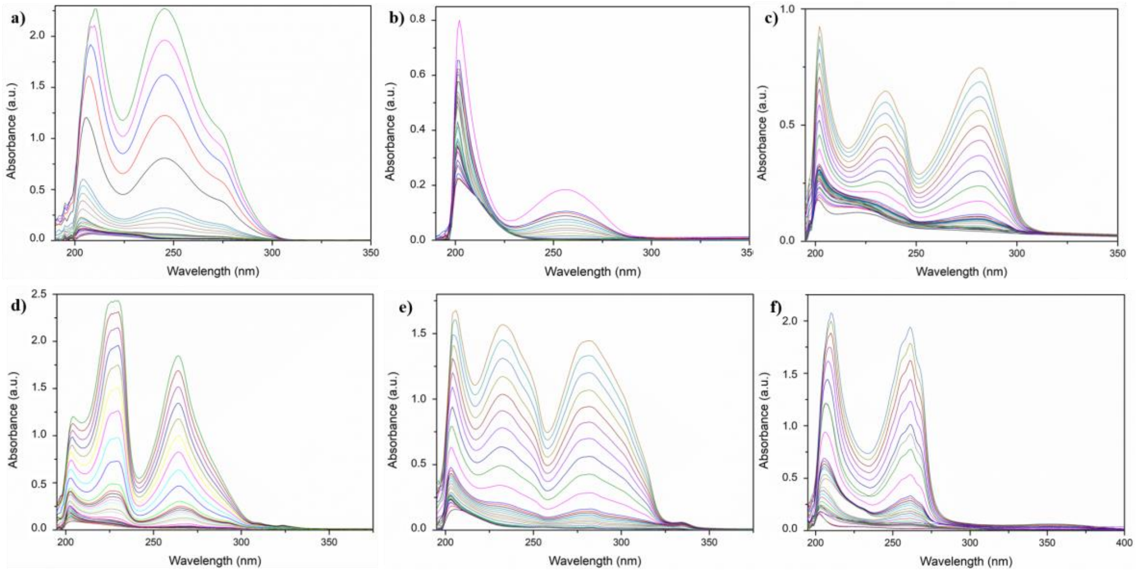

Figure S22. UV-Vis spectra of dPy ligands recorded at 298K, within a concentration range from $1\cdot10^{-9}$M to $1\cdot10^{-4}$M. (a) 3-phpy; (b) 4-phpy; (c) 2,2'-bipy; (d) 1,10-phen; (e) terpy and (f) dpa.

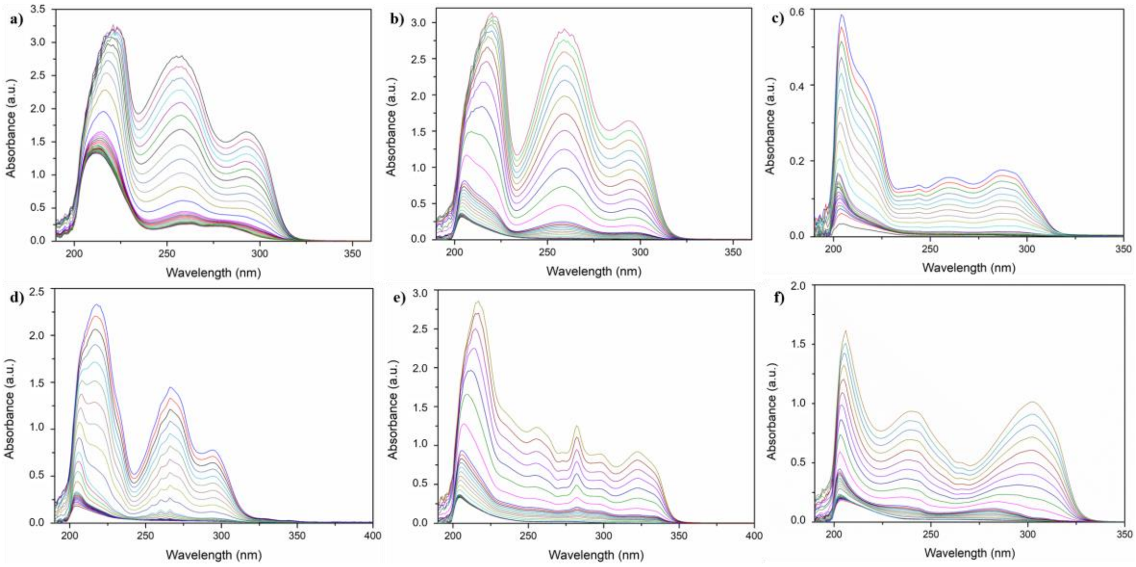

Figure S23. UV-Vis spectra of complexes **1**-**6** recorded at 298K, within a concentration range from $1\cdot10^{-9}$M to $1\cdot10^{-4}$M. (a) **1**; (b) **2**; (c) **3**; (d) **4**; (e) **5** and (f) **6**.

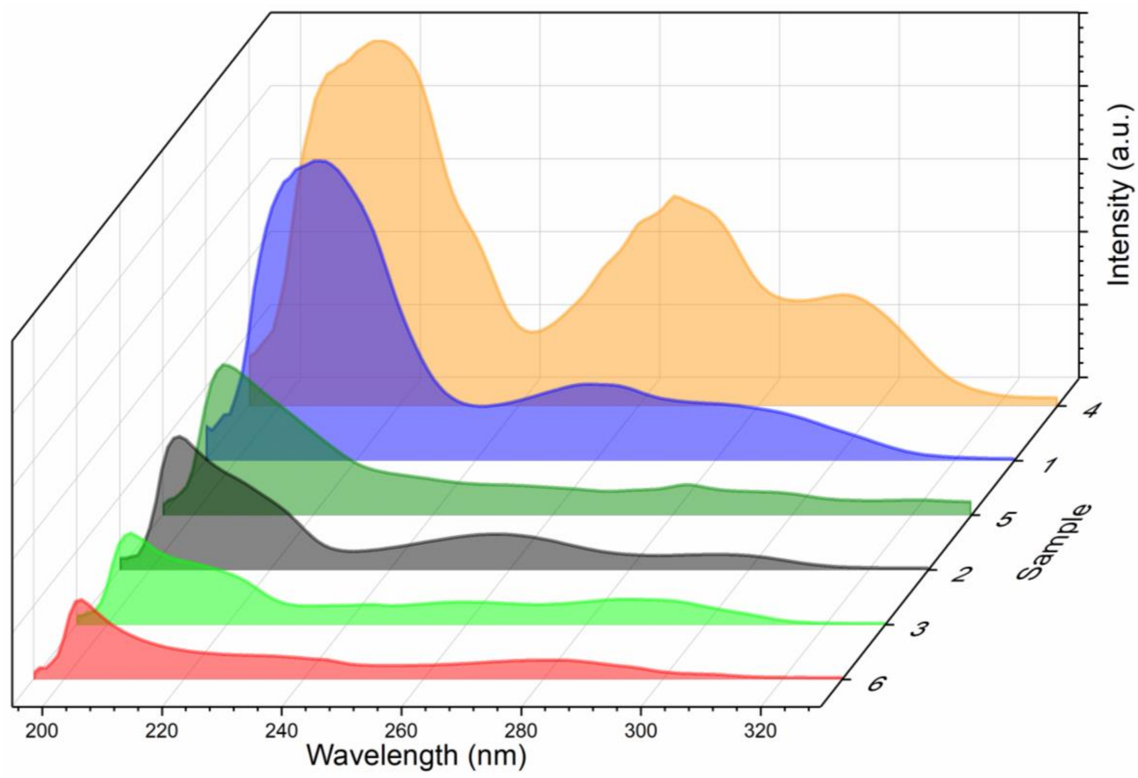

Figure S24. UV-Vis spectra of complexes **1-6** recorded at 298K at a concentration of ~$6.16 \cdot 10^{-6}$M

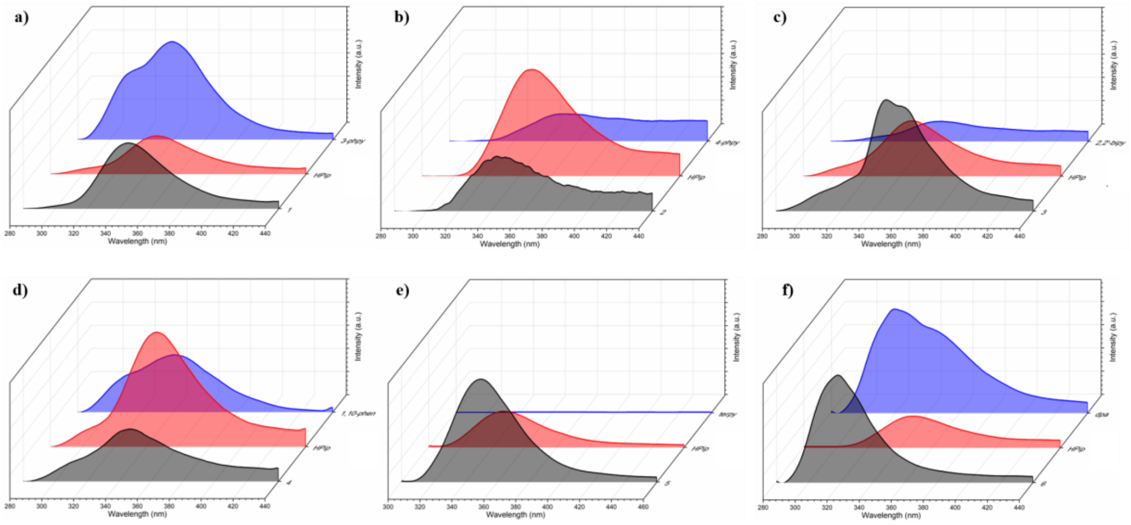

Figure S25. Comparative emission spectra of the complexes with their corresponding HPip and dPy ligands at the emission maxima of the complex. (a) **1**; (b) **2**; (c) **3**; (d) **4**; (e) **5**; (f) **6**. Color codes: blue (dPy), red (HPip) and black (**1-6**).

Table S1. UV-Vis data of the L-tyr, HPip and dPy ligands

Sample	$\lambda_{max-Abs}$ (log (ε))
L-Tyr	193(*4.62*); 223(*3.87*); 275(*3.11*); 282(*3.03*)
HPip	204(*4.55*); 255(*4.15*); 292(*3.57*)
3-phpy	204(*4.44*); 245(*4.23*); 275(*3.90*)
4-phpy	201(*4.02*); 257(*3.68*)
2,2'-bipy	202(*4.00*); 231(*3.84*); 243(*3.79*); 280(*3.94*)
1,10-phen	202(*4.38*); 226(*4.60*); 264(*4.44*); 308(*3.10*);
terpy	203(*4.41*); 231(*4.30*); 277(*4.26*);283(*4.25*); 333(3.20)
dpa	205(*3.98*); 261(*3.95*); 351(*2.50*)

All the wavelengths are given in nm. ε values are given in $M^{-1}\cdot cm^{-1}$.

HOMO and LUMO analysis

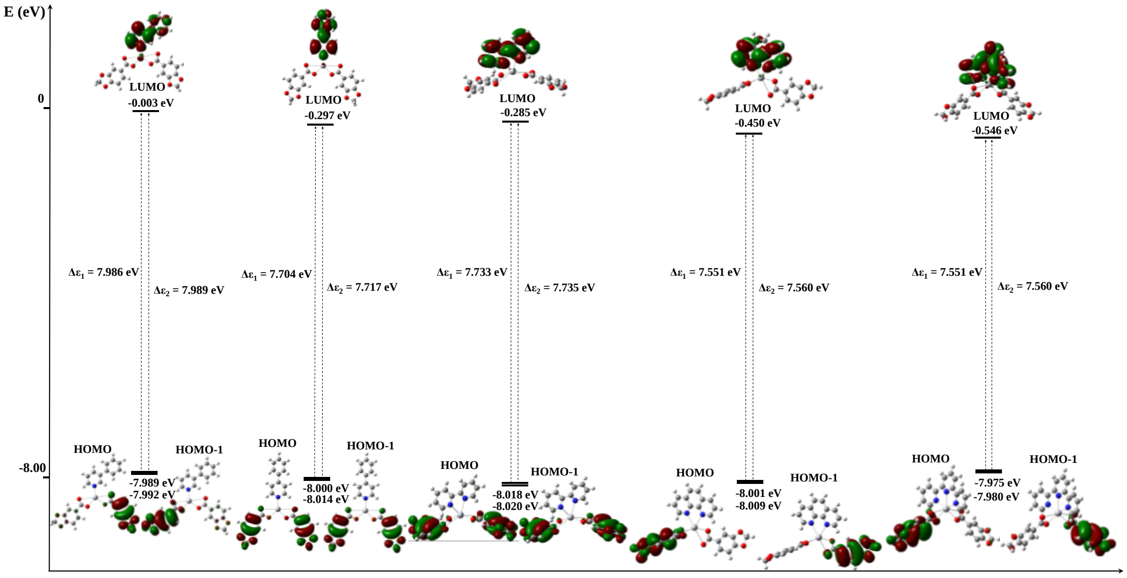

Figure S26. Calculated HOMO and LUMO of complexes **1-5** with their corresponding energy values and band gap.

2D color filled mapping of TDM

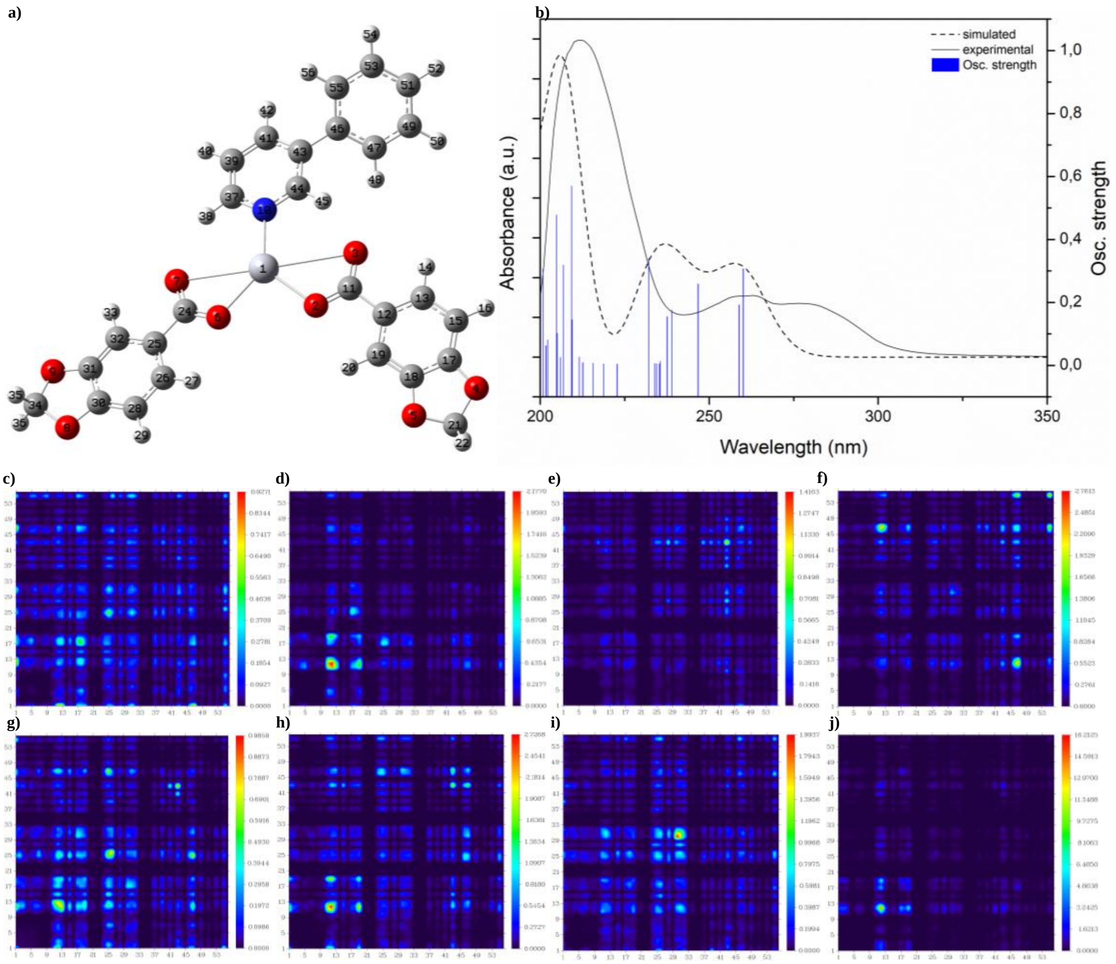

Figure S27. (a) Molecular structure with atom labelling and (b) experimental (solid line) and calculated (dashed line) UV-Vis spectra of compound **1**. 2D color filled mapping of TDM for transitions (c) 1; (d) 2; (e) 3; (f) 10; (g) 17; (h) 20, (i) 24 and (j) 35.

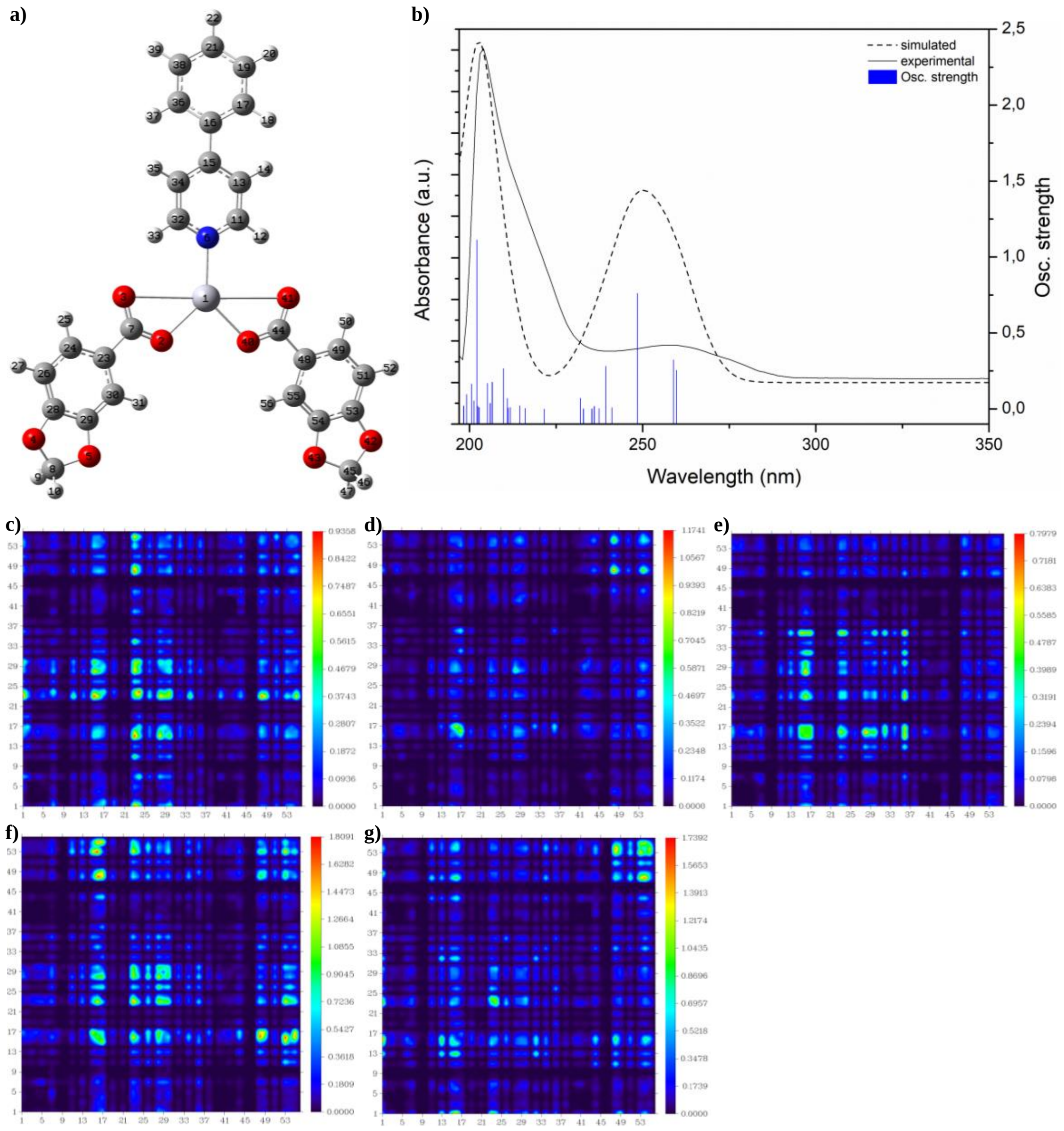

Figure 28. (a) Molecular structure with atom labelling and (b) experimental (solid line) and calculated (dashed line) UV-Vis spectra of compound **2**. 2D color filled mapping of TDM for transitions (c) 1; (d) 2; (e) 3; (f) 24 and (g) 32.

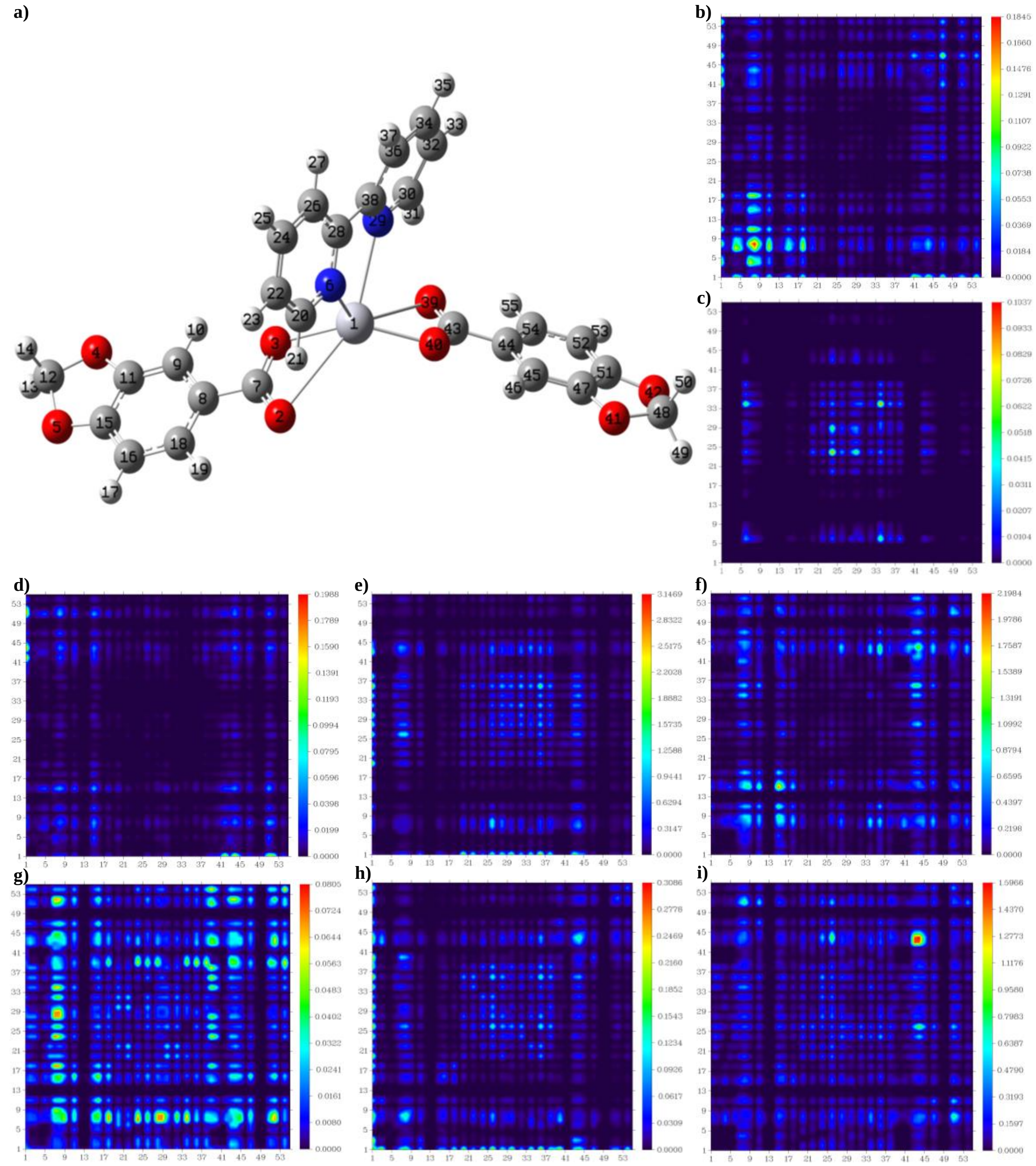

Figure S29. (a) Molecular structure and atom labelling of compound **3**. 2D color filled mapping of TDM for transitions (b) 1; (c) 3; (d) 5; (e) 12; (f) 25; (g) 30; (h) 33; and (i) 35.

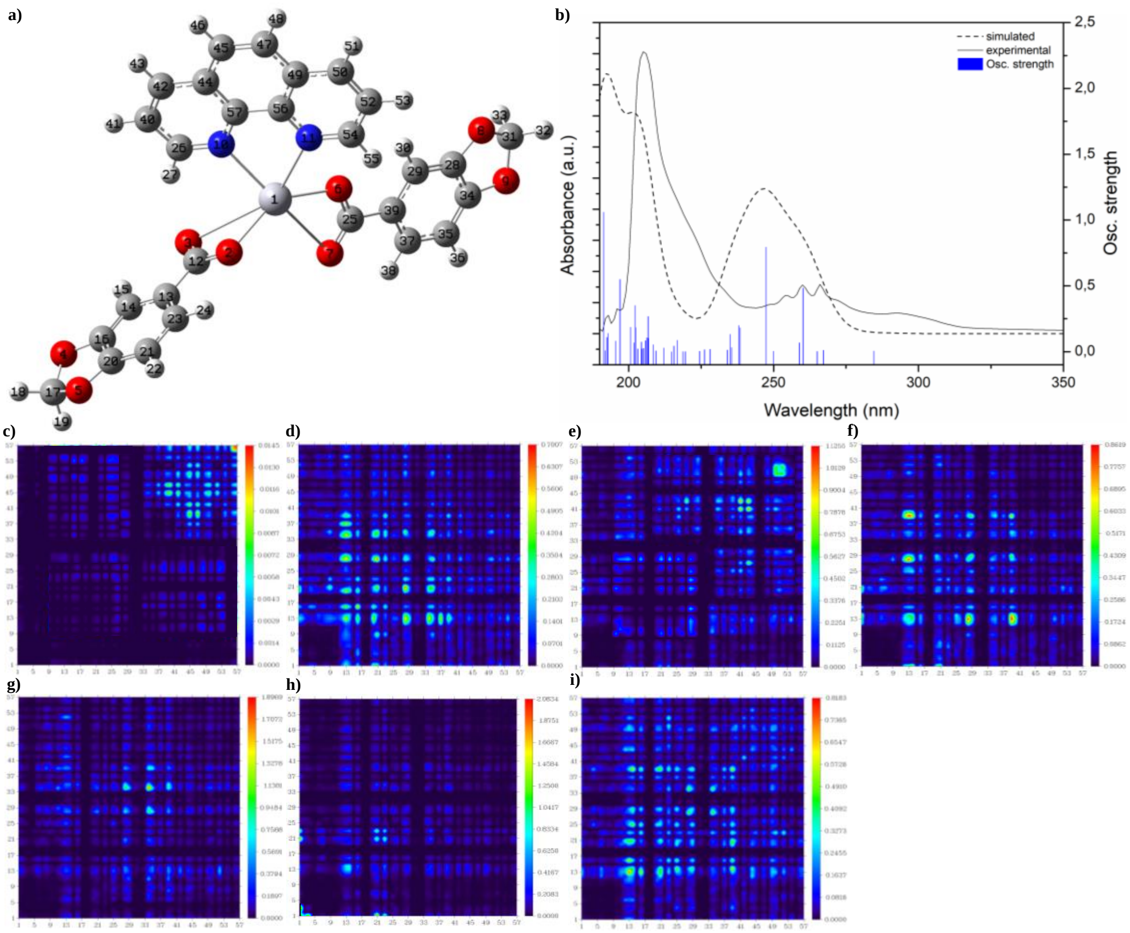

Figure S30. (a) Molecular structure with atom labelling and (b) experimental (solid line) and calculated (dashed line) UV-Vis spectra of compound **4**. 2D color filled mapping of TDM for transitions (c) 1; (d) 4; (e) 7; (f) 8; (g) 32; (h) 36 and (i) 41.

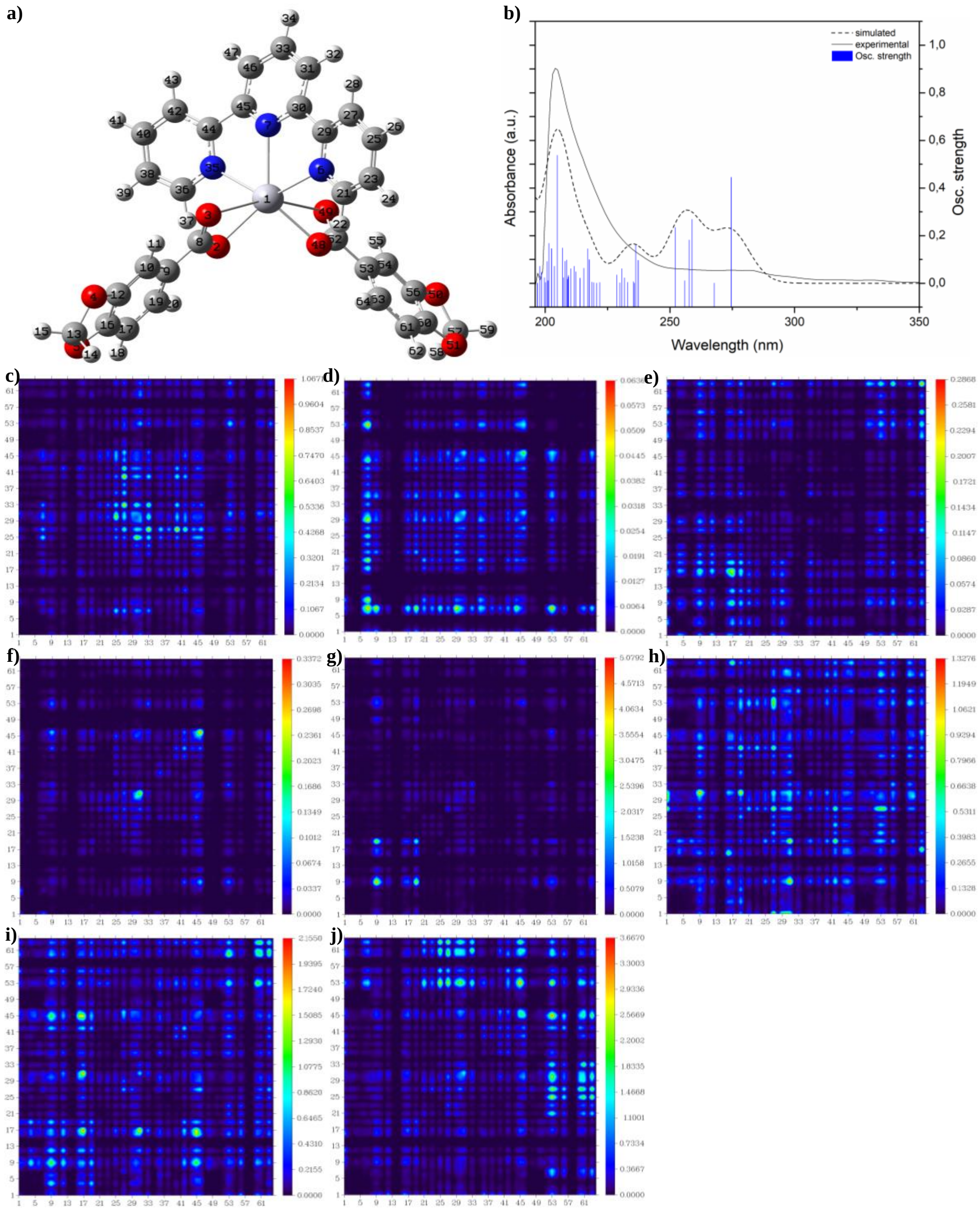

Figure S31. (a) Molecular structure with atom labelling and (b) experimental (solid line) and calculated (dashed line) UV-Vis spectra of compound **5**. 2D color filled mapping of TDM for transitions (c) 1; (d) 2; (e) 3; (f) 6; (g) 8; (h) 34; (i) 51; and (j) 52.

Table S2. Computational details of electronic transitions calculation.

Complex	Δr	$\lambda_{max-abs}$ Exp	$\lambda_{max-abs}$ Sim	Transition (character)	TS	Transition contributors
**1**	1.10	278	260.05	LC (LE)	1	Pip→Pip
	7.47		258.81	IL (CT)	2	Pip→Pip
	1.06	258	246.69	LC (LE)	3	3-phpy→3-phpy
	1.37		232.07	LC (LE)	10	3-phpy→3-phpy
	5.74	212	209.25	LMCT	17	Pip→Hg
	5.28		204.96	MLCT	20	Hg→Pip
	5.11		200.79	MLCT	24	Hg→Pip
	1.56		188.90	LC (LE)	35	Pip→Pip
**2**	1.19	293	259.75	LC (LE)	1	Pip→Pip
	1.14		258.88	LC (LE)	2	Pip→Pip
	1.28	259	248.42	LC (LE)	3	4-phpy→4-phpy
	3.21	205	202.08	MLCT+LMCT (CT)	24	Hg+Pip→Pip and Pip→Hg
	6.11		188.99	MLCT (CT)	32	4-phpy+Pip+Hg→4-phpy
**3**	1.12	287	260.51	LC (LE)	1	Pip→Pip
	0.47	260	257.11	LC (LE)	3	2,2’-bipy→2,2’-bipy
	1.67		238.92	LC (LE)	5	Pip→Pip
	0.82	215	224.26	LC (LE)	12	2,2’-bipy→2,2’-bipy
	2.91	203	202.48	LMCT+ MLCT(CT)	25	Pip→Hg and Hg→Pip
	2.13		195.78	LMCT (CT)	30	Pip+2,2’-bipy→Hg
	2.24		192.51	LMCT (CT) + IL (LE)	33	Pip→Hg and 2,2’-bipy→2,2’-bipy
	2.11		189.19	MLCT (CT)	35	Hg+Pip+2,2’-bipy→2,2’-bipy
**4**	1.75	294	284.61	LC (LE)	1	1,10-phen→1,10-phen
	1.69	266	260.12	LC (LE)	4	Pip→Pip
	1.33		247.41	LC (LE)	7	1,10-phen→1,10-phen
	1.31		238.44	LC (LE)	8	Pip→Pip
	7.35	206	202.42	MLCT (CT)	32	Hg→Pip
	3.52		197.03	LMCT (CT)	36	Terpy+Pip→Hg
	2.42		191.40	LMCT (CT)	41	Pip→Hg
**5**	0.85	282	274.59	LC (LE)	1	Terpy→terpy
	4.19	253	258.91	MLCT (CT)	2	Hg→terpy
	0.50		257.71	LC (LE)	3	Pip→Pip
	1.61		252.10	LC (LE)	6	terpy→terpy
	1.74		236.27	LC (LE))	8	Pip→Pip
	5.77	205	204.82	LMCT (CT)	34	Pip→Hg+terpy→terpy
	7.18		189.15	LC (LE) + LMCT	51	Pip→Pip and Pip→Hg
	7.49		188.55	LC (LE)	52	Pip→Pip

All the wavelengths are given in nm. TS = transition state; LC = ligand centered; IL = interligand; CT = charge transfer; LMCT = ligand-to-metal charge transfer; MLCT = metal-to-ligand charge transfer

NTOs analysis

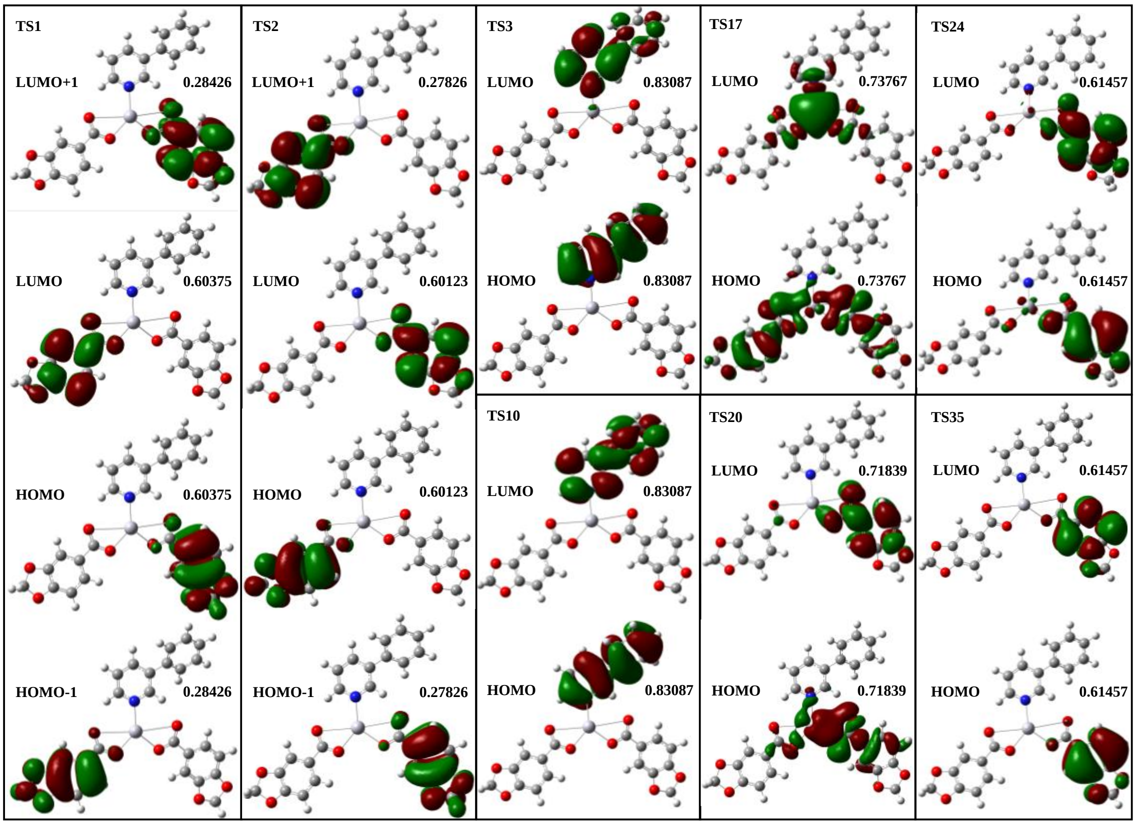

Figure S32. NTOs analysis of complex **1**.

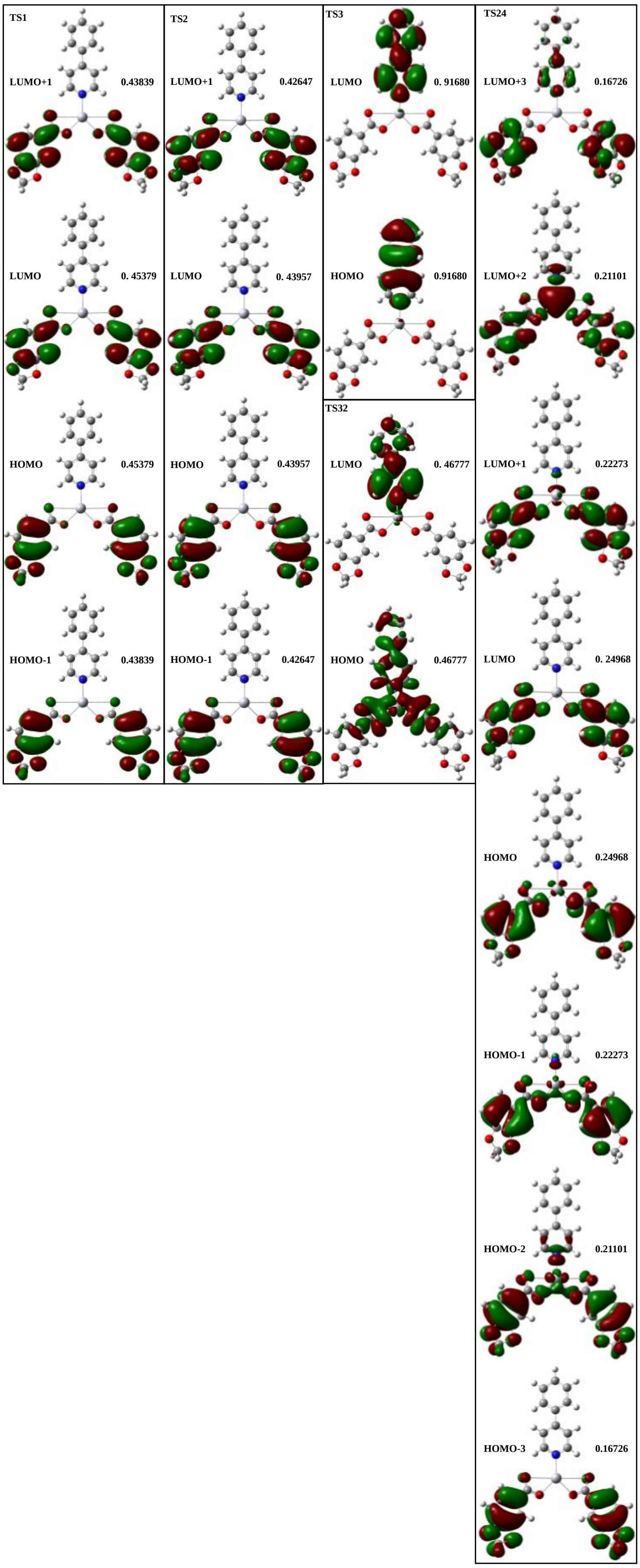

Figure S33. NTOs analysis of complex **2**.

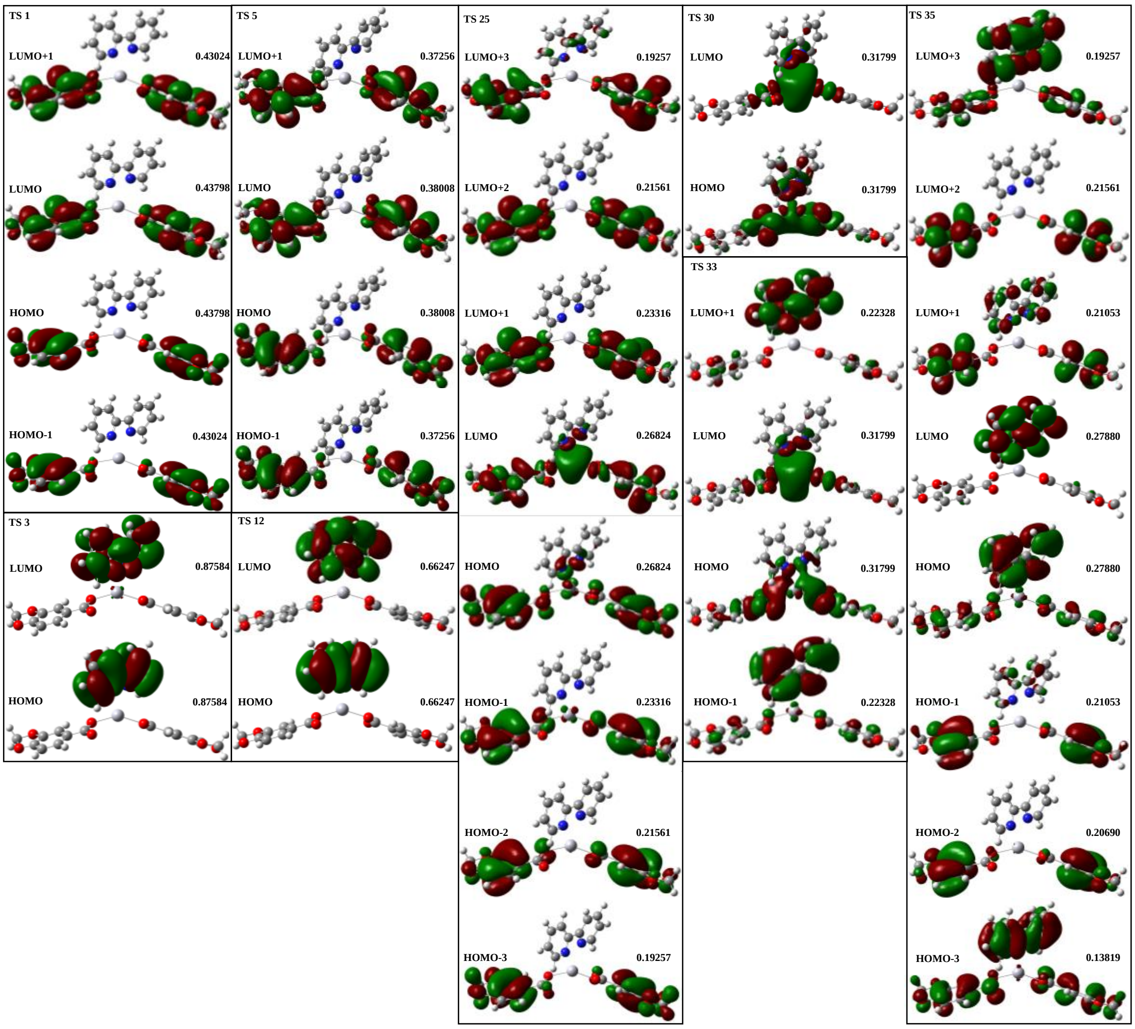

Figure S34. NTOs analysis of complex **3**.

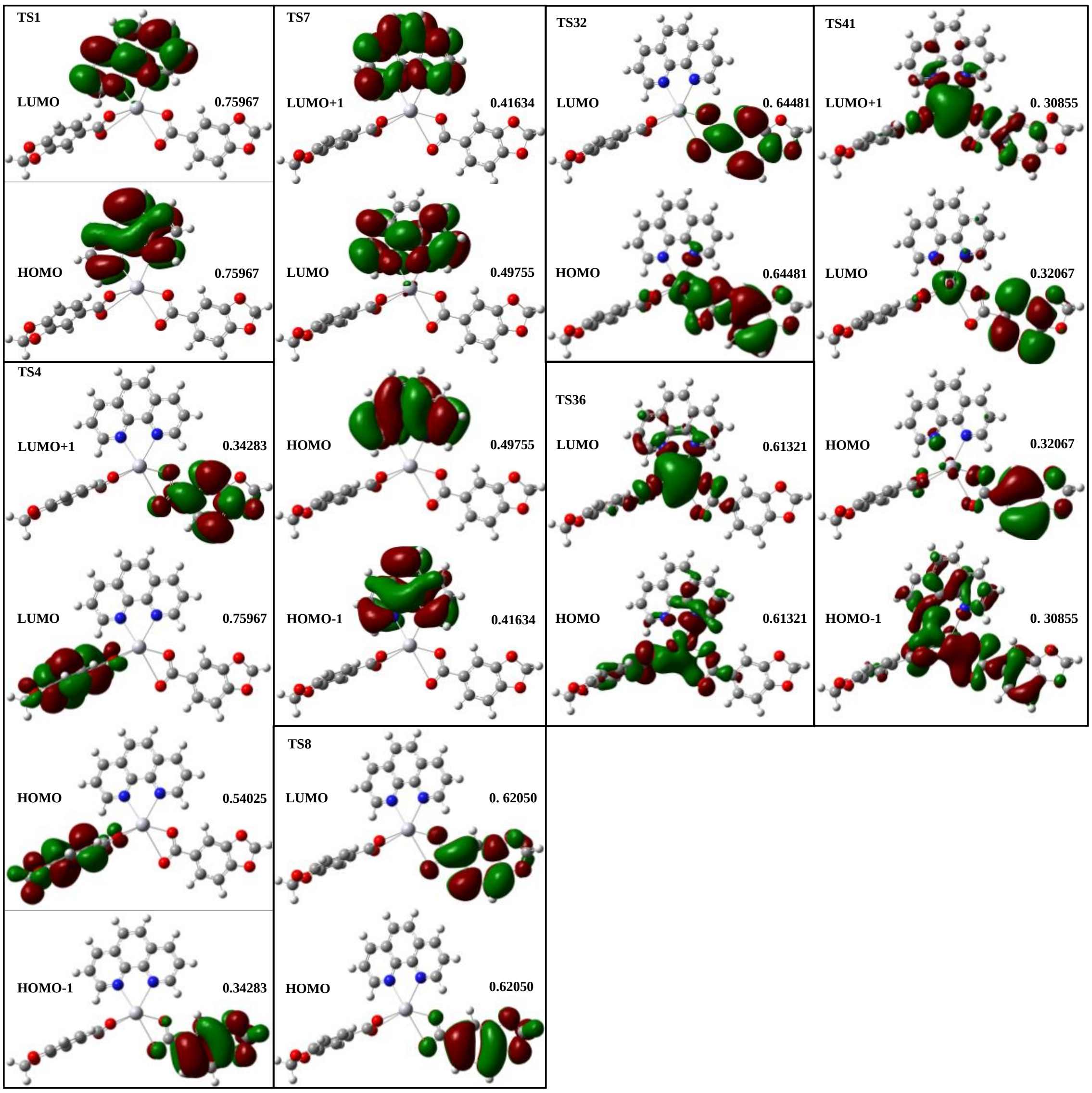

Figure S35. NTOs analysis of complex **4**.

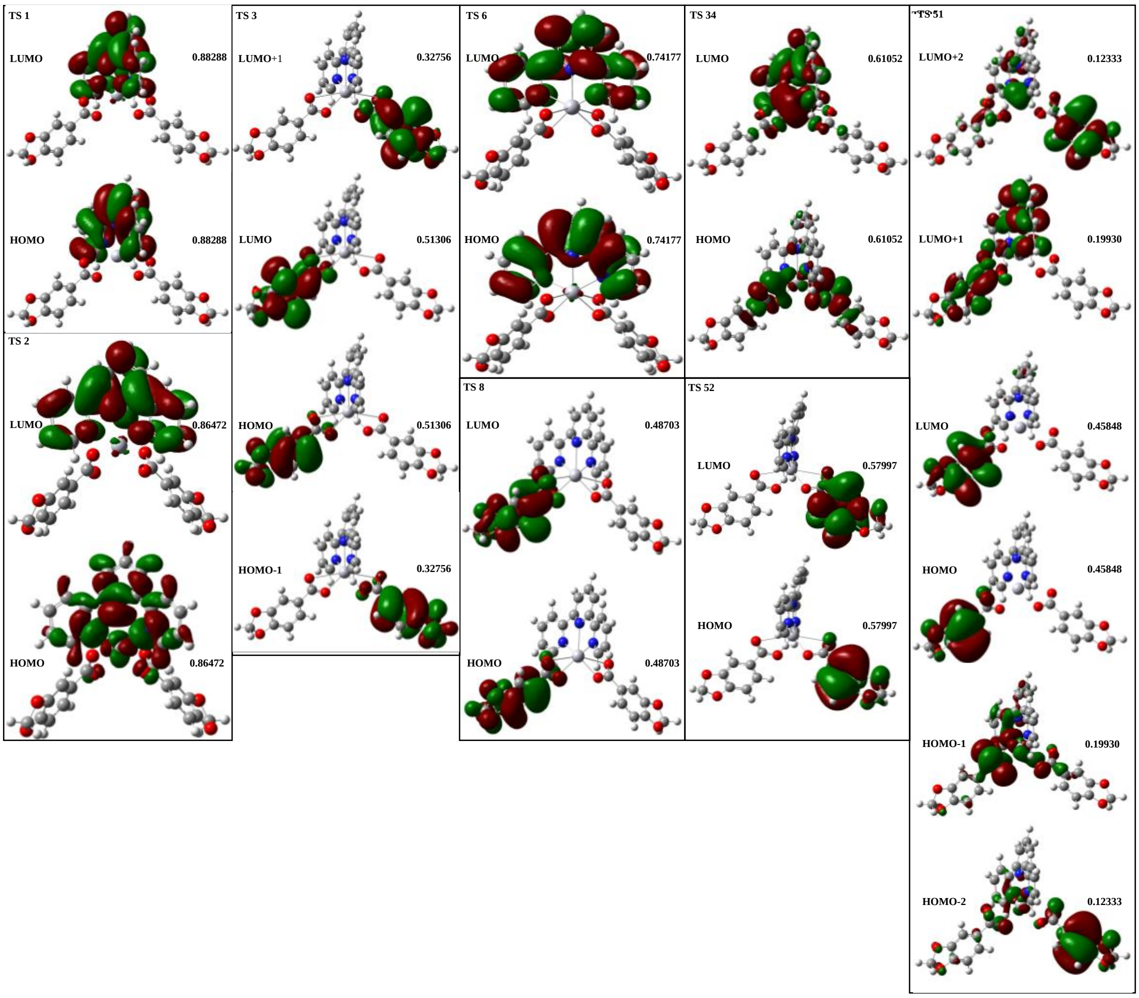

Figure S36. NTOs analysis of complex **5**.

Table S3. Cartesian coordinates from X-ray and optimized geometry of **1**.

Symbol (label)	X-Ray structure			Optimized geometry		
	X	Y	Z	X	Y	Z
Hg (1)	-0.229859	-0.496142	-0.314245	-0.229742	-0.496296	-0.314991
O (2)	0.690355	1.599883	-0.324115	0.690089	1.599923	-0.324965
O (3)	2.718708	0.653197	-0.334066	2.718504	0.653376	-0.334634
O (4)	4.027888	6.814507	0.268193	4.027201	6.814664	0.268843
O (5)	1.745168	6.587777	0.221865	1.744517	6.587799	0.222268
O (6)	-2.363975	0.251236	-0.337189	-2.363992	0.251212	-0.337701
O (7)	-2.985053	-1.829651	0.195510	-2.984825	-1.829760	0.194913
O (8)	-8.618343	0.867315	0.140911	-8.618284	0.866893	0.142017
O (9)	-8.108697	-1.326825	0.580654	-8.108478	-1.327449	0.580608
N (10)	0.766123	-2.478634	0.082521	0.766335	-2.478538	0.082441
C (11)	1.968708	1.636251	-0.280020	1.968430	1.636373	-0.280607
C (12)	2.564796	3.015530	-0.141713	2.564400	3.015677	-0.142038
C (13)	3.945660	3.165488	-0.117075	3.945253	3.165722	-0.117247
H (14)	4.561798	2.280537	-0.206023	4.561452	2.280822	-0.206296
C (15)	4.548369	4.418503	0.025200	4.547871	4.418743	0.025343
H (16)	5.624113	4.534377	0.040272	5.623610	4.534675	0.040530
C (17)	3.706990	5.496260	0.147212	3.706413	5.496418	0.147533
C (18)	2.328120	5.354742	0.122185	2.327549	5.354813	0.122356
C (19)	1.724238	4.135351	-0.024544	1.723761	4.135417	-0.024707
H (20)	0.649286	4.023542	-0.049109	0.648814	4.023534	-0.049391
C (21)	2.800881	7.473014	0.583683	2.800137	7.472929	0.584605
H (22)	2.722446	8.389846	0.003976	2.721669	8.390055	0.005372
H (23)	2.762678	7.666706	1.660614	2.761863	7.666042	1.661642
C (24)	-3.234070	-0.644377	-0.052093	-3.233954	-0.644455	-0.052476
C (25)	-4.665001	-0.171059	-0.024414	-4.664927	-0.171253	-0.024436
C (26)	-4.983965	1.155906	-0.285048	-4.983995	1.155818	-0.284419
H (27)	-4.183698	1.847191	-0.511558	-4.183786	1.847229	-0.510757
C (28)	-6.305311	1.612317	-0.262878	-6.305351	1.612170	-0.261808
H (29)	-6.552796	2.646887	-0.460993	-6.552904	2.646829	-0.459382
C (30)	-7.274167	0.683430	0.024341	-7.274127	0.683112	0.025138
C (31)	-6.962621	-0.642049	0.286167	-6.962483	-0.642477	0.286277
C (32)	-5.674235	-1.101792	0.273374	-5.674077	-1.102158	0.273081
H (33)	-5.424861	-2.132724	0.483848	-5.424642	-2.133177	0.483074
C (34)	-9.173269	-0.445022	0.236265	-9.173169	-0.445490	0.236987
H (35)	-9.586610	-0.732786	-0.735724	-9.586868	-0.732822	-0.734976
H (36)	-9.928334	-0.462697	1.018965	-9.927958	-0.463516	1.019953
C (37)	0.031915	-3.565799	0.338134	0.032196	-3.565642	0.338481
H (38)	-1.042358	-3.425107	0.359772	-1.042087	-3.424991	0.360079
C (39)	0.629244	-4.791029	0.561553	0.629603	-4.790752	0.562386
H (40)	0.016656	-5.656952	0.771970	0.017053	-5.656624	0.773137
C (41)	2.010051	-4.884156	0.519654	2.010410	-4.883806	0.520534
H (42)	2.493928	-5.835188	0.707923	2.494357	-5.834735	0.709156
C (43)	2.776488	-3.751715	0.255051	2.776780	-3.751412	0.255507
C (44)	2.097709	-2.558612	0.039520	2.097938	-2.558444	0.039494
H (45)	2.617566	-1.631801	-0.180640	2.617726	-1.631674	-0.181015

C (46)	4.255465	-3.791966	0.192883	4.255767	-3.791608	0.193391
C (47)	5.016746	-2.748562	0.719820	5.016967	-2.748022	0.720079
H (48)	4.525584	-1.910249	1.200528	4.525736	-1.909607	1.200545
C (49)	6.401446	-2.783782	0.652750	6.401675	-2.783178	0.653061
H (50)	6.979618	-1.968858	1.071400	6.979795	-1.968110	1.071512
C (51)	7.044243	-3.861353	0.058069	7.044548	-3.860870	0.058687
H (52)	8.125961	-3.887932	0.005040	8.126274	-3.887404	0.005701
C (53)	6.294427	-4.904492	-0.468858	6.294809	-4.904194	-0.467988
H (54)	6.788476	-5.745271	-0.940776	6.788926	-5.745071	-0.939669
C (55)	4.909642	-4.870531	-0.402040	4.910019	-4.870295	-0.401226
H (56)	4.333022	-5.680206	-0.834733	4.333455	-5.680119	-0.833720

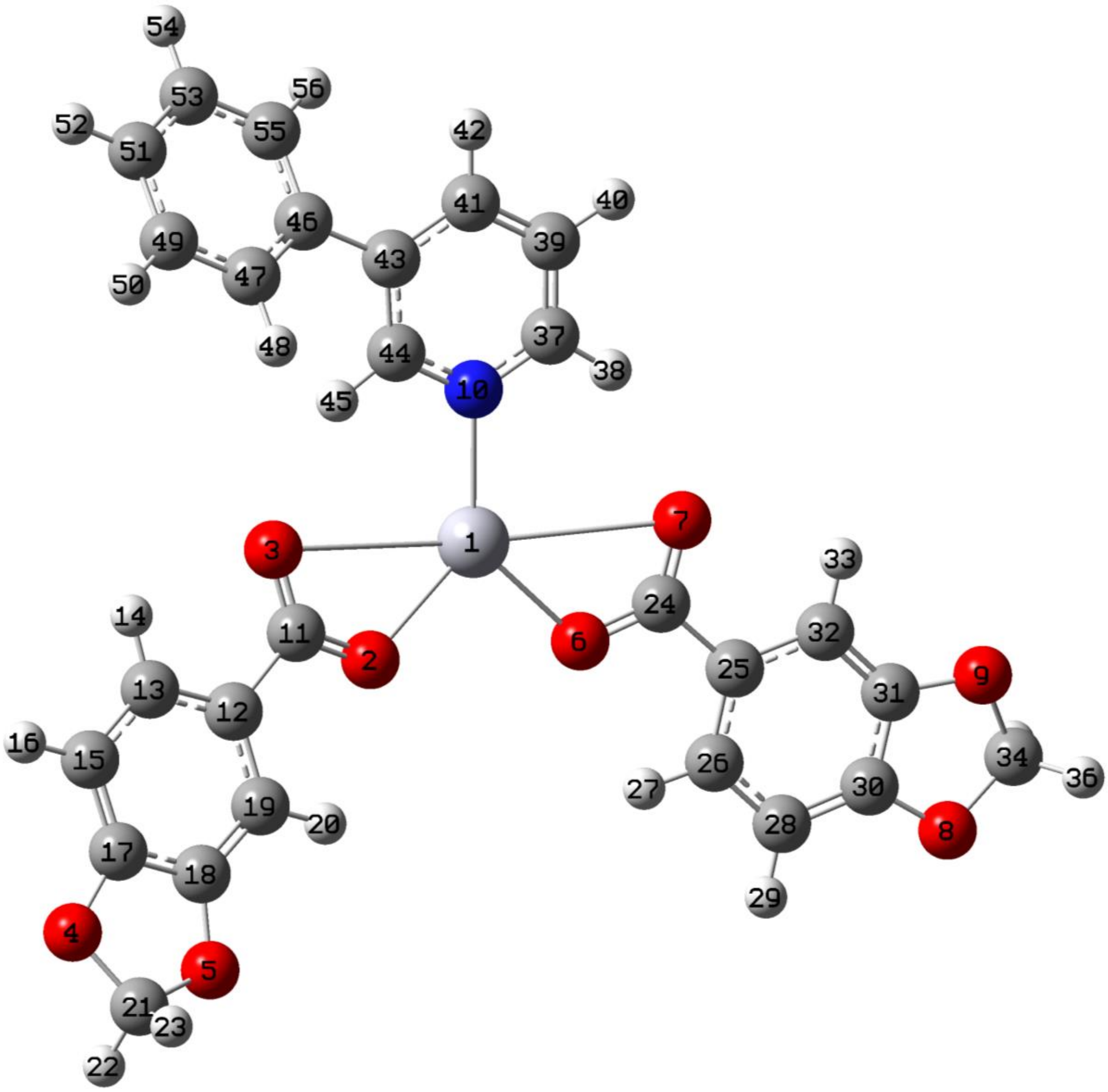

Figure S37. Optimized geometry of **1** in MeOH solution with labelling scheme.

Table S4. Cartesian coordinates from X-ray and optimized geometry of **2**.

Symbol (label)	X-Ray structure			Optimized geometry		
	X	Y	Z	X	Y	Z
Hg (1)	0.000000	0.000000	0.269141	0.000000	0.000000	0.274220
O (2)	0.111657	1.631584	-1.318402	0.104594	1.628775	-1.314275
O (3)	0.014441	3.165971	0.309426	0.000000	3.167206	0.309272
O (4)	-0.168363	6.666235	-4.957268	-0.368667	6.637904	-4.967672
O (5)	-0.071791	4.461623	-5.588056	-0.238885	4.432582	-5.590414
N (6)	0.000000	0.000000	2.512262	0.000000	0.000000	2.517286
C (7)	0.049662	2.833525	-0.880800	0.022917	2.830953	-0.880153
C (8)	0.102378	5.795763	-6.056235	-0.106440	5.767794	-6.069059
H (9)	1.138247	5.936962	-6.380985	0.917368	5.930347	-6.420835
H (10)	-0.604506	5.995278	-6.858501	-0.837798	5.947303	-6.853948
C (11)	0.011949	-1.155402	3.185851	0.020295	-1.154985	3.190870
H (12)	0.025081	-2.058159	2.585846	0.039497	-2.057662	2.590940
C (13)	0.014193	-1.191626	4.564188	0.022988	-1.191190	4.569206
H (14)	0.045990	-2.150246	5.064612	0.061850	-2.149568	5.069566
C (15)	0.000000	0.000000	5.289127	0.000000	0.000000	5.294173
C (16)	0.000000	0.000000	6.768858	0.000000	0.000000	6.773906
C (17)	-0.715976	-0.965849	7.476984	-0.707321	-0.971847	7.482072
H (18)	-1.293167	-1.711718	6.942997	-1.277760	-1.722929	6.948159
C (19)	-0.718869	-0.962714	8.863360	-0.710319	-0.968666	8.868445
H (20)	-1.287504	-1.711917	9.400707	-1.272184	-1.722949	9.405789
C (21)	0.000000	0.000000	9.559913	0.000000	0.000000	9.564997
H (22)	0.000000	0.000000	10.643239	0.000000	0.000000	10.648314
C (23)	0.009600	3.898193	-1.949644	-0.060814	3.889656	-1.952382
C (24)	-0.051840	5.235745	-1.579741	-0.142480	5.227434	-1.587271
H (25)	-0.059159	5.478059	-0.525288	-0.131409	5.474848	-0.534050
C (26)	-0.105215	6.261036	-2.528086	-0.239726	6.246523	-2.538858
H (27)	-0.159193	7.302256	-2.238443	-0.309665	7.287773	-2.252791
C (28)	-0.089889	5.881106	-3.847115	-0.246988	5.860342	-3.856171
C (29)	-0.028250	4.548515	-4.224115	-0.165240	4.527493	-4.228313
C (30)	0.020446	3.534027	-3.306635	-0.073429	3.518986	-3.307587
H (31)	0.059385	2.493877	-3.598485	-0.019383	2.478460	-3.595587
C (32)	-0.011949	1.155402	3.185851	-0.020295	1.154985	3.190870
H (33)	-0.025081	2.058159	2.585846	-0.039497	2.057662	2.590940
C (34)	-0.014193	1.191626	4.564188	-0.022988	1.191190	4.569206
H (35)	-0.045990	2.150246	5.064612	-0.061850	2.149568	5.069566
C (36)	0.715976	0.965849	7.476984	0.707321	0.971847	7.482072
H (37)	1.293167	1.711718	6.942997	1.277760	1.722929	6.948159
C (38)	0.718869	0.962714	8.863360	0.710319	0.968666	8.868445
H (39)	1.287504	1.711917	9.400707	1.272184	1.722949	9.405789
O (40)	-0.111657	-1.631584	-1.318402	-0.104594	-1.628775	-1.314275
O (41)	-0.014441	-3.165971	0.309426	0.000000	-3.167206	0.309272
O (42)	0.168363	-6.666235	-4.957268	0.368667	-6.637904	-4.967672
O (43)	0.071791	-4.461623	-5.588056	0.238885	-4.432582	-5.590414
C (44)	-0.049662	-2.833525	-0.880800	-0.022917	-2.830953	-0.880153
C (45)	-0.102378	-5.795763	-6.056235	0.106440	-5.767794	-6.069059

H (46)	-1.138247	-5.936962	-6.380985	-0.917368	-5.930347	-6.420835
H (47)	0.604506	-5.995278	-6.858501	0.837798	-5.947303	-6.853948
C (48)	-0.009600	-3.898193	-1.949644	0.060814	-3.889656	-1.952382
C (49)	0.051840	-5.235745	-1.579741	0.142480	-5.227434	-1.587271
H (50)	0.059159	-5.478059	-0.525288	0.131409	-5.474848	-0.534050
C (51)	0.105215	-6.261036	-2.528086	0.239726	-6.246523	-2.538858
H (52)	0.159193	-7.302256	-2.238443	0.309665	-7.287773	-2.252791
C (53)	0.089889	-5.881106	-3.847115	0.246988	-5.860342	-3.856171
C (54)	0.028250	-4.548515	-4.224115	0.165240	-4.527493	-4.228313
C (55)	-0.020446	-3.534027	-3.306635	0.073429	-3.518986	-3.307587
H (56)	-0.059385	-2.493877	-3.598485	0.019383	-2.478460	-3.595587

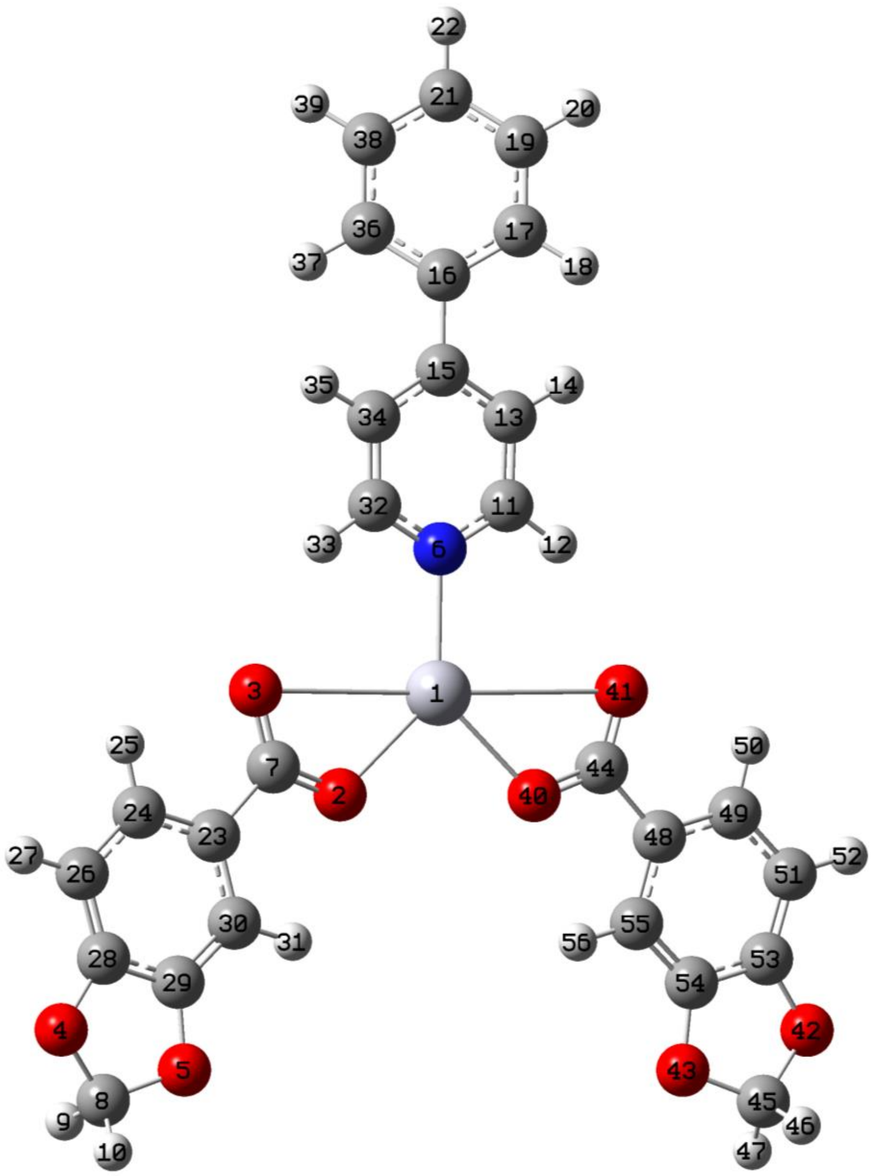

Figure S38. Optimized geometry of **2** in MeOH solution with labelling scheme.

Table S5. Cartesian coordinates from X-ray and optimized geometry of **3**.

Symbol (label)	X-Ray structure			Optimized geometry		
	X	Y	Z	X	Y	Z
Hg (1)	0.000000	0.000000	0.007249	0.000000	0.000000	0.074801
O (2)	-2.279430	-1.926389	-1.114710	-1.858182	2.263904	-1.044737
O (3)	-2.109819	0.248609	-0.627391	0.318617	2.089384	-0.564606
O (4)	-6.776636	2.067087	-1.683365	2.213221	6.651579	-1.863609
O (5)	-8.047063	0.322165	-2.443563	0.471051	7.942603	-2.617115
N (6)	-0.765790	-1.145215	2.195786	-1.130421	0.789231	2.279568
C (7)	-2.747169	-0.788619	-1.031976	-0.715383	2.723335	-0.986949
C (8)	-4.181824	-0.530952	-1.416983	-0.440136	4.136905	-1.427866
C (9)	-4.721188	0.761408	-1.301468	0.867779	4.648135	-1.371248
H (10)	-4.116865	1.584559	-0.946261	1.686395	4.036498	-1.019180
C (11)	-6.032326	0.922121	-1.661100	1.049077	5.940096	-1.783231
C (12)	-8.115507	1.632692	-1.886048	1.819290	7.990689	-2.147857
H (13)	-8.616598	2.300647	-2.584051	2.458861	8.400742	-2.926336
H (14)	-8.635666	1.587062	-0.921409	1.860381	8.586737	-1.230746
C (15)	-6.804990	-0.132980	-2.126253	0.000000	6.721649	-2.242354
C (16)	-6.296555	-1.402352	-2.254125	-1.284675	6.242428	-2.310889
H (17)	-6.900702	-2.220862	-2.623617	-2.099333	6.854472	-2.674825
C (18)	-4.960764	-1.582603	-1.884626	-1.486343	4.925080	-1.889473
H (19)	-4.511406	-2.564519	-1.959209	-2.480025	4.498110	-1.918348
C (20)	-1.663226	-2.126586	2.142272	-2.084226	1.715709	2.227111
H (21)	-1.837380	-2.555994	1.161283	-2.511223	1.898328	1.247108
C (22)	-2.353127	-2.567143	3.259776	-2.498920	2.423971	3.342577
H (23)	-3.072743	-3.370670	3.173358	-3.280278	3.166947	3.255640
C ()24	-2.105742	-1.942019	4.470705	-1.876687	2.164084	4.551820
H (25)	-2.637041	-2.240478	5.366320	-2.155488	2.707984	5.445688
C (26)	-1.176158	-0.916380	4.524184	-0.878708	1.205539	4.604934
H (27)	-0.993507	-0.400857	5.457142	-0.364052	1.011498	5.535644
C (28)	-0.509435	-0.545549	3.360346	-0.532107	0.523769	3.442907
N (29)	0.765790	1.145215	2.195786	1.130421	-0.789231	2.279568
C (30)	1.663226	2.126586	2.142272	2.084226	-1.715709	2.227111
H (31)	1.837380	2.555994	1.161283	2.511223	-1.898328	1.247108
C (32)	2.353127	2.567143	3.259776	2.498920	-2.423971	3.342577
H (33)	3.072743	3.370670	3.173358	3.280278	-3.166947	3.255640
C (34)	2.105742	1.942019	4.470705	1.876687	-2.164084	4.551820
H (35)	2.637041	2.240478	5.366320	2.155488	-2.707984	5.445688
C (36)	1.176158	0.916380	4.524184	0.878708	-1.205539	4.604934
H (37)	0.993507	0.400857	5.457142	0.364052	-1.011498	5.535644
C (38)	0.509435	0.545549	3.360346	0.532107	-0.523769	3.442907
O (39)	2.279430	1.926389	-1.114710	1.858182	-2.263904	-1.044737
O (40)	2.109819	-0.248609	-0.627391	-0.318617	-2.089384	-0.564606
O (41)	6.776636	-2.067087	-1.683365	-2.213221	-6.651579	-1.863609
O (42)	8.047063	-0.322165	-2.443563	-0.471051	-7.942603	-2.617115
C (43)	2.747169	0.788619	-1.031976	0.715383	-2.723335	-0.986949
C (44)	4.181824	0.530952	-1.416983	0.440136	-4.136905	-1.427866
C (45)	4.721188	-0.761408	-1.301468	-0.867779	-4.648135	-1.371248

H (46)	4.116865	-1.584559	-0.946261	-1.686395	-4.036498	-1.019180
C (47)	6.032326	-0.922121	-1.661100	-1.049077	-5.940096	-1.783231
C (48)	8.115507	-1.632692	-1.886048	-1.819290	-7.990689	-2.147857
H (49)	8.616598	-2.300647	-2.584051	-2.458861	-8.400742	-2.926336
H (50)	8.635666	-1.587062	-0.921409	-1.860381	-8.586737	-1.230746
C (51)	6.804990	0.132980	-2.126253	0.000000	-6.721649	-2.242354
C (52)	6.296555	1.402352	-2.254125	1.284675	-6.242428	-2.310889
H (53)	6.900702	2.220862	-2.623617	2.099333	-6.854472	-2.674825
C (54)	4.960764	1.582603	-1.884626	1.486343	-4.925080	-1.889473
H (55)	4.511406	2.564519	-1.959209	2.480025	-4.498110	-1.918348

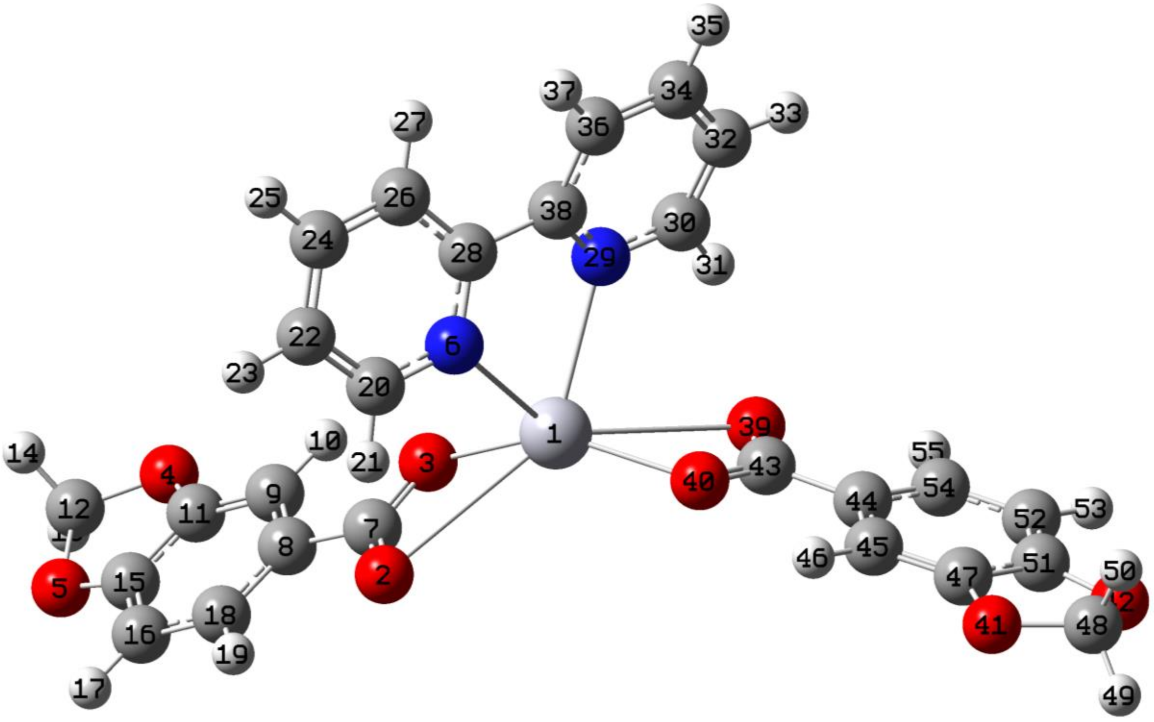

Figure S39. Optimized geometry of **3** in MeOH solution with labelling scheme.

Table S6. Cartesian coordinates from X-ray and optimized geometry of **4**.

Symbol (label)	X-Ray structure			Optimized geometry		
	X	Y	Z	X	Y	Z
Hg (1)	-0.096270	0.167847	-0.006718	0.069780	0.082468	0.065592
O (2)	1.916529	-0.596841	-0.682874	-2.006993	-0.557928	0.654778
O (3)	2.463542	-0.972842	1.490140	-2.437356	-1.179029	-1.449403
O (4)	7.328010	-2.766258	1.046842	-7.358485	-2.716840	-1.070892
O (5)	7.823625	-2.867790	-1.247668	-7.969914	-2.520668	1.132679
O (6)	-2.205678	-0.552342	0.031893	2.183285	-0.631675	0.043019
O (7)	-1.424373	-2.684963	0.033801	1.435489	-2.739317	0.042455
O (8)	-7.319861	-1.162826	0.278934	7.262556	-1.174809	-0.245199
O (9)	-7.752321	-3.470148	0.373263	7.718591	-3.421792	-0.333592
N (10)	0.891739	2.184001	0.964358	-0.910807	2.195526	-0.835584
N (11)	-1.099115	2.217729	-0.963273	1.319975	2.228780	0.783284
C (12)	2.737351	-0.990800	0.255668	-2.757574	-1.037060	-0.264291
C (13)	4.074086	-1.480070	-0.208497	-4.142958	-1.431754	0.181888
C (14)	5.018138	-1.880000	0.760367	-5.045909	-1.917140	-0.778358
H (15)	4.778410	-1.830755	1.813759	-4.750921	-2.003306	-1.815152
C (16)	6.233479	-2.325121	0.298194	-6.295259	-2.263144	-0.340821
C (17)	8.360847	-3.163074	0.086415	-8.329217	-3.123779	-0.110819
H (18)	9.256487	-2.568846	0.251675	-9.311963	-2.769517	-0.413956
H (19)	8.542085	-4.232927	0.171412	-8.303404	-4.212846	-0.003669
C (20)	6.531353	-2.385023	-1.060923	-6.669613	-2.142755	0.988688
C (21)	5.624040	-1.998702	-2.025618	-5.806429	-1.667644	1.944284
H (22)	5.863470	-2.049431	-3.079365	-6.104449	-1.571327	2.980046
C (23)	4.377177	-1.539732	-1.572824	-4.525151	-1.312452	1.512492
H (24)	3.626187	-1.223743	-2.285046	-3.806073	-0.936092	2.227125
C (25)	-2.371602	-1.854375	0.059329	2.341822	-1.904784	0.012865
C (26)	1.864672	2.145290	1.876396	-1.983428	2.172248	-1.601429
H (27)	2.203614	1.157824	2.168980	-2.364494	1.189920	-1.860983
C (28)	-6.120319	-1.879046	0.240326	6.090963	-1.879432	-0.218384
C (29)	-4.841958	-1.380282	0.157402	4.811875	-1.403423	-0.117195
H (30)	-4.634056	-0.320343	0.116455	4.591329	-0.345865	-0.074102
C (31)	-8.392883	-2.149915	0.423464	8.265282	-2.127239	-0.586762
H (32)	-9.085867	-2.056351	-0.409075	9.140994	-1.975098	0.039980
H (33)	-8.874960	-2.022081	1.391205	8.505352	-2.039728	-1.651359
C (34)	-6.380665	-3.246025	0.294461	6.370939	-3.236497	-0.268062
C (35)	-5.369732	-4.184252	0.263964	5.378901	-4.183838	-0.215540
H (36)	-5.580454	-5.244454	0.306177	5.602587	-5.241973	-0.249712
C (37)	-4.057438	-3.694344	0.180259	4.066838	-3.712491	-0.118040
H (38)	-3.222581	-4.383512	0.157098	3.244654	-4.414938	-0.082978
C (39)	-3.791772	-2.321929	0.129849	3.778573	-2.354569	-0.071320
C (40)	2.425823	3.319563	2.408376	-2.608028	3.336483	-2.061725
H (41)	3.211745	3.249360	3.148375	-3.486649	3.262695	-2.688081
C (42)	1.958848	4.543913	1.974065	-2.083317	4.550226	-1.703335
H (43)	2.370385	5.466321	2.368200	-2.537454	5.474860	-2.040251
C (44)	0.933640	4.600286	1.001824	-0.939745	4.599082	-0.887610
C (45)	0.404783	5.843775	0.506920	-0.341097	5.838158	-0.485545

H (46)	0.813420	6.768533	0.898044	-0.794028	6.762649	-0.823667
C (47)	-0.584484	5.860317	-0.424635	0.761756	5.854333	0.290712
H (48)	-0.984172	6.798179	-0.793206	1.215432	6.791905	0.589560
C (49)	-1.125034	4.633567	-0.946617	1.360059	4.631907	0.739666
C (50)	-2.155511	4.605509	-1.914659	2.512046	4.609247	1.545209
H (51)	-2.562261	5.538854	-2.287212	2.972380	5.544038	1.843643
C (52)	-2.635118	3.394081	-2.372489	3.039806	3.407424	1.939385
H (53)	-3.425790	3.345397	-3.109124	3.927759	3.354698	2.554818
C (54)	-2.077116	2.206646	-1.867220	2.403005	2.229480	1.530815
H (55)	-2.426888	1.234011	-2.187591	2.788761	1.256144	1.812865
C (56)	-0.621923	3.396217	-0.488129	0.796161	3.397377	0.376289
C (57)	0.422763	3.377892	0.513407	-0.382597	3.379144	-0.469077

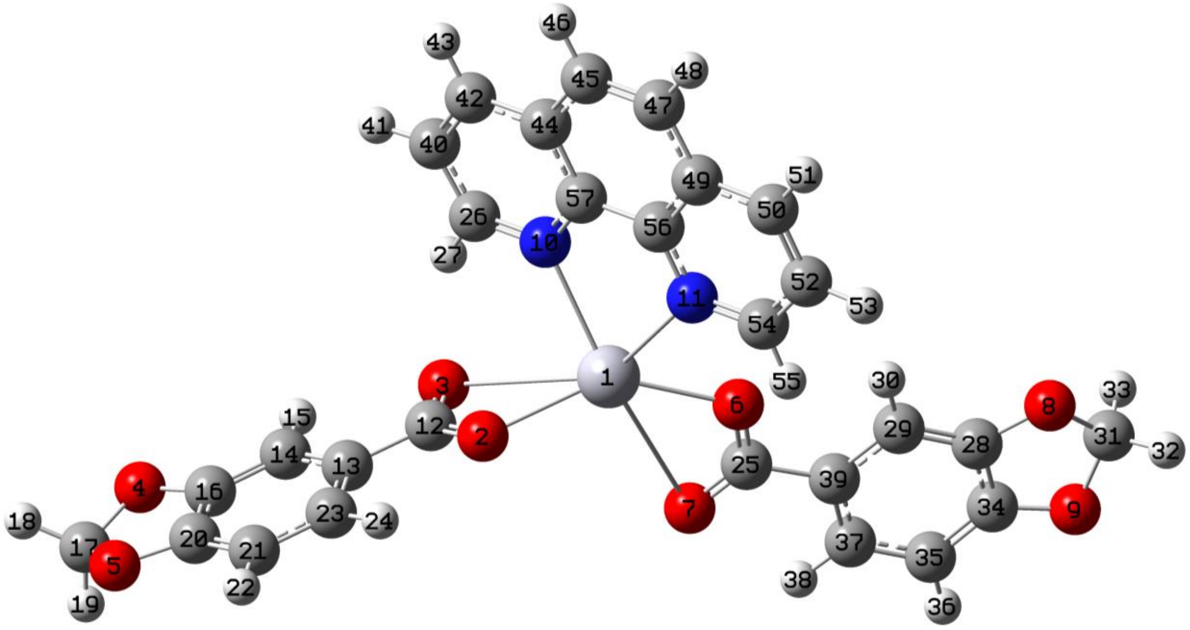

Figure S40. Optimized geometry of **4** in MeOH solution with labelling scheme.

Table S7. Cartesian coordinates from X-ray and optimized structure of **5**.

Symbol (label)	X-Ray structure			Optimized geometry		
	**X**	**Y**	**Z**	**X**	**Y**	**Z**
Hg(1)	0.000000	0.000000	0.648588	0.038391	0.638626	-0.017461
O (2)	0.417963	1.629994	-0.976114	-1.600735	-0.993998	-0.412586
O (3)	-1.137153	2.776933	0.140023	-2.755704	0.171322	1.099555
O (4)	-1.519615	7.081313	-2.685404	-7.132042	-2.54327	1.432652
O (5)	-0.042475	6.893154	-4.430655	-6.953829	-4.318371	-0.00969
N (6)	-2.080437	-0.847743	1.673820	0.811779	1.688198	2.138843
N (7)	0.000000	0.000000	3.181545	0.040409	3.172081	0.000483
C (8)	-0.327733	2.645483	-0.790542	-2.626835	-0.775387	0.308428
C (9)	-0.197241	3.757989	-1.803768	-3.758922	-1.766086	0.171992
C (10)	-1.008579	4.896029	-1.665217	-4.911242	-1.585802	0.954526
H (11)	-1.701097	4.976911	-0.838650	-4.987693	-0.745033	1.630208
C (12)	-0.879265	5.874745	-2.612688	-5.90973	-2.511355	0.819238
C (13)	-1.195248	7.599431	-3.971794	-7.674101	-3.822297	1.118665
H (14)	-2.027308	7.416577	-4.659778	-7.526773	-4.499642	1.966349
H (15)	-0.962380	8.658643	-3.889915	-8.72553	-3.719049	0.860535
C (16)	0.014679	5.766025	-3.666577	-5.806961	-3.583214	-0.05392
C (17)	0.824547	4.668254	-3.818669	-4.695154	-3.776368	-0.835295
H (18)	1.524473	4.589626	-4.640221	-4.62105	-4.611884	-1.518993
C (19)	0.698046	3.656731	-2.861359	-3.663542	-2.841442	-0.702377
H (20)	1.305436	2.765320	-2.941663	-2.760961	-2.953702	-1.287868
C (21)	-3.050700	-1.287423	0.877696	1.053567	0.923765	3.198715
H (22)	-2.865325	-1.202896	-0.186113	1.007438	-0.146038	3.032485
C (23)	-4.222134	-1.837957	1.367312	1.357857	1.45091	4.442459
H (24)	-4.988168	-2.182481	0.686075	1.549243	0.794306	5.280266
C (25)	-4.372310	-1.936030	2.739736	1.401889	2.828385	4.575489
H (26)	-5.269284	-2.364383	3.168876	1.622803	3.284278	5.532356
C (27)	-3.356729	-1.485255	3.566030	1.158019	3.623512	3.46787
H (28)	-3.465717	-1.570784	4.637038	1.177949	4.70009	3.56183
C (29)	-2.211023	-0.938658	2.998437	0.872548	3.015685	2.250926
C (30)	-1.069517	-0.445650	3.831845	0.627571	3.808361	1.008573
C (31)	-1.106620	-0.453823	5.222031	1.009926	5.139846	0.890755
H (32)	-1.972368	-0.803557	5.764178	1.51113	5.653319	1.698437
C (33)	0.000000	0.000000	5.915899	0.7673	5.795191	-0.303391
H (34)	0.000000	0.000000	6.998296	1.072528	6.826007	-0.42947
N (35)	2.080437	0.847743	1.673820	-0.833669	1.65921	-2.085521
C (36)	3.050700	1.287423	0.877696	-1.428782	0.888452	-2.991887
H (37)	2.865325	1.202896	-0.186113	-1.367256	-0.178946	-2.819473
C (38)	4.222134	1.837957	1.367312	-2.105754	1.410074	-4.080836
H (39)	4.988168	2.182481	0.686075	-2.573578	0.748397	-4.797029
C (40)	4.372310	1.936030	2.739736	-2.17384	2.786464	-4.212516
H (41)	5.269284	2.364383	3.168876	-2.707491	3.236942	-5.039842
C (42)	3.356729	1.485255	3.566030	-1.56004	3.587381	-3.263875
H (43)	3.465717	1.570784	4.637038	-1.628301	4.663469	-3.338854
C (44)	2.211023	0.938658	2.998437	-0.885396	2.987218	-2.207714
C (45)	1.069517	0.445650	3.831845	-0.209936	3.792261	-1.147543

C (46)	1.106620	0.453823	5.222031	0.14768	5.121596	-1.340776
H (47)	1.972368	0.803557	5.764178	-0.027057	5.61789	-2.284595
O (48)	-0.417963	-1.629994	-0.976114	1.657849	-0.952519	0.44008
O (49)	1.137153	-2.776933	0.140023	2.806526	0.102342	-1.157023
O (50)	1.519615	-7.081313	-2.685404	7.039257	-2.837019	-1.526013
O (51)	0.042475	-6.893154	-4.430655	6.840995	-4.523625	0.016622
C (52)	0.327733	-2.645483	-0.790542	2.668492	-0.803561	-0.322055
C (53)	0.197241	-3.757989	-1.803768	3.762386	-1.833811	-0.174691
C (54)	1.008579	-4.896029	-1.665217	4.887516	-1.749261	-1.011125
H (55)	1.701097	-4.976911	-0.838650	4.973427	-0.9498	-1.734216
C (56)	0.879265	-5.874745	-2.612688	5.847405	-2.713636	-0.866149
C (57)	1.195248	-7.599431	-3.971794	7.534917	-4.122666	-1.164771
H (58)	2.027308	-7.416577	-4.659778	7.320941	-4.835608	-1.96783
H (59)	0.962380	-8.658643	-3.889915	8.599941	-4.056732	-0.954664
C (60)	-0.014679	-5.766025	-3.666577	5.732594	-3.732378	0.067094
C (61)	-0.824547	-4.668254	-3.818669	4.64769	-3.830857	0.902032
H (62)	-1.524473	-4.589626	-4.640221	4.564633	-4.624926	1.632465
C (63)	-0.698046	-3.656731	-2.861359	3.654984	-2.856521	0.75953
H (64)	-1.305436	-2.765320	-2.941663	2.773566	-2.895465	1.385145

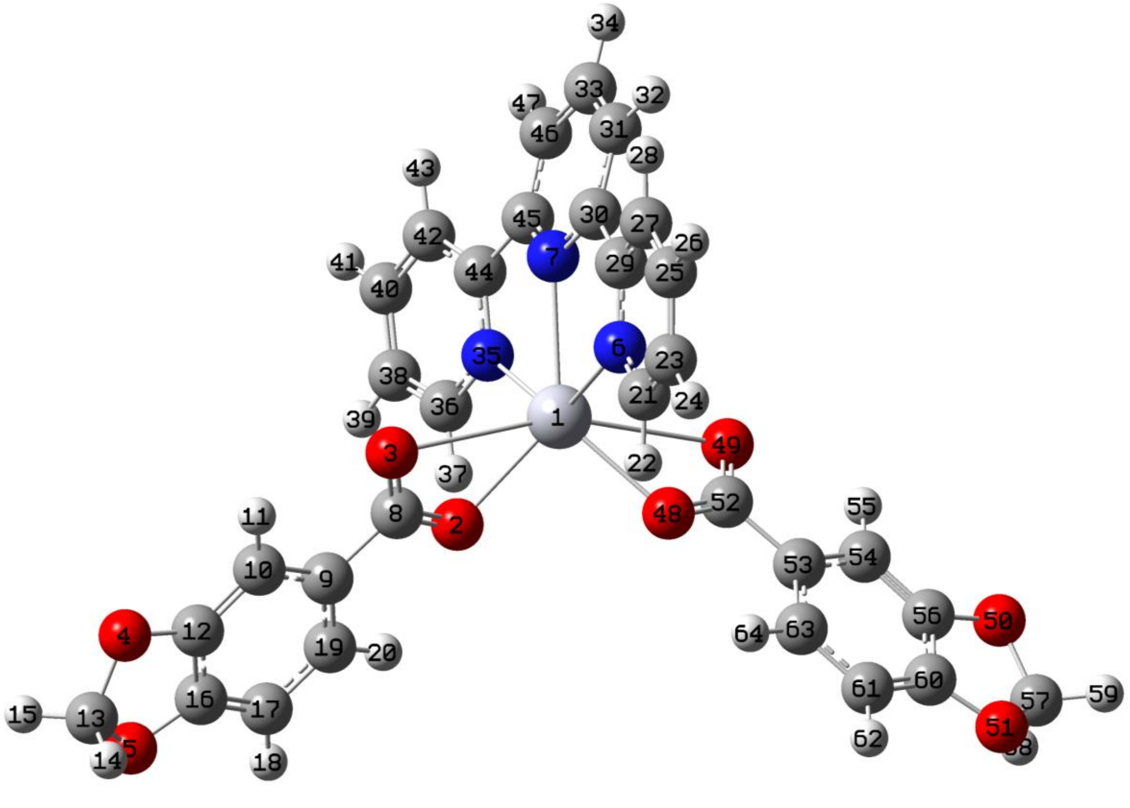

Figure S41. Optimized geometry of **5** in MeOH solution with labelling scheme.
